# A Statistical Mechanical Framework for Predicting Fermi Contact NMR Shifts

**DOI:** 10.1021/jacs.6c07045

**Published:** 2026-08-14

**Authors:** Euan N. Bassey, Elias Sebti, Anton Van der Ven, Raphaële J. Clément

**Affiliations:** † Materials Department, 8786 University of California Santa Barbara, Santa Barbara, California 93106-5050, United States; ‡ Materials Research Laboratory, University of California Santa Barbara, Santa Barbara, California 93106-5050, United States; § Max Planck Institute for Solid State Research, Stuttgart 70569, Germany

## Abstract

Solid-state nuclear magnetic resonance (NMR) spectroscopy is a powerful probe of local chemical environments in functional materials, many of which incorporate paramagnetic transition-metal or rare-earth ions. Unpaired electrons can induce strong electron–nuclear hyperfine interactions, giving rise to large paramagnetic shifts that encode detailed information about the local atomic and electronic structure. These same interactions, however, often produce severe line broadening, complicating spectral assignment—particularly in materials exhibiting mixed valence, magnetic and charge ordering, defects, or compositional disorder. Although first-principles calculations can aid in interpreting paramagnetic shifts, their computational cost becomes prohibitive for structurally and chemically complex systems. This limitation motivates the development of new approaches that retain first-principles accuracy while enabling tractable simulations across disordered, multicomponent materials. Here, we introduce an ab initio framework that combines cluster expansion techniques with Monte Carlo sampling to predict finite-temperature paramagnetic (Fermi contact) shifts. We demonstrate the approach for ^7^Li and ^17^O NMR shifts in the cathode material Li_2_MnO_3_. A magnetic cluster expansion reveals that nearest- and next-nearest-neighbor Mn–O–Li interactions dominate the ^7^Li Fermi contact shift. Extension to ^17^O further uncovers significant long-range contributions mediated by Mn–O–Mn–O pathways, identified here for the first time. This methodology enables first-principles-level predictions of paramagnetic NMR shifts in complex materials containing open-shell species, providing a route to interpreting spectra in real-world systems, including those relevant to energy storage, catalysis, and solid-state lighting.

## Graphical abstract

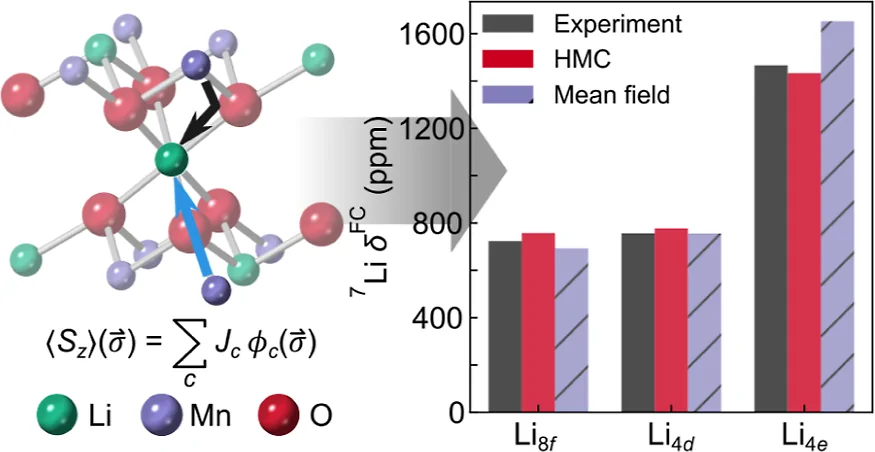

## Introduction

Paramagnetic materials play a key role in a wide range of technologies—including batteries,^1,2^ optical and optoelectronic devices,^3^ sensors,^4^ quantum computing,^5,6^ microprocessors, and catalysts^7,8^ —where functionality arises from open-shell transition metal (TM) or rare earth metal cations. These materials also contain point (antisite defect and/or vacancy), line (dislocation), and/or planar (grain and antiphase boundary, stacking fault) defects, along with cation, anion and/or charge (dis)­ordering. Such structural complexities significantly affect electronic and redox properties and, therefore, device performance.^9–11^ In this regard, techniques sensitive to the short-range atomic and electronic structure of paramagnetic materials can provide unique insights into the local processes that dictate their function and complement long-range methods such as diffraction, imaging and scattering.

Solid-state nuclear magnetic resonance (NMR) spectroscopy is an element-specific technique that probes local chemical environments in both crystalline and amorphous materials through resonant excitation of nuclear spins in an applied magnetic field. The resonant frequency, expressed as a chemical shift δ (in ppm) relative to a reference, reflects interactions between the nucleus of interest and its local environment. As such, the chemical shifts of resonances in an NMR spectrum convey detailed information about the chemical environments present in a material, and when multiple resonances are observed, their integrated intensities correspond to the relative populations of distinct sites.

Paramagnetic solids pose significant challenges for NMR spectroscopy due to the strong hyperfine interactions between unpaired electrons (from *d* or *f* orbitals of transition metal, *TM*, or rare earth species) and nearby nuclear spins.^12,13^ These interactions lead to rapid nuclear relaxation times and the formation of a “blind sphere” around the paramagnetic center, within which NMR signals are severely broadened or unobservable.^13–17^ Nuclei beyond this region often exhibit broad, overlapping resonances, particularly in systems with slow electronic relaxation or strong electron–nuclear coupling.

A key consequence of these interactions is the emergence of large-magnitude paramagnetic shifts, often dominated by the through-bond transfer of electron spin density to the nucleus of interest: this interaction is known as the Fermi contact interaction. Fermi contact shifts can reach hundreds to thousands of ppm—far exceeding those in diamagnetic systems—and encode rich information about the local chemical and magnetic environment. However, their interpretation remains challenging without computationally expensive first-principles methods.

For diamagnetic solids, first-principles calculations of NMR shieldings, shifts, and couplings have become central to NMR crystallography, especially since the development of gauge-including projector augmented wave (GIPAW)-based methods.^18,19^ Recently, machine-learning models such as ShiftML have been developed for the rapid prediction of chemical shifts in molecular solids with near-first-principles accuracy. These models are increasingly used in modern NMR-crystallographic workflows for spectral prediction and assignment, as well as for structural discrimination in pharmaceutical solids.^20–23^

In contrast, predictive modeling of paramagnetic shifts, either as hyperfine-driven shifts in open-shell solids or paramagnetic (excited state) contributions to diamagnetic chemical shift,^13,24^ remains considerably more challenging. The shift of a nucleus in a paramagnetic material not only depends more sensitively on the local structure and the identity of neighboring atoms, but also on fluctuations in the electronic configuration and spin state of nearby paramagnetic centers.^13,25^ Whereas machine-learning models for diamagnetic NMR largely map the local structure directly onto a shielding tensor or shift, paramagnetic shift predictions additionally require an explicit treatment of electron spin degrees of freedom and their fluctuations. Accurately describing the influence of unpaired electrons on the magnetic field experienced by an NMR nucleus remains challenging and typically requires careful assessment of correlated wave function methods, exchange–correlation functionals, and basis sets.^26–30^ As a result, existing approaches for paramagnetic shift prediction remain closely tied to specific chemistries and underlying first-principles protocols, and generally do not provide an explicit treatment of magnetic fluctuations. Therefore, there is a clear need for new computational approaches that can capture magnetic, compositional, and structural complexity, including disorder and defects, while retaining ab initio accuracy.

In this work, we introduce a new framework for representing the Fermi contact shift as a function of temperature and magnetic fluctuations. Furthermore, we describe how the approach can be extended to account for thermal vibrations and chemical disorder. We apply the approach to the Li_2_MnO_3_ cathode material to predict the ^7^Li and ^17^O shifts at finite temperature. The approach relies on cluster expansions of the electronic spin at the nuclei of interest as a function of the surrounding magnetic moments and uses Heisenberg Monte Carlo simulations to sample magnetic fluctuations at finite temperature. The method extends conventional “bond pathway” analyses of Fermi contact shifts, where mean-field approaches are used to determine local contributions to the shift, enabling a more precise decomposition of the effect of magnetic fluctuations. In particular, for ^17^O, we identify previously unresolved next-nearest neighbor contributions.

The new methodology moves beyond previous approaches that rely on a mean field model describing the dependence of the average moment on the paramagnetic sites, ⟨*S*⟩, on surrounding magnetic moments, temperature and applied magnetic field strength,^28,31^ and enables more accurate predictions of the Fermi contact shifts that account for magnetic short-range order. Overall, the framework significantly broadens the applicability of solid-state NMR spectroscopy to complex materials comprising open-shell species, including those that are relevant to energy, solid-state lighting, and catalytic technologies, and extends current computational work on NMR crystallography.^19,32–34^

## Background

In the present work, the term *paramagnetic state* refers to a thermally disordered ensemble of local magnetic moments rather than to a non-spin-polarized electronic state. The open-shell transition metal centers retain finite local moments, but their orientations fluctuate thermally, resulting in the absence of long-range magnetic order.

In this section, we first review the theoretical framework underlying paramagnetic NMR shifts and their prediction from first principles methods. Such approaches rely on a limited set of spin-polarized magnetic configurations and therefore cannot directly capture a thermally fluctuating paramagnetic phase. To overcome this limitation, we introduce a cluster expansion approach as a surrogate model for the dependence of the local hyperfine field on the surrounding magnetic environment, combined with Monte Carlo simulations to sample the thermodynamic ensemble of magnetic microstates at finite temperature. The Fermi contact shift is then obtained as a statistical average over the fluctuating local moment configurations. Finally, we introduce Li_2_MnO_3_ as the model system for this study.

In materials containing 3*d* TM species, the dominant paramagnetic shift typically arises from through-bond Fermi contact interactions between the nuclear spin and nearby unpaired electron spins. Since unpaired electron spins relax much faster (10^–14^ to 10^–6^ s) than nuclear spins (ca. 10^–3^ to 10^3^ s),^13,14^ the nucleus interacts not with the instantaneous electronic magnetic moment, but with its thermal average over all accessible electronic spin states. Consequently, in the fast electronic fluctuation limit (equivalently, a nonentangled electron–nucleus system), the hyperfine interaction produces a *shift* of the nuclear resonant frequency, rather than a resolved hyperfine splitting that would arise from a static electron spin polarization (an entangled electron–nucleus system). The NMR spectrum is recorded on equilibrated systems, so the ensemble-averaged electronic moment equals the thermodynamic average moment with which the nucleus interacts. Contributions from “pseudo-contact” through-space interactions between electronic and nuclear spins^13,30,35–37^ and from bulk magnetic susceptibility effects^38^ are typically at least an order of magnitude smaller for most 3d TM species and are neglected in this work. We note, however, that the restriction to Fermi contact interactions is not fundamental to the cluster expansion framework itself. In principle, the same methodology can be extended to pseudocontact shift contributions, which arise from the coupling between the rank-2 magnetic susceptibility tensor and the anisotropic hyperfine interaction tensor. Provided these quantities can be reliably obtained from first-principles calculations, one could construct cluster expansions for these tensors (with tensorial cluster expansions described in greater detail in the Supporting Information) and thereby evaluate pseudocontact contributions within the same framework. Such extensions could become important for materials containing heavy elements, strong spin–orbit coupling, or highly anisotropic magnetic environments.

### Derivation of the Fermi Contact Shift

The Fermi contact part of the hyperfine interaction Hamiltonian takes the form

1 $${Ĥ}_{hf}=\frac{2}{3}\mu_{0}g_{N}\mu_{N}g_{e}\mu_{B}Ŝ\cdot Î$$

where **Î** is the nuclear spin operator and

2 $$Ŝ=\sum_{i}\delta \left(\vec{r_{i}\;}\right){ŝ}_{i}$$

is the total electron spin operator at the nucleus.^12,24^ The sum in eq 2 extends over all electrons *i* that act on the nucleus of interest, located at positions $\vec{r_{i}\;}$ relative to the nucleus.

Since the electron magnetic moment is ca. ≈700 times larger than that of the proton (and larger still relative to other nuclear moments), electron spins couple more strongly to their environment and relax much faster than nuclei. As such, the electronic spin is effectively time-averaged from the perspective of the nucleus.^24,27,28,31^ This means eq 1 can be written as an effective nuclear Hamiltonian

3 $${Ĥ}_{hf}^{eff.}=\frac{2}{3}\mu_{0}g_{N}\mu_{N}g_{e}\mu_{B}\left\langle \mathrm{S⃗}\right\rangle \cdot Î$$

where ⟨S⃗⟩ is the expectation value of the total electronic spin at the nucleus.

The expectation value ⟨S⃗⟩ is both a quantum mechanical and thermal average. Over the lifetime of nuclear spin excitations, the electronic wave function fluctuates between several eigenstates, Ψ, with populations governed by statistical mechanics. In a given eigenstate, the quantum mechanical expectation value is ⟨S⃗⟩=⟨Ψ|S|Ψ⟩ , while the thermal average is obtained by weighting the quantum averages by their Boltzmann populations. Formally, ⟨S⃗⟩ is expressed via the density operator, $\rho =e^{-\beta Ĥ}$ , as^24^

4 $$\left\langle \mathrm{S⃗}\right\rangle =\frac{Tr\left(\rho S\right)}{Tr\left(\rho \right)}$$

Here, β = 1/*k*_B_ *T* (*k*_B_ is Boltzmann’s constant) and *Ĥ* is the electronic Hamiltonian. Since the Hamiltonian *Ĥ* contains the Zeeman interaction of the electrons with the applied magnetic field, *B*_0_, ⟨S⃗⟩ depends on both temperature and the applied magnetic field.

The expectation value in eq 4 is a three-dimensional vector with Cartesian components ⟨*S*_*x*_⟩, ⟨*S*_*y*_⟩ and ⟨*S*_*z*_⟩. Likewise, the nuclear spin operator is a vector with components *Î*_*x*_, *Î*_*y*_ and *Î*_*z*_. In the high-field limit, the nuclear spins are quantized along *B*_0_,^12,13,24^ conventionally chosen as the *z*-axis. Consequently, only the (longitudinal) *z*-component of **I** is relevant, and the effective Fermi contact contribution to the nuclear Hamiltonian becomes

$${Ĥ}_{FC}^{eff.}=\frac{2}{3}\mu_{0}g_{N}\mu_{N}g_{e}\mu_{B}\left\langle S_{z}\right\rangle {Î}_{z}$$ 5

where *Î*_*z*_ acts on the nuclear wave function.

The Hamiltonian in eq 5 yields a shift in the resonant frequency of the nucleus

6 $$\Delta \nu^{FC}=\frac{2\mu_{0}g_{N}\mu_{N}g_{e}\mu_{B}\left\langle S_{z}\right\rangle }{3h}$$

Relative to the Larmor frequency $\left(\nu_{0}=\frac{g_{N}\mu_{N}}{h}B_{0}\right)$ , this is

7 $$\delta^{FC}=10^{6}\cdot \frac{2\mu_{0}g_{e}\mu_{B}\left\langle S_{z}\right\rangle }{3B_{0}}$$

where the factor 10^6^ scales the shift to the usual parts-per-million (ppm) scale. This shift is therefore a constant and independent of the field strength, *B*_0_, provided that the NMR measurements are performed at a field where ⟨*S*_*z*_⟩ has a linear dependence on *B*_0_. The shift is, however, temperature-dependent, since ⟨*S*_*z*_⟩ is a thermal average. We note that the expression in eq 7 assumes that: (1) the nuclear spin relaxation time is much longer than that of the electrons, such that the nucleus only interacts with the thermal average of the electron spin and; (2) the applied magnetic field is strong enough to quantize the nuclear spins along the field axis, but low enough to ensure that ⟨*S*_*z*_⟩ varies linearly with field strength.

### First-Principles Calculation of the Spin Density at the Nuclear Position

The expectation value of the electron spin density at the nuclear position can be calculated from first-principles with spin-polarized density functional theory (DFT).^39^ The Cartesian components of the spin density at a point *r⃗* can be computed from the Kohn–Sham spinors, ϕ_*i*,σ_ (indices *i* and σ denoting the orbital and spin up/down labels, respectively)^39^

8 $$S_{x}\left(\mathrm{r⃗}\right)=\frac{\hbar }{2}\sum_{i}\left[\phi_{i,↑}^{*}\left(\mathrm{r⃗}\right)\phi_{i,↓}\left(\mathrm{r⃗}\right)+\phi_{i,↓}^{*}\left(\mathrm{r⃗}\right)\phi_{i,↑}\left(\mathrm{r⃗}\right)\right]$$

9 $$S_{y}\left(\mathrm{r⃗}\right)=\frac{\hbar }{2}\sum_{i}\left[\phi_{i,↑}^{*}\left(\mathrm{r⃗}\right)\phi_{i,↓}\left(\mathrm{r⃗}\right)-\phi_{i,↓}^{*}\left(\mathrm{r⃗}\right)\phi_{i,↑}\left(\mathrm{r⃗}\right)\right]$$

10 $$S_{z}\left(\mathrm{r⃗}\right)=\frac{\hbar }{2}\sum_{i}\left[\phi_{i,↑}^{*}\left(\mathrm{r⃗}\right)\phi_{i,↑}\left(\mathrm{r⃗}\right)-\phi_{i,↓}^{*}\left(\mathrm{r⃗}\right)\phi_{i,↓}\left(\mathrm{r⃗}\right)\right]$$

In the high-field limit, only the *z*-component of the spin density, *S*_*z*_, is needed to compute the Fermi contact shift at the nucleus of interest, denoted *X* hereafter. Only orbitals with *s* character will contribute to the local electron spin density at the nucleus, since only these orbitals have nonzero amplitude at the nucleus. This includes doubly occupied *s*-character orbitals, as these can have nonzero spin density at the nucleus *X* via exchange coupling with the valence electrons, inducing a slight difference in the spatial distributions of spin-up and -down components.^12,13^ In the *TM*oxides relevant to this work, distinct magnetic orderings (spin polarizations on the TM sublattice) will generate different local spin density distributions at the nucleus of interest.

The *z*-component of the spin density may be written as the difference in spin-up and -down charge densities^39–41^

11 $$S_{z}\left(\mathrm{r⃗}\right)=\frac{\hbar }{2}\left[\rho^{↑-↓}\left(\mathrm{r⃗}\right)\right]=\frac{\hbar }{2}\left[\rho_{↑}\left(\mathrm{r⃗}\right)-\rho_{↓}\left(\mathrm{r⃗}\right)\right]$$

where

12 $$\rho_{↑}\left(\mathrm{r⃗}\right)=\sum_{i}\phi_{i,↑}^{*}\left(\mathrm{r⃗}\right)\phi_{i,↑}\left(\mathrm{r⃗}\right)$$

with a similar expression for $\rho_{↓}\left(\mathrm{r⃗}\right)$ .

In the high-field limit, the Fermi contact shift (eq 7) can therefore be calculated using only the value of ⟨*S*_*z*_⟩ evaluated at the nucleus. The factor of 1/2 in eq 11 reflects the “per electron” expression introduced by Middlemiss et al.^28^

### The Goodenough–Kanamori–Anderson Rules

The Fermi contact shift, δ^FC^, arises from the transfer of unpaired electron density from the 3*d* orbitals of nearby paramagnetic *TM* cations to the *s* orbitals of the nucleus *X* under study. The resulting unpaired electron spin density at the nuclear position, $\rho^{↑-↓}\left(\mathrm{r⃗}\right)$ (eq 11), drastically increases the size of the local magnetic field experienced by the nuclear spin, and generates a proportionally large shift. The magnitude and sign of the electron spin density transfer from a given TM cation to the nucleus *X*, and the corresponding contribution to δ^FC^, depends on the overlap between and filling of the orbitals involved, resulting in a delocalization- or polarization-type transfer mechanism.^26^ They also depend on the size and orientation of the *TM* magnetic moments. Hence, δ^FC^ is a sensitive probe of the local atomic and electronic structure around *X* but is challenging to interpret.

Early efforts by Grey and co-workers^42–44^ to rationalize the sign and magnitude of Li Fermi contact shifts in paramagnetic lithium transition metal oxide cathodes used the Goodenough–Kanamori–Anderson (GKA) rules^45–48^ to identify competing delocalization- and polarization-type transfer mechanisms.^26,43,44,49^ This approach is often referred to as “bond pathway analysis”. To illustrate this method, we consider here a Mn^4+^–O^2–^–Li^+^ interaction pathway with either a 90° or a 180° angle between the two bonds (Figure 1), and the effect of virtual hopping (exchange) of unpaired electrons between Mn and Li *via* the bridging O atom. In room temperature NMR experiments, the sample is placed in a strong external magnetic field. Although electron moments in the paramagnetic material fluctuate rapidly, the field slightly biases the electronic populations, causing electron spin moments near Mn to preferentially align along the field and generate a small net magnetic moment. This majority spin orientation is conventionally referred to as “spin-up” or “positive” spin density,^26^ ,^40^ ,^43^ ,^44^ ,^49^ without implying a specific electron Zeeman energy.

**Figure 1:**
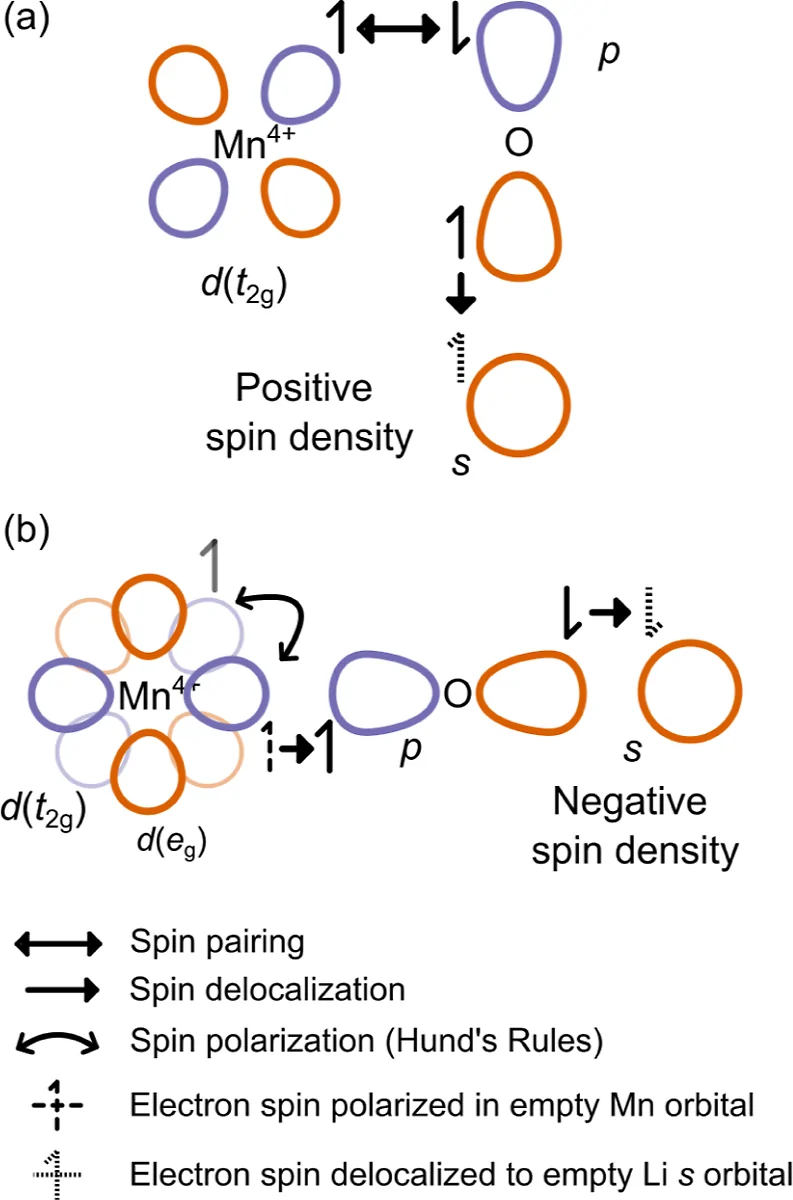
Illustration of unpaired electron spin density transfer from a paramagnetic Mn *d* orbital to the Li nucleus *via* a Mn­(3*d*)–O­(2*p*)–Li­(*s*) bond pathway at (a) a 90° bond angle and (b) a 180° bond angle. According to the Goodenough–Kanamori–Anderson rules, the sign and magnitude of the shift contributions depend on the geometry of the interaction pathway.

In the case of a 90° Mn^4+^–O^2–^–Li^+^ interaction pathway, the Mn *t*_2g_ orbital overlaps side-on (π-type overlap) with the O 2*p* orbital (Figure 1a). The two electrons that occupy the Mn–O orbital must be spin-paired (double-headed arrow, Figure 1a); consequently, the electron circulating near Mn is polarized “up”, while that near O is polarized “down”. The same O 2*p* orbital also interacts with the Li *s* orbital. According to Pauli’s principle, the second electron in the O 2*p* orbital interacting with Li must be polarized “up”, resulting in a net “up” electron density delocalized onto the Li *s* orbital and, consequently, at the Li nucleus (Figure 1a). Overall, for a 90° Mn–O–Li bond pathway, spin density transfer from a paramagnetic Mn *d* orbital to the Li nucleus occurs through a delocalization-type mechanism involving a single O 2*p* orbital, resulting in the transfer of like spin density and a positive shift contribution.

If, instead, the angle between the Mn–O–Li angle is 180°, the Mn *t*_2g_-orbital does not have the correct symmetry to overlap with the O 2*p*-orbital pointing at both Mn and Li (Figure 1b). In this case, the Mn *t*_2g_ orbital polarizes the vacant and orthogonal Mn *e*_g_ orbital (double-headed curved arrow in Figure 1b), whose symmetry permits overlap with the relevant O 2*p* orbital, such that electrons circulating in the Mn *e*_g_ orbital and the adjacent O 2*p* lobe are polarized “up”, according to Hund’s rules. Therefore, the electron that hops between the Li *s* orbital and the second lobe of the O 2*p* orbital (pointing toward the Li *s* orbital) is polarized “down”. This “down” polarization can then delocalize onto the Li *s* orbital, and therefore the Li nucleus. Overall, for a 180° Mn–O–Li bond pathway, spin density transfer from Mn to the Li nucleus occurs through a polarization-type mechanism involving two orthogonal 3*d* orbitals on Mn, leading to transfer of opposite spin density and a negative shift contribution.

We emphasize that this approach to predicting Fermi contact shift contributions is inherently qualitative, but it nonetheless provides a useful guideline for estimating the contributions of paramagnetic centers to the shifts of nearby ions. However, its applicability becomes limited when ion displacements are significant and when bond angles deviate from the ideal 90 or 180° cases, or when multiple magnetic species contribute to the shift at a given site. A more quantitative approach relies on first-principles methods to compute shifts, as described next.

### Mean-Field Models to Determine ⟨*S*_*z*_⟩

At thermal equilibrium, a solid will sample many microstates. In NMR spectroscopy, it is useful to distinguish between “fast” and “slow” degrees of freedom—*i.e.*, those that equilibrate on timescales that are much shorter and much longer, respectively, than the nuclear spin relaxation times. In *TM* oxides at room temperature, fast electronic fluctuations are dominated by reorientations of *TM* magnetic moments, leading to changes in orbital occupancy via the exchange and superexchange interactions. Likewise, vibrations perturb orbital overlap and hence local spin density distributions around nuclei. As such, the expectation value ⟨S⃗⟩ in eq 7 is a thermal average over magnetic and vibrational fluctuations. “Slower” degrees of freedom such as the degree of chemical disorder—requiring diffusive hops in a solid—generally evolve slowly and can be treated as static on the NMR time scale (except for highly mobile species^50,51^ ).

Evaluation of the Fermi contact shift therefore requires computing the spin density at nucleus *X* for a range of magnetic and vibrational configurations, followed by statistical averaging according to eq 4. However, exhaustive sampling of all configurations is intractable, and a surrogate model is needed to interpolate spin densities from first principles calculations performed on a finite set of magnetic orderings.

In a simple mean-field approximation, the electronic spin density at the nucleus of interest scales linearly with the magnetization, *M*, of the *TM* cations of the solid

13 $$\left\langle S_{z}\right\rangle ={\left\langle S_{z}\right\rangle }_{sat.}\frac{M}{M_{sat}}$$

where ${\left\langle S_{z}\right\rangle }_{sat.}$ is the electron spin density at the nucleus in the ferromagnetic state (which may be evaluated using DFT) and *M*_sat_ the saturation magnetization. This model neglects the effect of magnetic short-range ordering on the electron spin density at the nuclear position and will predict the same value of ⟨*S*_*z*_⟩ (and hence the Fermi contact shift) for configurations with different distributions of local moments but the same total moment, *M*. As such, eq 13 fails to account for local magnetic fluctuations.

Previous authors have used a Curie–Weiss mean-field model (where *k*_B_ *T* ≫ *g*μ_B_ *B*_0_) to relate the average magnetization of the solid to the applied field and temperature^28^

14 $$M\left(T,B_{0}\right)=\frac{B_{0}\mu_{eff}^{2}}{3k_{B}\left(T-\theta \right)}$$

where μ_eff_ and θ are the effective magnetic moment and Weiss constant, respectively. This yields the following expression for the Fermi contact shift

15 $$\delta^{FC}=10^{6}\cdot \frac{2{\left\langle S_{z}\right\rangle }_{sat}\mu_{eff}^{2}}{9k_{B}S_{form}\left(T-\theta \right)}$$

where *S*_form_ is the formal spin of the transition metal ions surrounding the nucleus of interest. *S*_form_ is related to the per-ion saturation magnetization by *M*_sat_ = *g*μ_B_ *S*_form_. This expression has yielded reasonably accurate shift predictions (within 10–20% of the experimental shift^27,31,52–54^ ) for paramagnetic materials in which exchange interactions are weak and the magnetic moments are not correlated at the experimental temperature.

### A Cluster Expansion of ⟨S⃗⟩

To capture short-range magnetic order beyond a linear dependence on the average magnetization, we model the electron spin density at a nucleus *X* using a cluster expansion.^55–59^ For collinear (Ising-like) magnetic moments, each magnetic site, *l*, is assigned an occupation variable σ_*l*_ = ±1 (representing “up” and “down” spin states), which may be written as a vector $\mathrm{\sigma ⃗}=\left(\sigma_{1},\sigma_{2},...,\sigma_{N}\right)$ , to keep track of the occupants on all *N* magnetic sites. The expectation value of the electron spin at the nucleus *X* is then expanded as

16 $$\left\langle S_{z}\right\rangle \left(\mathrm{\sigma ⃗}\right)=\sum_{c}J_{c}\Phi_{c}\left(\mathrm{\sigma ⃗}\right)$$

where Φ_*c*_ = ∏_*i*∈*c*_σ_*i*_ are cluster basis functions, corresponding to products of spins on clusters of sites *c* such as points, pairs, triplets, etc. (a simple example is provided in Figure 2). The coefficients *J*_*c*_ are known as effective cluster interactions (ECIs) and describe the weighting of the cluster function Φ_*c*_ in the expansion, and may be trained using first-principles calculations of ⟨*S*_*z*_⟩ for different magnetic configurations, σ⃗.^60^

**Figure 2:**
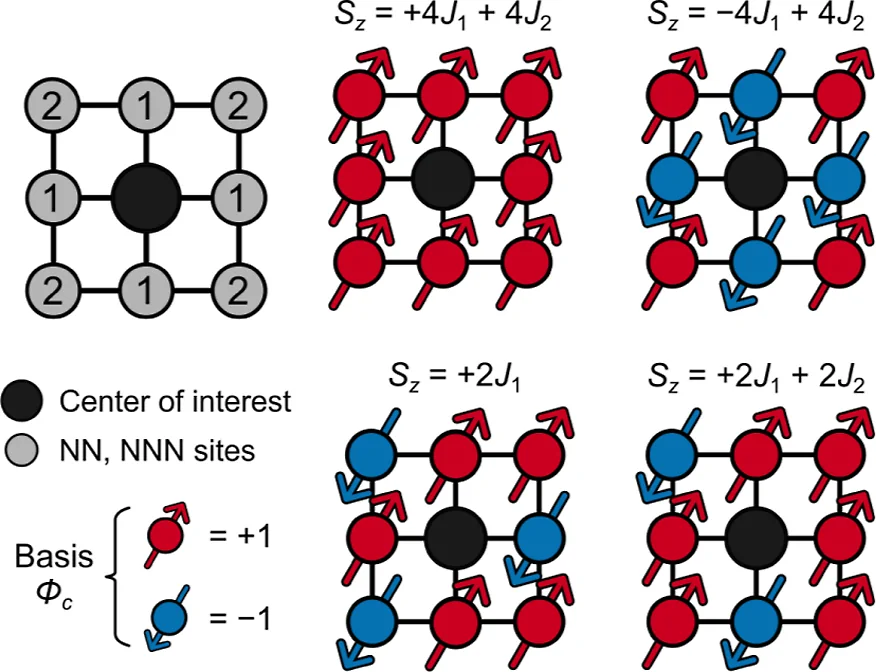
A simple example of a magnetic cluster expansion of *S*_*z*_. Left: a two-dimensional square lattice, showing symmetrically distinct nearest neighbors (NN, “1”) and next-nearest neighbors (NNN, “2”), together with the Ising (spin up/spin down) basis set Φ_*c*_. Right: four symmetrically distinct magnetic configurations and their corresponding *S*_*z*_ values expressed in terms of the basis functions and expansion coefficients, *J*_*c*_, here restricted to point-term contributions to the effective Hamiltonian.

Crystal symmetry reduces the number of independent ECIs. Since eq 16 is a cluster expansion of a local property, it must be invariant to all space group symmetry operations that map the nucleus onto itself.^61–63^ The collection of *N*_p_ symmetrically equivalent cluster functions belong to an orbit, $\Omega_{p}=\left(\Phi_{c_{1}},...,\Phi_{c_{N_{p}}}\right)$ and have the same ECI. As such, eq 16 may be rewritten as

17 $$\left\langle S_{z}\right\rangle \left(\mathrm{\sigma ⃗}\right)=\sum_{\Omega_{p}}J_{p}N_{p}\xi_{p}\left(\mathrm{\sigma ⃗}\right)$$

where

18 $$\xi_{p}\left(\mathrm{\sigma ⃗}\right)=\frac{1}{N_{p}}\sum_{c\in \Omega_{p}}\Phi_{c}\left(\mathrm{\sigma ⃗}\right)$$

are known as correlation functions and are averages over symmetrically equivalent cluster functions for a specific configuration, σ⃗.^64,65^ These correlation functions can serve as symmetry-adapted fingerprints of the local magnetic ordering and may be used as descriptors in a neural network model^66^ of ⟨*S*_*z*_⟩ if nonlinear surrogate models are desired.

Further restrictions on the cluster basis functions are imposed by time-reversal symmetry:^57^ when all spins in an Ising configuration σ⃗ are flipped, the sign of ⟨*S*_*z*_⟩ must also flip. Hence, only cluster bases containing an odd number of spin products (i.e., points, triplets, ...) survive. We note that the cluster expansion constructed later is restricted to Ising-type magnetic moments. Additional details on cluster expansions constructed from the computed shift tensors are provided in the Supporting Information, where we further discuss the possibility of extending the framework to include additional degrees of freedom, namely lattice displacements and chemical disorder.

### Monte Carlo Simulations

To evaluate the thermal averages of ⟨*S*_*z*_⟩, we perform Heisenberg Monte Carlo simulations at the experimentally relevant temperature and magnetic field strength. Two types of cluster expansions are used. The first is a spin cluster expansion of the energy^57,58,67–69^ as a function of the orientations of the magnetic moments of the paramagnetic centers. The second is a local cluster expansion of *S*_*z*_ at each symmetrically distinct site of the species *X* of interest as a function of neighboring Mn magnetic moments (eq 17). A large number of magnetic microstates is sampled using the spin cluster expansion of the energy, as described in ref 69. These configurations are then used to calculate thermal averages of $\left\langle S_{z}\right\rangle$ at each site. Since the value of *S*_*z*_ at a given site depends on the local orientations of nearby magnetic moments, it varies across the crystal. To obtain a representative shift, we therefore average the shifts for species *X* over all sites in the cell.

### Li_2_MnO_3_ as a Model System

Li_2_MnO_3_, containing paramagnetic Mn^4+^ ions, was selected as a model system to demonstrate the cluster expansion method for Fermi contact shift predictions. This compound also holds technological importance as the end-member of the Li- and Mn-rich family of energy-dense oxide cathodes.^70–74^

The crystal structure of Li_2_MnO_3_, synthesized via a standard solid-state reaction (see Experimental), was determined from Rietveld refinement of room temperature powder neutron diffraction data, as shown in Figure S1. We henceforth refer to this model as N-Li_2_MnO_3_, with space group *C*2/*c*. The structure consists of alternating layers of edge-sharing cation octahedra (Figure 3a): one layer contains only LiO_6_ octahedra, the other a honeycomb-ordered arrangement of MnO_6_ and LiO_6_ octahedra resulting in Mn_6_ rings. We note that our choice of space group differs from other reports, where the structure adopts a *C*2/*m* structure.^75,76^ Here, we chose to use the *C*2/*c* structure to capture the effects of stacking faults, as per previous reports.^77,78^

**Figure 3:**
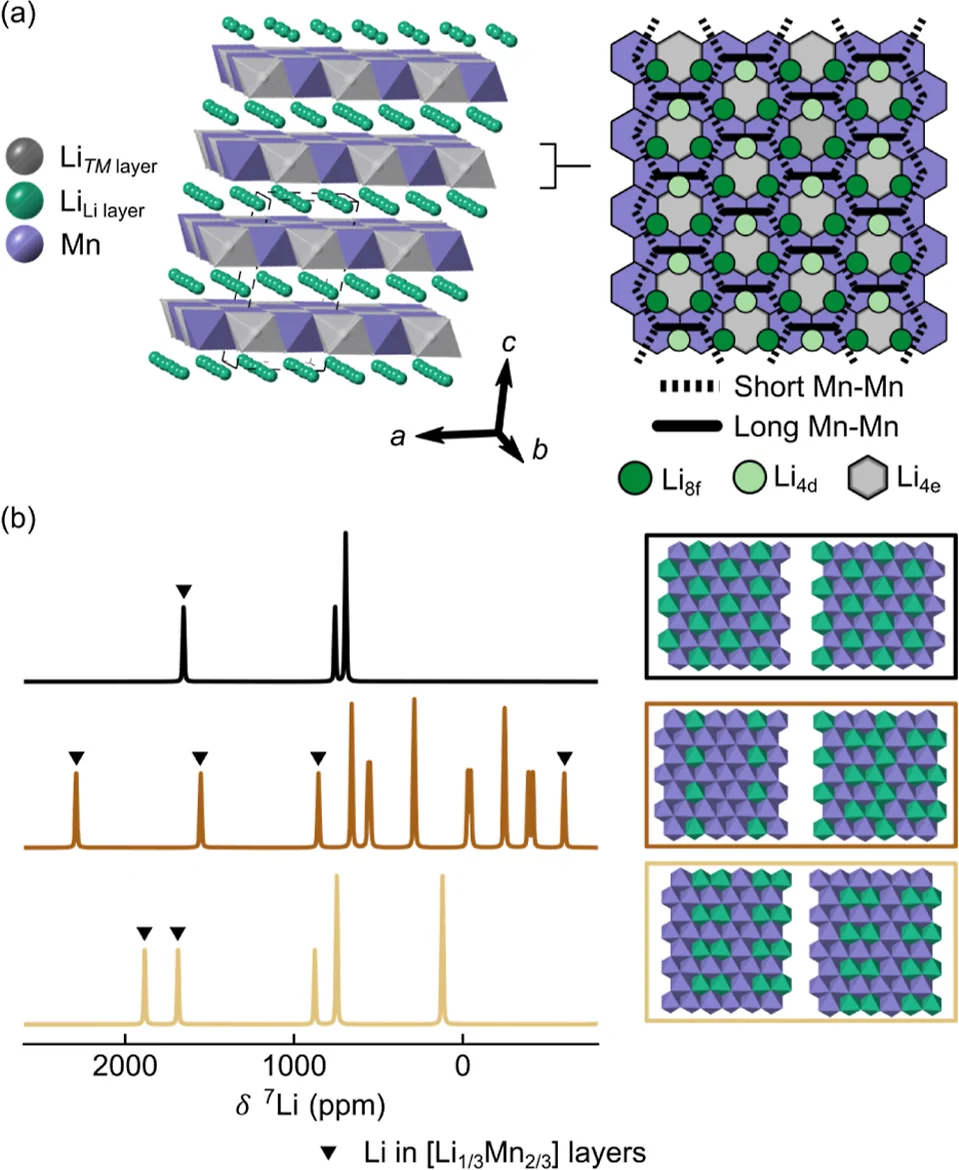
Impact of the Li/Mn ordering in N-Li_2_MnO_3_ on the predicted ^7^Li NMR spectrum. (a) Refined crystal structure of Li_2_MnO_3_ obtained from Rietveld refinement of powder neutron diffraction data (N-Li_2_MnO_3_). On the right, a top-down view of the layers, highlighting the “pinched” Mn–Mn bond distances in the [Li_1/3_Mn_2/3_] layer and the relative positions of the three symmetrically distinct Li sites: Li_8*f*_ and Li_4*d*_ in the Li layer, and Li_4*e*_ in the [Li_1/3_Mn_2/3_] layer. The latter is depicted as a gray hexagon, as it occupies the center of a six-membered ring of Mn cations in the honeycomb-ordered lattice. (b) Simulated spectra based on first-principles calculated shifts for three distinct Li/Mn orderings within the [Li_1/3_Mn_2/3_] layers (shown on the right). All first-principles ^7^Li Fermi contact shifts were scaled using an appropriate magnetic scaling factor (eq 15).

In the literature, it is referred to as an “O3” structure,^75,76,79^ following the nomenclature system introduced by Delmas and co-workers.^80^ The Mn_6_ rings are “pinched” and feature two long Mn–Mn bonds and four short Mn–Mn bonds, similarly to other honeycomb-structured “Li-rich” layered oxide cathodes where Li and transition metals occupy the same layer.^81–83^ Mn_6_ ring pinching and the three crystallographic Li sites in Li_2_MnO_3_ are illustrated in Figure 3a, with Li_8*f*_ and Li_4*d*_ in the Li layers, and Li_4*e*_ in the [Li_1/3_Mn_2/3_] layers. Prior reports have indicated that Li_2_MnO_3_ exhibits stacking faults, where the honeycomb pattern between Li and Mn is misaligned between adjacent layers,^76,79^ as well as antisite defects, where Mn and Li species swap positions in the otherwise ordered [Li_1/3_Mn_2/3_] layer.^79^ Our refinement clearly shows the presence of antisite defects, with a finite probability of Li occupying Mn sites and Mn occupying Li sites within the TM layers. Low-angle reflections diagnostic of stacking faults (for example, the (002) reflection^76^) are difficult to observe in the neutron diffraction pattern, complicating the determination of the stacking fault density.

The ^7^Li solid-state NMR spectrum recorded at 300 K on Li_2_MnO_3_ is shown in Figure S2 and consists of three distinct signals. The two resonances at 724 and 757 ppm correspond to Li_8*f*_ and Li_4*d*_ environments in the Li layers,^49^ respectively, with their relative intensities reflecting the site multiplicities (the Li_8*f*_ resonance accounts for 48% of the integrated signal intensity, while the Li_4*d*_ resonance accounts for 24%). The third resonance at 1466 ppm, from Li_4*e*_ in the [Li_1/3_Mn_2/3_] layers,^49^ accounts for 28% of the integrated signal intensity. We note that the high-frequency resonance must be fit with two components (a representative single-component fit is shown in Figure S2 of the Supporting Information): the first corresponding to the expected honeycomb-like LiMn_6_ local environment, and the second corresponding to either a LiMn_5_Li local environment,^84^ or stacking faults of the O3 structure.^75,85^ The first (LiMn_6_) component accounts for approximately 22% of the total integrated signal intensity, while the second component accounts for about 5%.

## Results

We now apply the approach to calculate finite-temperature Fermi contact shifts described in the Background section to ^7^Li and ^17^O in Li_2_MnO_3_. Prior to doing so, we evaluate the effect of chemical site occupancy on the Fermi contact shift by performing first principles ^7^Li shift calculations on representative Li_2_MnO_3_ structural models. Our results indicate that a perfectly honeycomb-ordered structure adequately represents the experimental system. We therefore restrict the subsequent analysis to this model, allowing us to neglect site occupancy as a variable.

### Impact of Chemical Disorder on the Fermi Contact Shift

We begin by examining the role of chemical site occupation on the mean-field, first principles ^7^Li Fermi contact shifts. We focus on the experimental N-Li_2_MnO_3_ structure and enumerate several Li/Mn orderings within the [Li_1/3_Mn_2/3_] layers, while keeping the layer stoichiometry, ion positions, and lattice parameters fixed.

Figure 3b shows three representative Li/Mn configurations along with their simulated spectra. For each configuration, we first calculate the unpaired electron spin density at the ^7^Li nuclear positions (and thus the Fermi contact shift via eq 7) using the CRYSTAL23 software package^40,41,86^ with the Hyb20 (B3LYP) exchange–correlation functional. Room-temperature shifts are then estimated using the mean-field approach described in the Background section, i.e., by scaling the ferromagnetic unpaired electron spin densities at the Li sites by a magnetic scaling factor derived from eqs 13 and 15. Because our objective is to isolate and assess the impact of site occupation, we do not explicitly treat magnetic fluctuations using a cluster expansion formalism. Instead, the mean-field description provides a consistent framework for comparing shifts across different Li/Mn orderings without introducing additional complexity from coupled magnetic–structural degrees of freedom. The magnetic scaling factor is obtained from bulk magnetic susceptibility measurements (Figure S3), using an effective magnetic moment of $\mu_{eff}=3.87\;\mu_{B}\;mol_{Mn}^{-1}$ and a Weiss constant of θ = −45 K, in agreement with a previous report.^75^

Additional results obtained using a modified hybrid functional containing 35% Fock exchange (Hyb35) are provided in the Supporting Information (Figures S4, S6, S8, S10–S12, S14, S16–S20). Typically, for paramagnetic 3d transition metal oxides, the shifts predicted by Hyb20 and Hyb35 calculations span a range that encompasses experimentally observed values.^27,28,31^ For clarity, the main text focuses on Hyb20 results, as these show closer agreement with the experimental shifts than those obtained with Hyb35.

In Figure 3b, the top (black) spectrum was computed for the experimental N-Li_2_MnO_3_ structure (without structural relaxation, preserving the honeycomb ordering of Li and Mn within the TM layers). The lower spectra were calculated for models with different Mn/Li distributions, as illustrated to the right of the spectra. The wide distribution of predicted chemical shifts for Li in both the Li layers and the [Li_1/3_Mn_2/3_] layers in the three configurations indicates that the local chemical environment has a strong impact on the Fermi contact shift. In the nonhoneycomb-ordered configurations, Li environments with similar numbers and arrangements of Mn and Li neighbors to those in the honeycomb-ordered structure yield calculated shifts comparable to those obtained for honeycomb-ordered N-Li_2_MnO_3_.

Overall, the honeycomb-ordered structural model shows the best agreement with the experimental spectrum (Figures S2 and 3). Given that the occupancy of nearby sites by transition metals is a “static” degree of freedom on the NMR time scale, it should not be incorporated into the averaging of ⟨*S*_*z*_⟩. Accordingly, we restrict the remainder of the analysis to the honeycomb-ordered model, which allows us to disregard chemical disorder as a variable.

### Impact of Magnetic Configurations on the Value of ⟨*S*_*z*_⟩

In this section, we investigate the effect of different configurations of the Mn magnetic moments on *S*_*z*_ at the Li nuclear positions in honeycomb-ordered N-Li_2_MnO_3_. Several symmetrically distinct collinear magnetic configurations were generated using CASM.^59^ For each configuration, the unpaired electron spin density at the Li nuclear positions was computed using CRYSTAL23 and the Hyb20 functional.

The spin density at the Li nuclear position is plotted as a function of the total spin of the surrounding Mn cations, *S*_tot._, in Figure 4 (and in Figure S4 for Hyb35 results). For each Li site, *S*_tot._ is defined as the sum of the spins of all Mn ions within a radius of 4.2 Å. This cutoff includes nearest- and next-nearest-neighbor (NN and NNN) Mn spins for Li_8*f*_ and Li_4*d*_, and only NN Mn spins for Li_4*e*_. In this summation, Mn spins are treated as either “spin-up” (*s* = +3/2) or “spin-down” (*s* = −3/2).

**Figure 4:**
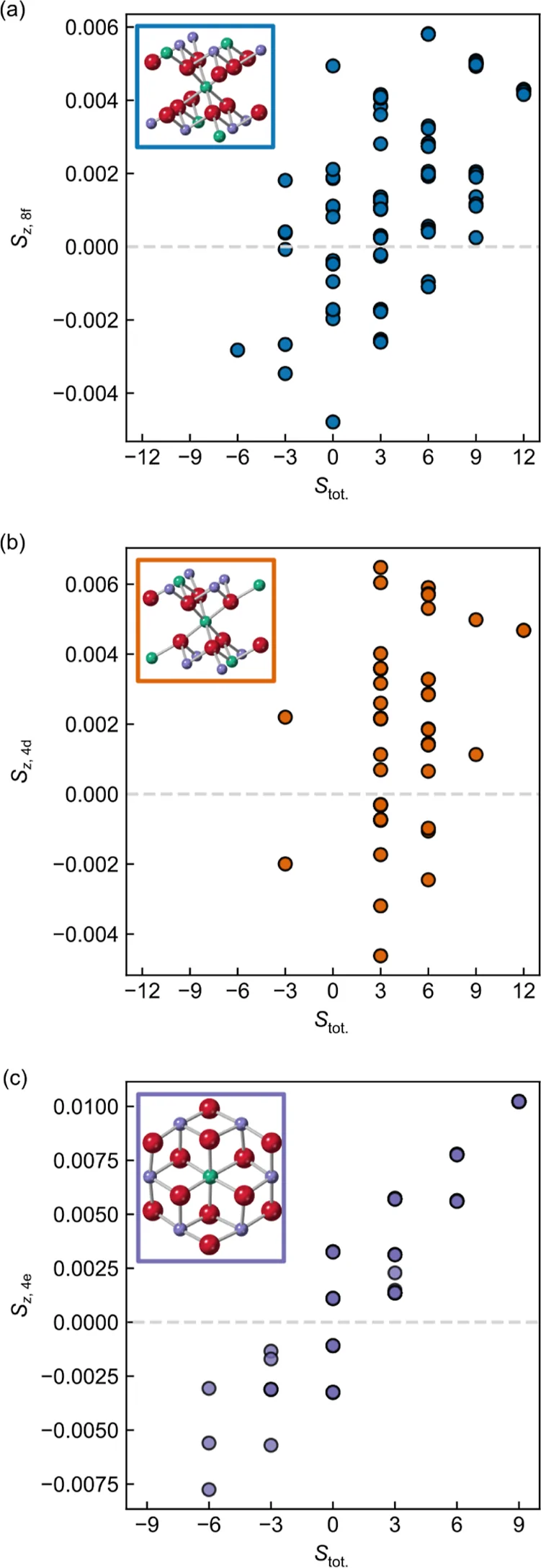
*z*-Component of the total spin, *S*_*z*_, of Li ions for various magnetic environments of (a) Li_8*f*_, (b) Li_4*d*_, and (c) Li_4*e*_ in N-Li_2_MnO_3_, plotted against the total spin, *S*_tot._ of the Mn cations surrounding the Li sites, up to a maximum radius of 4.2 Å. The nonlinear dependence of *S*_*z*_ on *S*_tot._ suggests that a cluster expansion is necessary.

Based on the mean-field approximations used previously,^27,28^ *S*_*z*_ is expected to vary linearly with the magnetization of the surrounding TM cations (eq 13). However, our results show that the dependence of *S*_*z*_ on the total spin around a given Li site depends on the local contributions from symmetrically distinct TM cations. This motivates the use of a cluster expansion to quantitatively account for each of these contributions when evaluating the Fermi contact shift.

### Cluster Expansion of ⟨*S*_*z*_⟩

The dependence of *S*_*z*_ at a nucleus of interest on the orientations of surrounding magnetic moments can be described using a local cluster expansion described by eq 17. The expansion is parametrized using *S*_*z*_ values calculated for different collinear (Ising-like) magnetic configurations of the Mn cations. To this end, cluster basis functions centered on symmetrically distinct Li sites in N-Li_2_MnO_3_ were determined using CASM.^59^ For each magnetic configuration, the corresponding averages of the symmetrically distinct cluster basis functions (eq 18) were also calculated using CASM.

When fitting the effective Hamiltonian, we retained the minimum number of clusters required to achieve a satisfactory fit. In practice, only point clusters (terms associated with the magnetic spin orientation of individual Mn ions), and only terms corresponding to Mn ions located two bonds away from the Li site, were necessary. This corresponds to nearest and next-nearest neighbor (NN and NNN, respectively) interactions for Li_8*f*_ and Li_4*d*_, but only NN for Li_4*e*_. For the latter site, including NNN terms in the effective Hamiltonian did not significantly improve the fit, yielding similar rmse and cross validation scores (Figure S5). Accordingly, we focus on the NN-only fit for Li_4*e*_. Hence, all relevant bond pathways are of the type Mn^4+^–O–Li. These terms are hereafter referred to as pair contributions or pair terms, as they represent the coupling between the orientation of a single magnetic moment on Mn, *s*_*i*_, and the unpaired electron spin at the ^7^Li nucleus. Notably, the effective cluster interaction (ECI) associated with each pair term is analogous to the bond pathway interaction between a Mn center and a nearby Li nucleus, representing the coupling between the unpaired electron spin density transferred from the Mn to the Li nucleus and the Li nuclear spin.

As shown in Figure 5a–c, our cluster expansion fits are excellent for all three local Li environments in N-Li_2_MnO_3_; further, our predicted values of *S*_*z*_ closely match the calculated *S*_*z*_ for both the training and testing data sets. The resulting root-mean-square errors (rmses) for the fits are on the order of 10^–5^, corresponding to approximately 0.1% of the target span. Relative to the span of calculated values, this is substantially smaller than the error levels typically reported for other cluster expansions and machine-learned interatomic models.^87–89^ This level of accuracy is considered excellent, considering that our final model contains a small number of terms (up to eight) after fitting to over 170 data points.^67,87^ The small errors likely also reflect the strong dependence of *S*_*z*_ on local magnetic interactions. The Hyb35 results likewise show an excellent fit, with only small errors (Figure S6).

**Figure 5:**
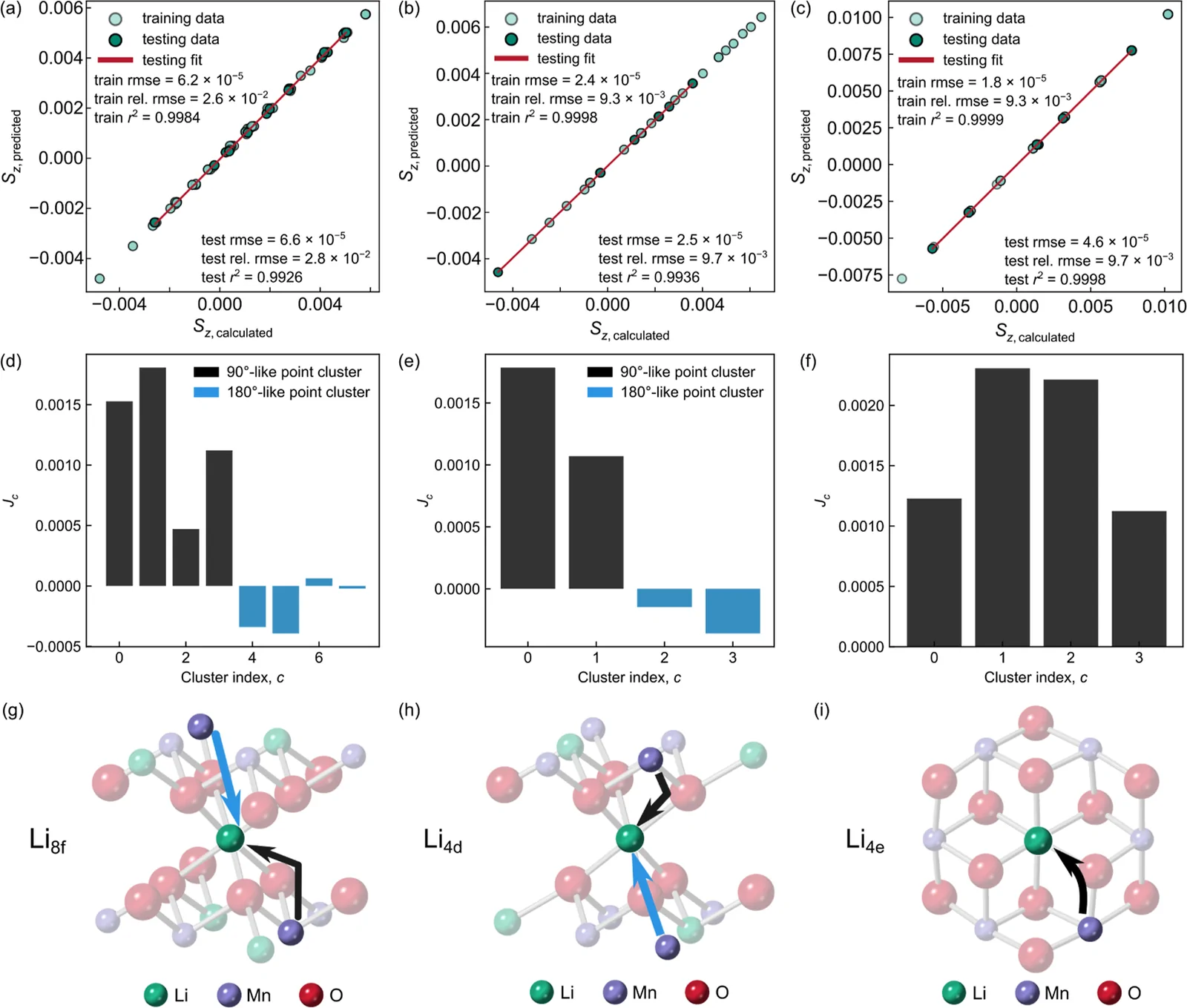
Cluster expansion results of the unpaired electron spin density, *S*_*z*_, at Li_8*f*_, Li_4*d*_, and Li_4*e*_ nuclear positions in N-Li_2_MnO_3_. (a–c) show cluster expansion fits, and (d–f) corresponding effective cluster interactions (ECIs), for Li_8*f*_, Li_4*d*_, and Li_4*e*_, respectively. The root-mean square error (rmse), *r* ^2^ and relative rmse were computed using *k*-fold cross-validation for both the training and testing data. Relative rmse values, defined as the rmse normalized by the standard deviation of the calculated *S*_*z*_, of ∼10^–2^ to 10^–3^ indicate an excellent predictive accuracy of the cluster expansion. (g–i) show the interaction pathways between the Li nucleus and neighboring Mn centers associated with each ECI type for Li_8*f*_, Li_4*d*_, and Li_4*e*_, respectively.

For Li_8*f*_ and Li_4*d*_, two types of ECIs can be distinguished: those corresponding to NN Li-Mn interactions ((Li–Mn)_NN_), and those corresponding to NNN Li–Mn interactions ((Li–Mn)_NNN_). The (Li–Mn)_NN_ couplings comprise two approximately 90° angle Li–O–Mn interaction pathways. These pathways have large, positive (Li–Mn)_NN_ ECIs, consistent with GKA predictions of a transfer of “up” spin density to the Li nucleus and a positive contribution to the shift (Figures 5d,e, 1a, and S6). Li–O–Mn_NN_ bond angles and Li–O and O–Mn bond lengths are all subtly different, even in the relatively high symmetry N-Li_2_MnO_3_ structure, which leads to a different ECI for each (Li–Mn)_NN_ interaction pathway. In particular, one of the (Li–Mn)_NN_ ECIs for Li_8*f*_ (cluster 2 in Figure 5d) is significantly smaller in magnitude than the others.

The (Li–Mn)_NNN_ couplings comprise one approximately 180° angle Li–O–Mn interaction pathway. The corresponding ECIs are significantly smaller in magnitude than (Li–Mn)_NN_ ECIs. For both Li_4*d*_ and Li_8*f*_, the (Li–Mn)_NNN_ ECIs are either negative or close to zero. Again, these results are consistent with GKA predictions that an approximately 180° Li–O–Mn_NNN_ interaction should yield a small, negative shift contribution. The near-zero (Li_8*f*_–Mn)_NNN_ ECIs (clusters 6 and 7 in Figure 5d) can be rationalized by longer Mn–O and Li–O bonds and slight deviations from a 180° interaction pathway. The (Li–Mn)_NN_ ECIs for Li_4*e*_ in the [Li_1/3_Mn_2/3_] layers are also large and positive, and on average slightly larger than those of Li_8*f*_ and Li_4*d*_, which we attribute to shorter Li_4*e*_–Mn_NN_ distances.

### Prediction of Room-Temperature ^7^Li Fermi Contact Shifts

The finite temperature Fermi contact shifts of ^7^Li in Li_2_MnO_3_ were computed from thermally averaged unpaired electron spin densities, $\left\langle S_{z}\right\rangle$ , obtained for all 4096 Li sites in an 8 × 8 × 8 supercell of the primitive Li_8_Mn_4_O_12_ structure using magnetic Monte Carlo simulations under a constant applied field *B*_0_. The simulations employed a Heisenberg magnetic Hamiltonian,^69^ with exchange parameters fitted to the total energies of 43 collinear magnetic configurations. Within each sampled magnetic configuration, the unpaired spin density at each Li nucleus was evaluated using a local cluster expansion for *S*_*z*_. Further details on the magnetic energy cluster expansion fits are provided in Supporting Note 1.

The first-principles calculations predict that the magnetic ground state of N-Li_2_MnO_3_ consists of nearest-neighbor Mn spins within each [Li_1/3_Mn_2/3_] layer aligned antiparallel, with nearest-neighbor Mn spins in adjacent [Li_1/3_Mn_2/3_] layers also aligned antiparallel. This magnetic ground state is consistent with previous experimental reports^90,91^ and with the magnetic energy cluster expansion, which reveals antiferromagnetic interactions between nearest-neighbor Mn spins along both the short and long Mn–Mn distances within the [Li_1/3_Mn_2/3_] layers (Figures S7 and S8). The Heisenberg Monte Carlo (HMC) simulations predict a magnetic ordering temperature below 1 K, whereas the experimental transition temperature is significantly higher, at 36 K (Figures S9 and S10).^90^ We attribute this discrepancy to the omission of spin–orbit coupling, single-ion anisotropy, and/or spin–spin dipolar interactions in our Monte Carlo model, which have been previously reported for Li_2_MnO_3_.^90^ However, this limitation does not affect the validity of the Monte Carlo predictions at temperatures well above the transition, as N-Li_2_MnO_3_ remains firmly in the paramagnetic regime at room temperature.

As described in the Background, the cluster expansion for *S*_*z*_ was parametrized using collinear magnetic configurations, such that the cluster basis functions depend only on the Mn spin moments projected along the external field direction (i.e., Ising spin variables). In the finite-temperature simulations, however, magnetic configurations were sampled using a continuous Heisenberg Hamiltonian. We adopted Heisenberg statistics as they are expected to more accurately capture the temperature dependence of the magnetic response than Ising statistics.^69^

The local collinear cluster expansion of *S*_*z*_ was elevated to a nonlinear cluster expansion for $\mathrm{S⃗}=\left(S_{x},S_{y},S_{z}\right)$ using the collinear ECI (Figure 5d–f). The value of *S*_*z*_ was then collected at each site inside the Monte Carlo simulation cell and converted into a shift using eq 17. We reiterate that here we focus on the *z*-component of the spin because the system is treated in the high-field limit, where the nuclear spin is effectively quantized along the direction of the applied magnetic field (i.e., along *z*).^12,13,24^

Figure 6a shows histograms of the sampled *S*_*z*_ values for each of the three crystallographic Li sites at 320 K. Those values were obtained from spin densities calculated using the Hyb20 functional. A temperature of 320 K approximates the effective sample temperature during the room-temperature NMR experiment, taking into account frictional heating under fast (60 kHz) magic angle spinning.^51,92^ Each value of *S*_*z*_ represents the unpaired spin density at a Li site within a specific local magnetic environment, determined by the arrangement of neighboring Mn moments through the local cluster expansion. In reality, however, electron spins fluctuate rapidly on the NMR time scale.^13,25^ To account for dynamic averaging, ensemble-averaged ⟨*S*_*z*_⟩ values are computed for each of the three crystallographic Li sites by sampling the distribution of local magnetic environments. This is performed within a large Monte Carlo cell, in which all relevant magnetic arrangements are represented. The ensemble-averaged values are then converted into average Fermi contact shifts using eq 7. The average shifts for Li_8*f*_, Li_4*d*_, and Li_4*e*_ are plotted as vertical lines over the experimental NMR spectrum in Figure 6b. They are also decomposed into contributions from each (NN and NNN) coordination shell in Figure 6c.

**Figure 6:**
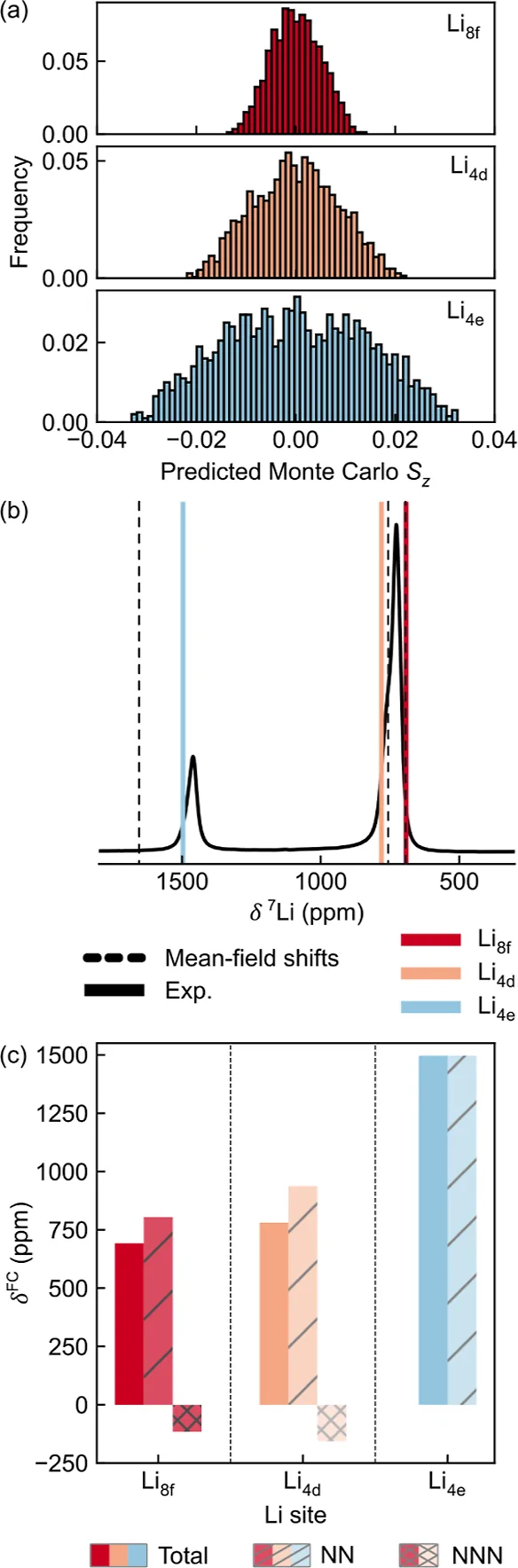
Predicted electron spin densities and ^7^Li Fermi contact shifts from Heisenberg Monte Carlo (HMC) simulations of N-Li_2_MnO_3_ at 320 K and 2.35 T. (a) Histograms of the *z*-component of the unpaired spin density, *S*_*z*_, for Li_8*f*_ (top), Li_4*d*_ (middle), and Li_4*e*_ (bottom) sites in an 8 × 8 × 8 supercell of the primitive Li_8_Mn_4_O_12_ structure. (b) Numerical averages of the Li_8*f*_, Li_4*d*_, and Li_4*e*_ shifts are shown alongside the mean-field calculated shifts (obtained using eq 15), and the experimental spectrum, acquired at 2.35 T with a magic angle spinning frequency of 60 kHz, corresponding to a sample temperature of 320 K. (c) Decomposition of the average shifts into contributions from each coordination shell.

The average HMC shifts calculated for Li_8*f*_, Li_4*d*_, and Li_4*e*_ are in excellent agreement with experiment and correctly reproduce the observed ordering: (δ­(Li_8*f*_) < δ­(Li_4*d*_) < δ­(Li_4*e*_)). Deviations between mean-field and HMC predicted shifts and experimental values are summarized in Figure 7, and Table S1, with mean-field results shown as dashed lines in [Fig fig6]b. Although the mean-field approach captures the magnitude of the shifts, it significantly overestimates the separation between inequivalent sites. In contrast, the HMC results show much closer agreement with experiment.

**Figure 7:**
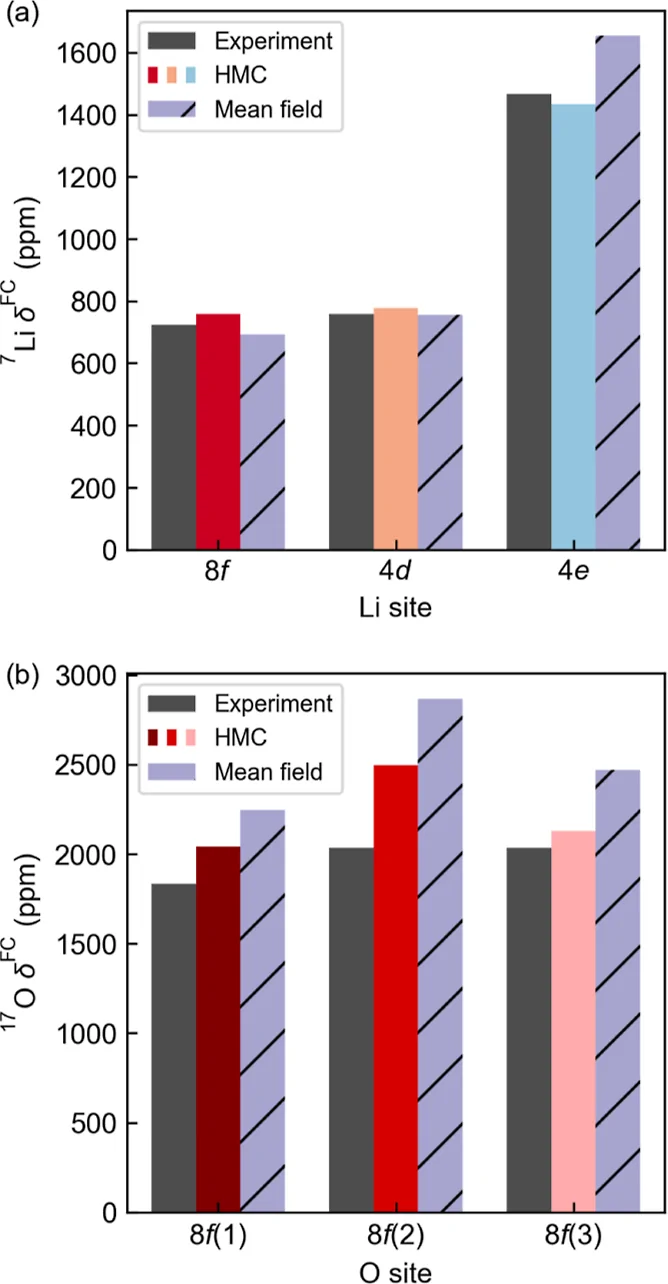
Comparison of experimental and calculated Fermi contact shifts, δ^FC^, for (a) ^7^Li and (b) ^17^O in Li_2_MnO_3_. Experimental shifts were obtained at 60 kHz MAS (320 K) and at a field of 2.35 T (^7^Li) and 11.7 T (^17^O). Heisenberg Monte Carlo (HMC) and mean field (MF) shifts were obtained from first principles calculations on N-Li_2_MnO_3_ using the Hyb20 functional. HMC shifts were determined by combining ECIs from a magnetic cluster-expansion with Monte Carlo-derived Heisenberg Mn moment at 320 K. MF shifts were obtained by scaling the first principles shifts from 0 to 320 K according to eq 15 (θ = −45 K, $\mu_{eff}=3.87\;\mu_{B}\;{mol}_{Mn}^{-1}$ ).

### Prediction of ^17^O Fermi Contact Shifts

While ^7^Li was used as a representative nucleus in the preceding sections, the cluster expansion approach is broadly applicable to a wide range of nuclei. To illustrate this versatility, we examine ^17^O Fermi contact shifts in N-Li_2_MnO_3_. Owing to the closer proximity of oxygen to paramagnetic centers in transition metal oxides, ^17^O NMR shifts are larger in magnitude than ^7^Li shifts and are significantly more sensitive to subtle variations in the local crystal and electronic structure.^75,93^ This heightened sensitivity complicates spectral assignment based on simple bond pathway models; for example, prior work has shown that nominally similar Mn^4+^–O pathways can give rise to a broad distribution of hyperfine contributions spanning several hundred ppm.^75,93^

The N-Li_2_MnO_3_ structure comprises three crystallographically distinct O environments, each occupying an 8*f* Wyckoff position. We denote these sites as 8*f*(1), 8*f*(2) and 8*f*(3), distinguished by their positions relative to the long and short Mn–Mn distances (Figure 8a). Although additional O environments associated with stacking faults have been reported,^75,94^ these are not considered here for the sake of simplicity.

**Figure 8:**
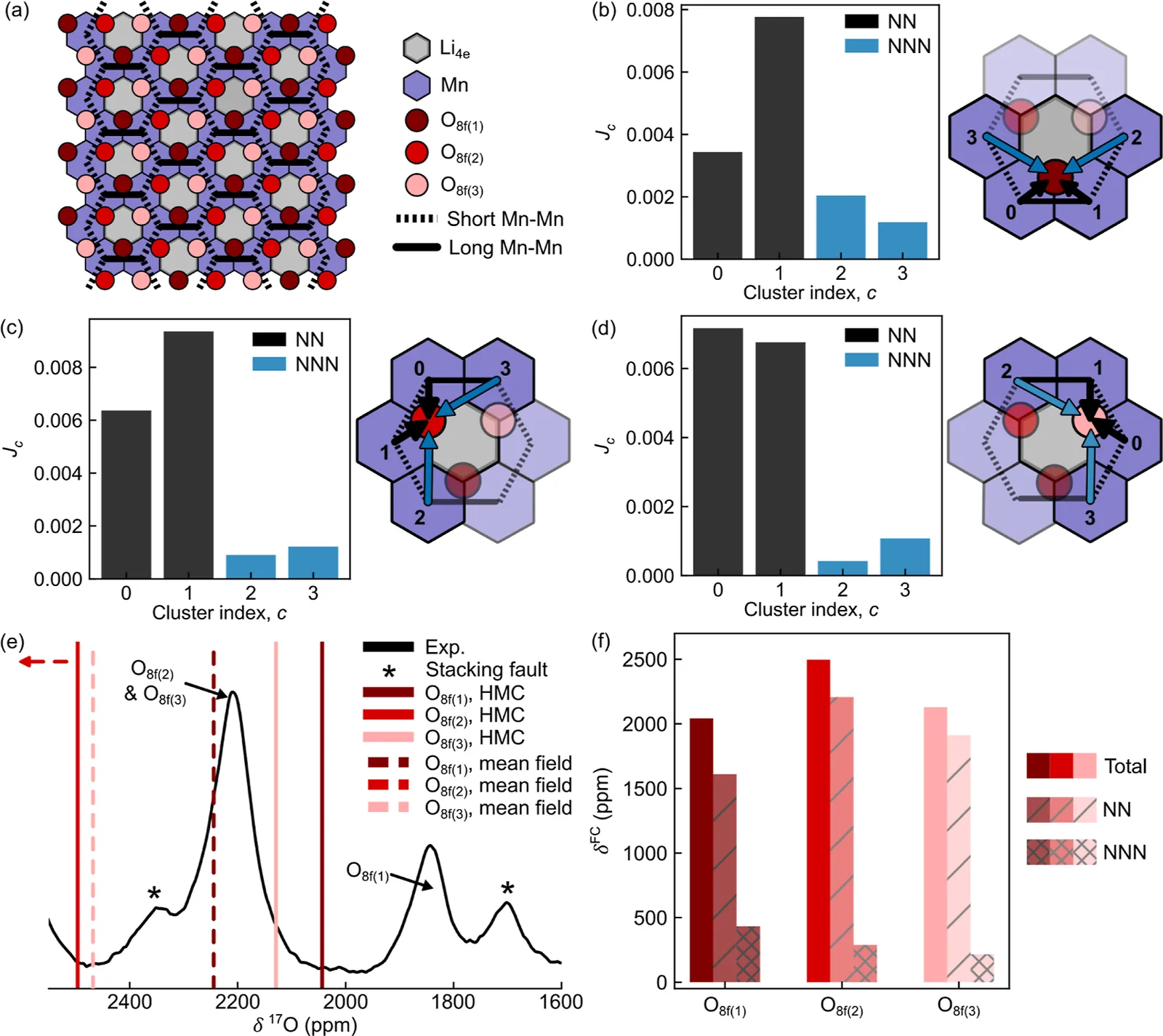
^17^O Fermi contact shift cluster expansion results for the three O crystallographic environments in N-Li_2_MnO_3_. (a) O_8*f*(1)_, O_8*f*(2)_, and O_8*f*(3)_ environments superimposed on the [Li_1/3_Mn_2/3_] layer. (b–d) Effective cluster interactions (ECIs), and interaction pathways between the O nucleus and neighboring Mn centers associated with each ECI type, for O_8*f*(1)_, O_8*f*(2)_ and O_8*f*(3)_, respectively. (e) Numerical averages of the O_8*f*(1)_, O_8*f*(2)_, and O_8*f*(3)_ shifts predicted from Heisenberg Monte Carlo (HMC) simulations at 320 K and 11.7 T, alongside the isotropic region of the experimental spectrum acquired at 11.7 T with a magic angle spinning frequency of 60 kHz, corresponding to a sample temperature of 320 K. Both the HMC predicted shifts (solid vertical lines) and mean-field shifts (dashed vertical lines) have been included. The mean-field shift for O_8*f*(2)_, at 2869 ppm, falls outside the spectral window shown here. The experimental spectrum is reproduced from ref 75. Copyright 2016 the American Chemical Society. (f) Decomposition of the average ^17^O Fermi contact shifts into contributions from each coordination shell.

Using the mean-field approach (eq 15), we obtain shifts of 2245 ppm, 2869 ppm and 2468 ppm, for O in 8*f*(1), 8*f*(2) and 8*f*(3) sites, respectively.

The cluster expansion for ⟨*S*_*z*_⟩, given in eq 17, was fitted to *S*_*z*_ values at ^17^O nuclear positions computed for the same set of collinear Mn spin configurations used for ^7^Li. We find that accurately reproducing the shifts for all three O sites requires four terms: two arising from the first coordination sphere (nearest neighbors, NN), and two from the second coordination sphere (next-nearest neighbors, NNN, Figure S12). As expected, NN contributions are largest in magnitude, reflecting efficient spin density transfer from Mn centers directly bonded to O (Figures 8b–d and S13). However, the cluster expansion fit also clearly reveals that NNN Mn terms make a significant contribution to the ^17^O Fermi contact shift, indicating that the shift is not solely governed by spin density transfer from NN Mn, as previously assumed.^93^ There are two pathways that the Mn NNN spin density may traverse: (i) Mn_NNN_–O–Mn_NN_–O*, and/or (ii) Mn_NNN_–O–Li_NN_–O*, where O* denotes the oxygen nucleus of interest. We note that the former is shorter in distance than the latter and is expected to dominate the NNN spin density transfer mechanism. If the NNN terms correspond to pathway (i), this suggests that the relative alignment of the two Mn magnetic moments within the Mn–O–Mn–O* cluster affects the spin density at O*. The two Mn can adopt four magnetic configurations—up–up, up–down, down–up, down–down—each of which, according to the GKA rules, will lead to a distinct contribution to the spin density at O*. Capturing these differences requires the inclusion of both NN and NNN cluster terms, which together enable to distinguish between the four magnetic configurations.

Finite-temperature ^17^O Fermi contact shifts were computed for all 6144 O sites in the 8 × 8 × 8 supercell of Li_8_Mn_4_O_12_ used in the Monte Carlo simulations. As described previously, for each O site, the *z*-component of *S⃗* was calculated using eq 17 and the ECIs shown in Figure S14 for Hyb20 and Figure S15 for Hyb35. Ensemble-averaged shifts for ^17^O in 8*f*(1), 8*f*(2) and 8*f*(3) sites at 320 K are plotted as vertical lines over the experimental spectrum in Figure 8e. Experimental and HMC-predicted shifts are in relatively good agreement, indicating that the cluster expansion method is not only transferable to other nuclei, but is also capable of capturing longer-range spin density transfers that may be overlooked in simple bond pathway models. Deviations between the mean-field and HMC predictions and experimental values are summarized in Figure 7b, with mean-field results shown as dashed lines in Figure 8e. As in the case of ^7^Li, the mean-field approach reproduces the overall magnitude of the ^17^O Fermi contact shifts but overestimates the differences between the three O environments. In contrast, the HMC results show much closer agreement with experiment, particularly for 8*f*(1) and 8*f*(3). In Figure 8f, the decomposition of the average shift into contributions from each coordination shell highlights that NNN Mn each contribute between 220 and 430 ppm, which represents a significant fraction (between 11 and 21%) of the total ^17^O Fermi contact shift. While unsurprising, these NNN contributions to the shift have not been reported previously.

### Impact of Volume and Atomic Displacements on the Fermi Contact Shift

In the previous sections, we employed a magnetic cluster expansion to obtain the unpaired spin density, ⟨*S*_*z*_⟩, at the ^7^Li and ^17^O nuclei. To examine the influence of cell volume on the calculated ^7^Li and ^17^O Fermi contact shifts, we generated a series of structures based on N-Li_2_MnO_3_ in which the fractional atomic coordinates were held fixed while the unit cell volume was systematically expanded and contracted. The resulting variation is shown in Figure 9a for ^7^Li and in Figure S16 for ^17^O. In both cases, a clear correlation is observed between the unit cell volume and the average Fermi contact shift: increasing the cell volume (and hence the Mn–O and Li–O bond lengths) reduces the magnitude of the shifts, consistent with weaker orbital overlap and, consequently, reduced spin density transfer. Furthermore, the shifts vary smoothly with cell volume, without any abrupt discontinuities, suggesting that the underlying spin density transfer mechanism remains unchanged, while its strength varies continuously with the interatomic distances. In particular, the Li_4*e*_ site is more strongly affected by changes in volume than the Li_8*f*_ or Li_4*d*_ (Figures 9a and S17). This is expected, as the Li_4*e*_ sites lie closer to the paramagnetic Mn centers and therefore couple more strongly to Mn. In addition, all three O sites show similar changes in shift with volume (Figure S16), as expected given their comparable interactions with nearby Mn centers.

**Figure 9:**
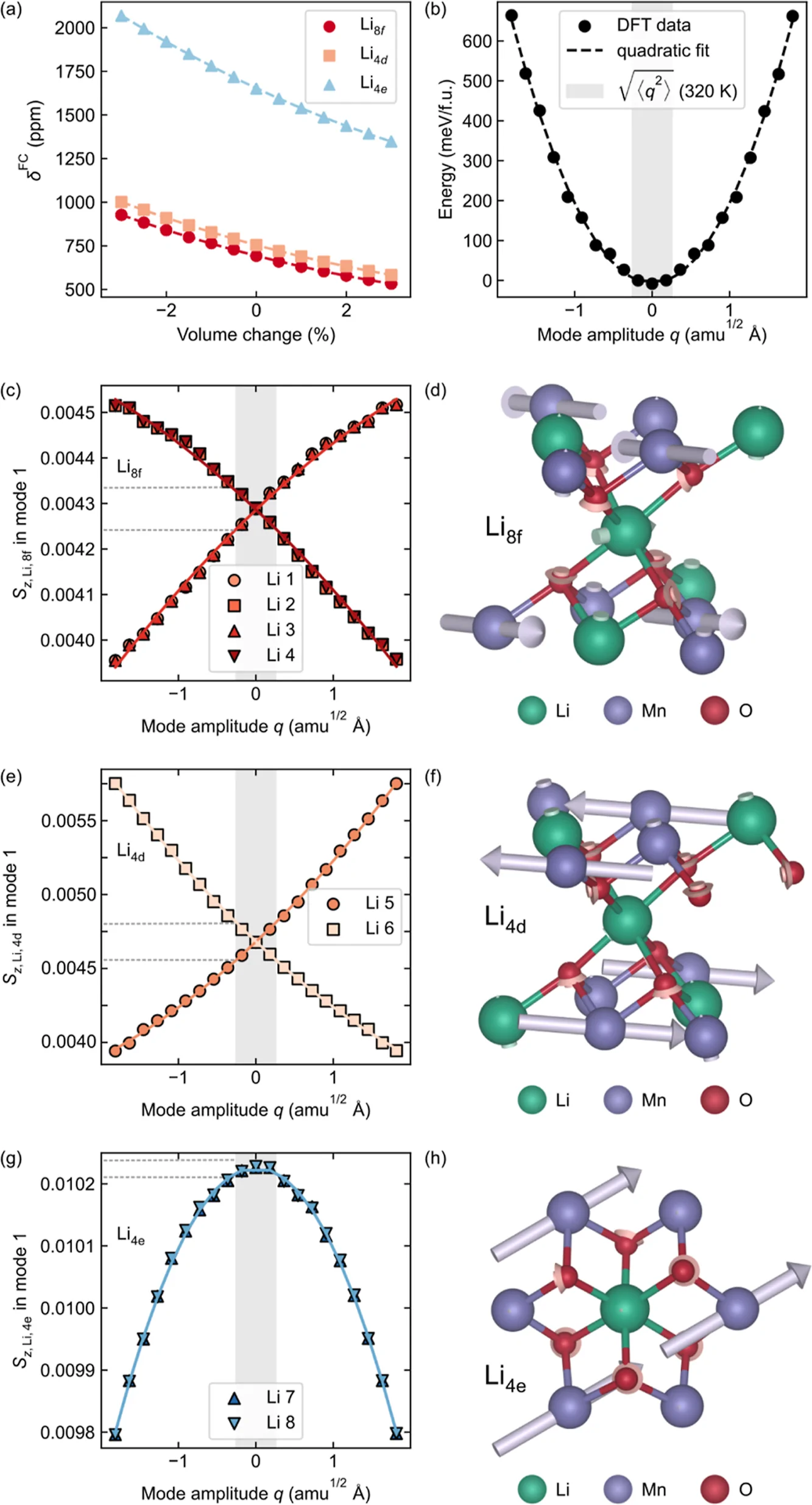
Impact of volume and atomic displacements (vibrational modes) on the calculated ^7^Li *z*-component of spin density, *S*_*z*_. All first-principles ^7^Li spin densities were computed using the Hyb20 functional. (a) Change in Li *S*_*z*_ as a function of cell volume for the N-Li_2_MnO_3_ structure. (b) Variation in the energy per formula unit with normal mode displacement amplitude for mode 1, as described in the main text. The corresponding variations in *S*_*z*_ of Li_8*f*_, Li_4*d*_ and Li_4*e*_ sites along the same mode are shown in (c,d), (e,f), and (g,h), respectively. The light gray boxes in (b,c,e,g) indicate the displacement range expected at room temperature, with the corresponding range of *S*_*z*_ values highlighted with horizontal gray dashed lines.

We now investigate the sensitivity of the Fermi contact shift with respect to nonmagnetic degrees of freedom in honeycomb-ordered Li_2_MnO_3_ (Figure 9). Since vibrational excitations are anticipated to be orders of magnitude faster than magnetic (vibrations fluctuate around 10^12^ to 10^13^ Hz; magnetic fluctuations approach 10^8^ to 10^12^ in the paramagnetic regime^13^), we choose to focus on the impact of vibrations on the spin density at the nuclear position, *S*_*z*_, rather than the shift. This allows us to isolate and assess the effect of vibrations, with a view toward incorporating these contributions into future extensions of the cluster expansion formalism. As such, to fully account for the impact of vibrations on the Fermi contact shift, we would need to first average the shifts from all vibrational modes using Bose–Einstein statistics, and then average across magnetic fluctuations. This would require a coupled vibrational–magnetic cluster expansion, which is beyond the scope of the present paper. We evaluate hereafter variations in *S*_*z*_ as a function of displacement amplitudes for select vibrational modes (Figure 9).

To isolate the impact of displacements, *i.e.*, lattice vibrations, we choose to enumerate three symmetry-adapted normal modes (excluding rigid translations) of N-Li_2_MnO_3_ using CASM,^59^ and compute ^7^Li shifts for a series of (mass-normalized) amplitudes. In this scheme, the individual phonon modes provide a basis for systematically perturbing the spin-density transfer pathways, enabling quantification of their individual contributions to the Fermi contact shift. We chose to explore three modes in particular, which we label mode 1, mode 2 and mode 3 for clarity. In mode 1, shown in Figure 9b–h, Mn ions dominate the motion, with every other Mn ion around the Mn_6_ rings in the [Li_1/3_Mn_2/3_] layers moving in the same direction within a given layer, while the displacement direction alternates between adjacent [Li_1/3_Mn_2/3_] layers. The effect of the Mn displacement is to shear the Mn_6_ honeycomb ring and elongate two of the short Mn–Mn distances and shorten the long Mn–Mn distance (Figure S17). Accompanying this, the O ions move to effectively compress the [Li_1/3_Mn_2/3_] layer and widen the Li layer, and the Li_8*f*_ ions displace by a small amount in a direction perpendicular to the Mn displacement vector.

For mode 2, presented in the Supporting Information (Figures S17–S24), displacements are dominated by O ions: O on either side of each [Li_1/3_Mn_2/3_] layer move in opposite directions, causing expansion of the [Li_1/3_Mn_2/3_] slab height (i.e., along the *c*-axis) and contraction of the Li layer slab height. The Li and Mn ions experience negligible displacements in mode 2. Mode 2 was therefore chosen to isolate the influence of distortions on the O sublattice. Mode 3 is similar to mode 1, but Mn ions instead move in a direction parallel to the vector between two nearest neighbor Mn ions in the [Li_1/3_Mn_2/3_] layer. We also depict the modes in terms of the distributions of Li–O, Mn–O and Li–Mn distances, as well as Mn–O–Li bond angles in Figures S25–S30 inclusive.

The dependence of the total energy of the cell versus the amplitude of mode 1 is presented in Figure 9b. The energy varies quadratically with displacement, indicating that the system remains within the harmonic regime over the considered range. For mode 1, the fitted force constant is 400 meV amu^–1^ Å^–2^ (amu: atomic mass units), giving a vibrational frequency of 9.89 THz (330 cm^–1^) and a mass-scaled root-mean-square displacement at room temperature of 0.254 amu^1/2^ Å. In the harmonic regime, if *S*_*z*_ varies linearly with the normal mode displacement amplitude, it is not expected to produce a net change in the shift (see Supporting Information, Section S14). In contrast, quadratic or higher-order dependencies of the shift on the displacement result in a nonzero average shift at room temperature.

The variation in the shift of Li atoms occupying Li_8*f*_ sites is shown in Figure 9c. The variation in *S*_*z*_ is approximately linear over the range of amplitudes considered for mode 1, changing by up to 7.8% relative to the equilibrium value at one of the displacement extrema. This variation arises from changes in the individual contributions of the neighboring Mn atoms to the Li spin density as the ions are displaced. Across the room-temperature root-mean-square displacement (±0.254 amu^1/2^ Å), *S*_*z*_ for Li_8*f*_ varies linearly, and as such, vibrations are not expected to contribute to the total *S*_*z*_ and hence the total shift.

For Li_4*d*_, a linear trend is observed at small displacements, whereas a slight curvature emerges at larger displacement amplitudes, presumably due to nonlinear orbital overlap effects (Figure 9e). The values of *S*_*z*_ over the full range of normalized mode amplitudes shown in Figure 9e span a wider range than those for Li_8*f*_, reaching a maximum deviation from the equilibrium shift of 173 ppm (22.8%). This behavior indicates that the shift of Li_4*d*_ is more sensitive to atomic displacements than Li_8*f*_. We tentatively suggest that the origin of the greater sensitivity of Li_4*d*_ is due to the slightly larger range of local Mn–O–Li angles sampled over mode 1. One possible explanation for the increased sensitivity relative to Li_8*f*_ is the different orientation of the Mn displacement vectors with respect to the Mn–O–Li bonds (Figure 9f). Despite the larger sensitivity, this vibrational mode is again not expected to make a net contribution to the shift at room temperature.

The *S*_*z*_ of Li_4*e*_ varies quadratically with displacement (Figure 9g), indicating that vibrations associated with this symmetry-adapted mode are expected to contribute to the observed shift. However, the calculated contribution at room temperature is only 0.0001 spin density units (see Section S14 of the Supporting Information), and is therefore at least 1 order of magnitude smaller than the contribution from magnetic fluctuations.

The changes in the ^17^O *S*_*z*_ for mode 1 are shown in Figure S18. While the magnitude of the variation exceeds that of ^7^Li (reaching up to 40% relative to the equilibrium value), *S*_*z*_ remains linear for this mode and is therefore not expected to contribute to the finite-temperature shift. Further data for two other additional modes are provided in Figures S19–S24.

The results presented in this section demonstrate that the Fermi contact shift is sensitive to variations in bond lengths and angles along the spin density transfer pathways. However, since magnetic fluctuations away from the ferromagnetic configuration generally reduce the magnitude of the Fermi contact interaction, the present approach provides an upper bound on the vibrational contribution to the observed shifts. We therefore suggest that, although vibrations of the symmetry-adapted mode considered here do contribute, their effect is significantly smaller than that of magnetic fluctuations. It would nevertheless be valuable in future work to include vibrational averaging and spin–phonon coupling, particularly in materials with softer lattices or stronger magnetostructural coupling, where vibrational effects are expected to be more pronounced.

## Discussion

In this work, we have developed an ab initio-based framework for predicting finite-temperature Fermi contact shifts that explicitly incorporates magnetic fluctuations and provides a route toward including additional (e.g., vibrational) degrees of freedom. Our work extends current NMR crystallography methods to periodic paramagnetic solids, providing a framework to explore competing degrees of freedom and their impact on the experimental NMR spectrum. Using ^7^Li in Li_2_MnO_3_ as an example, we demonstrate that this approach yields room-temperature Fermi contact shifts that reproduce experimental trends and are in close quantitative agreement with experiment. We go beyond a simple mean-field description of the magnetization of neighboring TM sites,^27,28,31,54,95^ instead employing a cluster expansion approach combined with Monte Carlo sampling of magnetic configurations to accurately capture short-range magnetic order. While long-range magnetic order is absent at room temperature in Li_2_MnO_3_ (*T*_N_ ≈ 35 K, Figure S3), local electronic spin fluctuations remain important, as each Li site samples a distribution of neighboring Mn spin orientations. This local description, and the ability to resolve distinct magnetic environments, is expected to be particularly powerful in correlated magnetic materials such as α-NaMnO_2_,^52,96,97^ where bond pathway decomposition is challenging and the simple mean-field scaling factor must be modified.^52^

The differences between the Heisenberg Monte Carlo (HMC)-predicted shifts (using eq 7) and experimental shifts for the three Li environments in Li_2_MnO_3_ (Figure 7a) are substantially smaller than those obtained using a mean-field (MF) approach (eq 15). The improvement is especially pronounced for the Li_4*e*_ site (Figure 6b). The Li_8*f*_ and Li_4*d*_ shifts are already well described at the mean-field level, and the HMC-derived shifts show comparable agreement with experiment relative to the MF results.

The broader applicability of the method is illustrated by examining the ^17^O Fermi contact shifts in Li_2_MnO_3_. These shifts are significantly more sensitive to changes in the local environment than those of ^7^Li, and the agreement between HMC-derived shifts and experimental values is substantially improved relative to MF-derived shifts. Additionally, the HMC method identifies, for the first time, significant contributions from NNN Mn ions to the ^17^O Fermi contact shift, on the order of several hundred ppm at room temperature (Figure 8f).

Overall, the HMC approach yields more accurate shift predictions for Li_4*e*_ and for the three O sites, and similar predictions to the mean-field method for Li_8*f*_ and Li_4*d*_. Additionally, this approach more faithfully captures symmetrically distinct spin density transfer pathways.^28,31^ Nevertheless, while an explicit treatment of local magnetic environments using ab initio statistical mechanics is an important step toward more accurate paramagnetic shift calculations, as it captures the underlying physics more faithfully than a mean-field approximation, the present results do not provide sufficient evidence to attribute the improved agreement with experiment solely to the description of local magnetic configurations. In particular, it remains unclear whether magnetic environments associated with the same crystallographic site exhibit distinct relaxation behavior, and, if so, whether these differences should be incorporated into the ensemble averaging. Determining nuclear relaxation properties in the presence of both paramagnetic and quadrupolar interactions, as in the case of ^17^O, is nontrivial and lies beyond the scope of this work. Interestingly, the present ensemble averaging scheme yields excellent agreement with experiment despite neglecting differences in relaxation behavior. One possible explanation is that magnetic environments at opposite ends of the distribution (represented by our histogram of *S*_*z*_ values in the Monte Carlo simulation cell, Figure 6) are related by symmetry and may therefore relax on similar timescales.

While the HMC approach yields improved agreement with experiment relative to the mean-field approximation, residual quantitative discrepancies remain, which are significant in the case of ^17^O. One possible source of the remaining error is the neglect of quadrupolar-induced shifts. However, calculations of the electric field gradient tensor for ferromagnetic N-Li_2_MnO_3_ using the Hyb20 functional predict a quadrupolar-induced shift of approximately 32 ppm for ^17^O at 11.7 T. Including this contribution would increase the overall shift and slightly *worsen* the agreement with experiment. In contrast, quadrupolar-induced shifts for ^7^Li are expected to be negligible because of its small quadrupole moment.

Another potential source of error arises from the choice of exchange–correlation functional and basis set. While ^7^Li Fermi contact shifts along a given bond pathway typically vary by 10–50 ppm depending on the degree of Hartree–Fock exchange in the hybrid functional,^28,31,93,98^ ^17^O hyperfine shifts are considerably more sensitive, with variations that can reach several hundred ppm.^93,98^ Optimizing the choice of functional and basis set therefore represents an important avenue for improving quantitative agreement with experiment.

Additional discrepancies may also arise from the neglect of anisotropic hyperfine and spin–orbit coupling effects. Extending the present framework to include cluster expansions of noncollinear hyperfine interactions together with an explicit treatment of the full hyperfine tensor would enable anisotropic contributions to be incorporated directly, while also allowing simulation of the broad spinning sideband manifolds commonly observed in paramagnetic NMR spectra.^13,49^

A limitation of the present approach is its reliance on high-quality first-principles calculations for a representative set of microstates. For chemically complex systems with large unit cells, strong spin–orbit coupling, noncollinear magnetism, or significant dynamical disorder, modeling a sufficiently diverse data set becomes prohibitively expensive. In such cases, active-learning strategies (informed by experimental data) or sparse regression techniques, could be employed to choose which configurations will best sample the configurational space and to build simpler models. We therefore view the present framework as a physically interpretable baseline that can readily incorporate more sophisticated surrogate models as larger and more diverse training data sets become available.

Our analysis of vibrational perturbations provides additional insight into the microscopic origin of finite-temperature Fermi contact shifts. The present study considers only selected symmetry-adapted distortions to illustrate the sensitivity of the Fermi contact shift to lattice dynamics. A fully quantitative treatment would require sampling the complete phonon spectrum (including mode frequencies, eigenvectors, and anharmonic effects) over distinct magnetic orderings. The thermally averaged *S*_*z*_ due to vibrations can be determined, and can then be cluster expanded with respect to magnetic degrees of freedom. This is beyond the scope of this work, but represents an interesting future avenue of work.

Nevertheless, our results demonstrate that vibrational perturbations can modify the instantaneous *S*_*z*_ in a mode-dependent manner, yielding a linear-like variation in *S*_*z*_ with normal mode amplitude in some cases and quadratic-like variations in others. We suggest that the dependence of *S*_*z*_ on normal-mode amplitude is governed by the symmetry of the vibrational mode with respect to the local Li site. In particular, symmetry may either permit or forbid coupling between the normal coordinate and *S*_*z*_ leading to approximately linear or quadratic variations, respectively, of *S*_*z*_ with mode amplitude. Whether the variation of *S*_*z*_ with normal mode amplitude is linear or parabolic will depend on how the vibrational displacement field of the normal mode transforms under the local point group symmetry. Determining the precise correspondence between local irreducible representations and the functional form of the coupling requires a detailed group-theoretical analysis and is left for future work.

The variations in *S*_*z*_ arising from the vibrational modes considered here are at least 1 order of magnitude smaller than those induced by magnetic fluctuations. Furthermore, Li_2_MnO_3_ is a well-ordered crystalline oxide in which significant chemical rearrangement is not expected at typical NMR experimental temperatures. Hence, fluctuations associated with local chemical disorder or compositional dynamics are expected to occur on much longer timescales than vibrational or magnetic fluctuations. This suggests a hierarchy of contributions to the Fermi contact shift in Li_2_MnO_3_, with magnetic fluctuations producing a larger shift than vibrational excitations. A complete finite-temperature description, however, will require consideration of the characteristic timescales of all relevant degrees of freedom relative to the NMR experiment, as fluctuations may contribute either to the averaged shift or to line broadening and relaxation depending on their correlation times.

The present cluster expansion framework provides a natural foundation for extending the model to include additional degrees of freedom. In particular, incorporating chemical disorder and atomic displacements (vibrational degrees of freedom) directly into the effective Hamiltonian, together with cross-terms coupling these variables to magnetic fluctuations, represents a promising direction for future work.

One possible approach to developing a coupled magnetic–vibrational cluster expansion is to first thermally average the hyperfine interactions over vibrational configurations and then determine effective magnetic ECIs from these vibrationally averaged interactions. Alternatively, magnetic and vibrational degrees of freedom could be treated simultaneously through a multidimensional cluster expansion containing explicit cross-terms. More generally, the same framework could be extended beyond phonons to incorporate other local structural degrees of freedom, including defect configurations. We anticipate that materials exhibiting highly frustrated magnetic states, soft phonon modes, and/or strong local structural distortions will show substantially larger deviations between NMR shifts predicted from static electronic structure calculations and experiment, highlighting the importance of explicitly accounting for finite-temperature fluctuations in such systems. Incorporating site disorder into the cluster expansion will be particularly important for materials in which local structural and compositional disorder modifies magnetic coupling pathways. LiNiO_2_ provides one such example, where clustered antisite defects^53,99^ and/or twin boundary defects^100^ have been proposed to induce large, highly localized perturbations of the ^7^Li Fermi contact shift. However, conventional mean-field calculations predict overlapping spectral signatures for these distinct defect environments, preventing unambiguous discrimination between the competing structural models. Similar challenges arise in Li_2_MnO_3_, where assigning ^7^Li resonances associated with planar defects and antisite disorder remains difficult despite support from mean-field DFT calculations.^76,85,101^ By explicitly accounting for magnetic fluctuations in large, structurally disordered models, the present framework provides a pathway toward resolving these ambiguities and establishing more robust correlations between local structure, NMR spectra, and electrochemical behavior.

## Conclusion

The assignment of NMR shifts in paramagnetic solids remains a longstanding challenge. Although conventional bond pathway decomposition methods can aid interpretation, they rely on static, local descriptors and a mean-field treatment of transition metal (TM) magnetization, thereby neglecting magnetic fluctuations and the full distribution of local magnetic environments. A more quantitative and transferable framework is therefore needed to establish a direct connection between local structure, finite-temperature magnetic disorder, and experimentally observed Fermi contact shifts.

In this work, we have shown that the Fermi contact shift is governed by three degrees of freedom: chemical site occupancy, magnetic configuration, and atomic displacements. Using Li_2_MnO_3_ as a model system, we systematically assessed the influence of each contribution on the ^7^Li Fermi contact shift. In particular, we demonstrated that the magnetic configuration of the surrounding TM cations strongly impacts the electron spin density at the Li nucleus and, equivalently, the local value of the *z*-component of the electron spin, *S*_*z*_. Because the Fermi contact shift is proportional to the thermal average ⟨*S*_*z*_⟩, these results demonstrate that magnetic fluctuations must be treated explicitly when evaluating this quantity.

We have developed a cluster expansion framework for predicting *S*_*z*_ that captures local magnetic interactions beyond conventional bond-pathway models. Combined with Heisenberg Monte Carlo simulations, this approach enables accurate, site-resolved predictions of finite-temperature ^7^Li Fermi contact shifts entirely from first principles.

We extended the framework to predict ^17^O Fermi contact shifts, further demonstrating its transferability to other nuclear species. For ^17^O, the method captures shift contributions from second-shell neighbors for the first time, revealing longer-range spin density transfer pathways that have been neglected in previous bond pathway models.

For both ^7^Li and ^17^O, the room-temperature shifts predicted by the cluster expansion Monte Carlo framework show improved agreement with experiment relative to the mean field approximation. We attribute this improvement, at least in part, to the explicit treatment of local magnetic configurations and their thermal fluctuations, although additional developments will be required to achieve fully quantitative agreement with experiment. In particular, future work could incorporate relaxation effects directly into the ensemble-averaging scheme.

Furthermore, although the present framework lays the groundwork for assigning Fermi contact shifts in complex, chemically disordered, and multicomponent solids without requiring a prohibitively large number of ab initio calculations, it also provides a flexible foundation for future developments, including the incorporation of quadrupolar interactions, spin–orbit coupling, noncollinear magnetism, and additional structural degrees of freedom such as atomic displacements and site occupancy.

## Supplementary Material



## Notes

The Supporting Information contains a detailed experimental, notes about applying the cluster expansion to tensor quantities, a Rietveld refinement to powder neutron diffraction data, additional experimental ^7^Li NMR spectra, bulk magnetic susceptibility measurements, Monte Carlo simulated susceptibility data, additional cluster expansion fits for ^17^O and structural information about the models used in this work (PDF)

## ∥

Centre de RMN à Très Hauts Champs de Lyon (UMR 5082—CNRS, ENS Lyon, UCB Lyon 1), Université de Lyon, 69100 Villeurbanne, France
