# Supplementary material for: A Statistical Mechanical Framework for Predicting Fermi Contact NMR Shifts
Source: J Am Chem Soc. 2026 Aug 14;148(33):35575–93. doi: 10.1021/jacs.6c07045 (PMC13523746; doi:10.1021/jacs.6c07045)
Supplement: Supplementary file 1 [file ja6c07045_si_001.pdf]

Supporting Information for: A Statistical Mechanical Framework for Predicting Fermi Contact NMR Shifts

Euan N. Bassey,[†,‡,¶] Elias Sebti,[†,‡] Anton Van der Ven,[*,†] and Raphaële J. Clément[*,†,‡,§]

†*Materials Department, University of California Santa Barbara*

‡*Materials Research Laboratory, University of California Santa Barbara*

¶*Present address: Centre de RMN à Très Hauts Champs de Lyon (UMR 5082 – CNRS, ENS Lyon, UCB Lyon 1), Université de Lyon, 69100 Villeurbanne, France*

§*Max Planck Institute for Solid State Research, Stuttgart, 70569, Germany*

E-mail: avdv@ucsb.edu; rclement@ucsb.edu

Contents

1 **Experimental** 3

2 **Tensorial Cluster Expansion of $\langle\vec{S}\rangle$** 7

3 **Neutron Diffraction Rietveld Refinement** 9

4 **Experimental ^{7}Li NMR Spectra** 10

5 **Bulk magnetic susceptibility measurements** 11

6 **Additional plots on the variation of S_z with $S_{\mathrm{tot.}}$** 12

7 **Additional Cluster Expansion Fits** 13

8 **Prediction of Finite-Temperature NMR Spectrum using Heisenberg Monte Carlo Simulations** 15

9 **Comparison of Monte Carlo, Mean Field and Experimental Shifts** 20

10 **Additional ^{17}O NMR Fermi contact shift calculation results** 22

11 **Structural Models of Li_2MnO_3 Used in This Study** 26

12 **Effect of Normal Modes on the Fermi Contact Shifts for ^{7}Li and ^{17}O** 30

13 **Effect of Harmonic Lattice Vibrations on Room-Temperature Fermi Contact Shifts** 31

14 **Effect of Normal Mode Displacements on Local Structure around Li and O** 42

1 Experimental

Synthesis. Powder samples of Li_2MnO_3 were prepared *via* a high-temperature solid-state synthesis. Stoichiometric quantities of Li_2CO_3 and Mn_2O_3 were mixed, then calcined at 650 °C (5 °C min^{-1}) for 12 hr in air in an alumina crucible. The powder was ground in an agate pestle and mortar, pelletized and fired at 850 °C (5 °C min^{-1}) for 24 hr in air.

Powder Neutron Diffraction. Powder neutron diffraction measurements were carried out at the Institut Laue-Langevin using the D2B instrument.[1] A powder sample of Li_2MnO_3 was loaded into a thin-walled vanadium canister (5 mm diameter). The background was fit using a 12-term Chebyshev polynomial. The lattice parameters were refined alongside the site occupancies for Li and Mn. Rietveld[2,3] refinements were carried out using the TOPAS Academic 7.0 structure refinement software package.[4,5]

^{7}Li Nuclear Magnetic Resonance Spectroscopy. Samples of Li_2MnO_3 were packed into 1.3 mm diameter ZrO_2 magic angle spinning (MAS) rotors in an Ar-filled glovebox. Each rotor spent no longer than 10 minutes outside of the glovebox before being inserted into the magnet under a protective atmosphere of flushing nitrogen gas. NMR spectra were referenced to 1.0 M aqueous LiCl (0 ppm). ^{7}Li NMR spectra were recorded on a Bruker AVANCE spectrometer (2.35 T) using a home-built 1.3 mm MAS probe, an MAS frequency of 60 kHz, and a $\pi/2$ pulse length of 1.75 μs.

Rotor-synchronized Hahn-echo pulse sequences ($\pi/2-\tau-\pi-\tau-$acquire) were used throughout. The recycle delay (100 ms) was set such that the bulk, paramagnetically shifted signal was recorded quantitatively, while the diamagnetic signal (due to small quantities of amorphous impurities at the surface of particles) was suppressed. An additional spectrum, using a longer recycle delay of d_1 = 10 s, Projection magic angle turning phase-adjusted sideband suppression (pj-MATPASS) experiments were also recorded to separate the isotropic resonances from the overlapping spinning sideband manifold.[6] Relaxation times—spin-lattice, T_1, and spin-spin, T_2—were recorded using saturation-recovery and pseudo-two-dimensional Hahn-echo experiments.

Magnetic Susceptibility Measurements. Magnetic susceptibility measurements were carried out on a powder sample of Li_2MnO_3 (27.1(2) mg) using a Quantum Design Magnetic Property Measurement System 3 (MPMS3) superconducting quantum interference device (SQUID) magnetometer. The zero-field cooled (ZFC) and field-cooled (FC) susceptibilities were measured in a field of 0.1 T over a temperature range 2-300 K. As $M(H)$ is linear in this field regime,, the small-field approximation to the susceptibility, $\chi \approx M/H$, was assumed to be valid. The data were corrected for diamagnetism of the sample using Pascal's constants.[7]

First-Principles Calculations of Fermi Contact Shifts. To begin with, a $(2 \times 2 \times 1)$ supercell of the Li_2MnO_3 (space group $C2/m$) experimental structure determined from Rietveld refinements of the neutron diffraction data was constructed ($a \simeq 4.96$Å, $b \simeq 8.58$Å, $c \simeq 9.65$Å, $\alpha = 90°$, $\alpha \simeq 99.5°$, $\gamma = 90°$, composition $Li_{16}Mn_8O_{32}$). This experimental structure was relaxed with either all electronic spins on Mn aligned ferromagnetically or antiferromagnetically. In the antiferromagnetic case, nearest-neighbor Mn spins were set anti-parallel to one another. Structural relaxations were performed using the VASP code,[8–10] employing the projector-augmented wave method.[11,12] These calculations employed the SCAN *meta*-GGA exchange-correlation functional,[13–15] and a plane-wave cutoff energy set to 520 eV. The Brillouin zone was sampled with a Monkhorst-Pack[16] *k*-point mesh with a mesh density of < 0.05 $Å^{-1}$. All electronic structures were converged to 10^{-5} eV for energies, while forces on atoms were converged to 0.02 eV $Å^{-1}$. The PAW pseudopotentials used in the DFT calculations were: Li_sv (valency of 3) for lithium, Mn_pv (valency of 13) for manganese, Ni (valency of 10) for nickel, and O (valency of 6) for oxygen. Gaussian smearing with a width of 0.05 eV was used for all calculations. These relaxed cells were then used to enumerate 44 collinear (Ising-like) spin configurations, generated using the CASM software package.[17] Calculations of the formation energies of these configurations was conducted using both VASP and CRYSTAL23.[18–20] The parameters used for CRYSTAL23 calculations are detailed below.

^{7}Li Fermi contact shifts were calculated using methods described previously.[21–24] Periodic spin-polarized hybrid density functional theory (DFT) - Hartree Fock (HF) calculations were

performed using CRYSTAL23. The structures considered in this work were the original experimental ($2 \times 2 \times 1$) supercell, as well as the ferromagnetically and antiferromagnetically relaxed structures. The spin configuration was initially locked to the configuration generated by CASM and the electronic wavefunction was optimized under fixed cell and structure constraints. This spin-locked wavefunction was then used to initialize a final calculation of the wavefunction without spin configuration restrictions; in all cases, the spin configuration was maintained relative to the original configuration generated by CASM (*i.e.*, no spin flips occurred between the final wavefunction and the initial configuration generated by CASM). The unpaired electron spin densities at the ^{7}Li nuclear positions were calculated using the B3LYP[25,26] hybrid functional (20% HF exchange), referred to as Hyb20. Additional calculations using a modified B3LYP functional with 35% Fock exchange were also run. These calculations employed extended basis sets, taken from the Ahlrichs set for metal ions[27] and the IGLO-III basis set for O.[28] The NMR shift calculations employed two basis sets, taken from the Ahlrichs set for metal ions[27] and the IGLO-III basis set for O.[28] A smaller basis set for geometry optimisations (denoted BS-I) and a more extended set for the single-point hyperfine calculations (BS-II). The BS-I sets were taken without modification—from solid-state studies by Catti et al.,[29–31] whilst the BS-II sets comprised bases from the Ahlrichs set for metal ions[32] and IGLO-III basis set for O.29 The BS-I sets were of the form $(9s2p)/[5s2p]$ for Li, $(20s12p5d)/[1s4sp2d]$ for Mn, and $(14s6p)/[1s3sp]$ for O. The BS-II sets are of the form $(9s2p)/[5s2p]$ for Li, $(13s10p5d)/[7s6p3d]$ for Mn, and $(10s6p2d)/[6s5p2d]$ for O. In both cases, the values in parentheses denote the number of Gaussian primitives and the values in square brackets denote the contraction scheme. Initial single-point calculations were carried out on a ferromagnetic configuration without optimizing the geometry generated by VASP, with an energy tolerance of 10^{-7} AU, a root-mean-square force gradient tolerance of 3×10^{-4} AU and integral thresholds of 10^{-7}, 10^{-7}, 10^{-7}, 10^{-7} and 10^{-14} for the Coulomb overlap, Coulomb penetration, exchange overlap and g and n series exchange penetration, respectively.

Cluster Expansions of the Fermi Contact Shift and of the Total Energy. As described in the Background section, the Fermi contact shifts and total energy of each of the 44 configurations

of interest were first computed from first principles. The resulting shifts and energies were then fit against the effective cluster functions describing the configurations using the method described in the main text (eq. (17)). For this, *ab initio* shifts and energies were first separated into different groups, one for each crystallographically distinct site (shift) or configuration (energy). Then we separated the data for each site or configuration into training and testing sets, the latter comprising 10% of the data points for a single crystallographic environment. To assess the predictive accuracy of the model, a k-fold cross-validation procedure was employed, in which the dataset was partitioned into k subsets; the model was trained on $k-1$ subsets and validated on the remaining subset. The optimal regularization parameter and number of cluster terms were determined by minimizing the cross-validation error.

Monte Carlo Simulations to Obtain Finite-Temperature Fermi Contact Shifts. The ECIs obtained from the energy cluster expansion fit were provided as input to an in-house Monte Carlo code integrated into CASM[33] to determine the magnetic state (site-specific magnetic spins) at the experimentally-relevant temperature. The ^{7}Li NMR Fermi contact shift was determined for each Li site within a large supercell (8×8×8) relative to the primitive cell, giving 2048 Mn atoms) from the product of the cluster functions obtained from Monte Carlo and the corresponding ECIs from the shift cluster expansion. Finally, the shifts of crystallographically distinct Li sites were averaged over the entire cell to obtain an average shift for each site.

2 Tensorial Cluster Expansion of $\langle\vec{S}\rangle$

When the moments on *TM* cations are free to adopt any spatial orientation, as in a Heisenberg model, a tensorial cluster expansion is necessary.[34] Instead of expanding only the z-component of $\langle\vec{S}\rangle$, the full vector must be expanded:

$$\begin{aligned}\langle S_\alpha\rangle = &\sum_l \sum_\gamma \Lambda^l_{\alpha,\gamma} M^l_\gamma + \\ &\sum_{l,m,n} \sum_{\gamma,\epsilon,\tau} \Gamma^{l,m,n}_{\alpha,\gamma,\epsilon,\tau} M^l_\gamma M^m_\epsilon M^n_\tau + \cdots .\end{aligned} \tag{1}$$

In eq. 1, $\langle S_\alpha\rangle$ is the αth component of $\langle\vec{S}\rangle$, $\Lambda^l_{\alpha,\gamma}$ and $\Gamma^{l,m,n}_{\alpha,\gamma,\epsilon,\tau}$ are expansion coefficients, and M^l_γ is the γth component of the magnetic moment vector $\vec{M}^l$ at site l. As with the Ising expansion of $\langle S_z\rangle$, time reversal symmetry preserves only the odd terms in M^l_γ. In contrast to the Ising expansion of $\langle S_z\rangle$, however, the expansion in eq. 1 must be equivariant to the local site point group.

We note that this full tensorial expansion is necessary for solids where spin-orbit coupling is significant. Even in the absence of spin-orbit coupling, a Heisenberg magnetic model is necessary to recover the correct magnetic response at finite temperature.[33,35,36] In the absence of spin-orbit coupling, however, eq. 1 must be equivariant to the symmetry of free space, providing further restrictions on the expansion coefficients. For example, if only point terms are considered, the expansion in the absence of spin-orbit coupling becomes:

$$\langle\vec{S}\rangle = \sum_l J_l \vec{M}^l, \tag{2}$$

where J_l are the same expansion coefficients as in eq. 16 in the main text.

The framework can further be generalized to include lattice displacements and chemical disorder by extending the tensor cluster expansion to depend on magnetic, vibrational, and occupational degrees of freedom.[17]

If all ions in a crystal, l, have a displacement vector, $\vec{u}_l$, measuring the ion positions relative to a high-symmetry reference point then we can collect the displacement vectors of all ions into a quantity $\tilde{\mathbf{u}} = (\vec{u}_1, ..., \vec{u}_N)$. Likewise, the chemical occupancy at each site may be described by a quantity p_l, which, collected over all sites becomes $\tilde{\mathbf{p}} = (p_1, ..., p_N)$. The cluster expansion then becomes:

$$\langle S_\alpha \rangle = \sum_c \Lambda^c_{\alpha,\gamma} \Phi_{c,\gamma} \left(\tilde{\mathbf{M}}, \tilde{\mathbf{u}}, \tilde{\mathbf{p}} \right). \tag{3}$$

Here, the sum extends over all clusters c with symmetrically distinct basis functions $\Phi_{c,\gamma}$, weighted by the ECI $\Lambda^c_{\alpha,\gamma}$.

3 Neutron Diffraction Rietveld Refinement

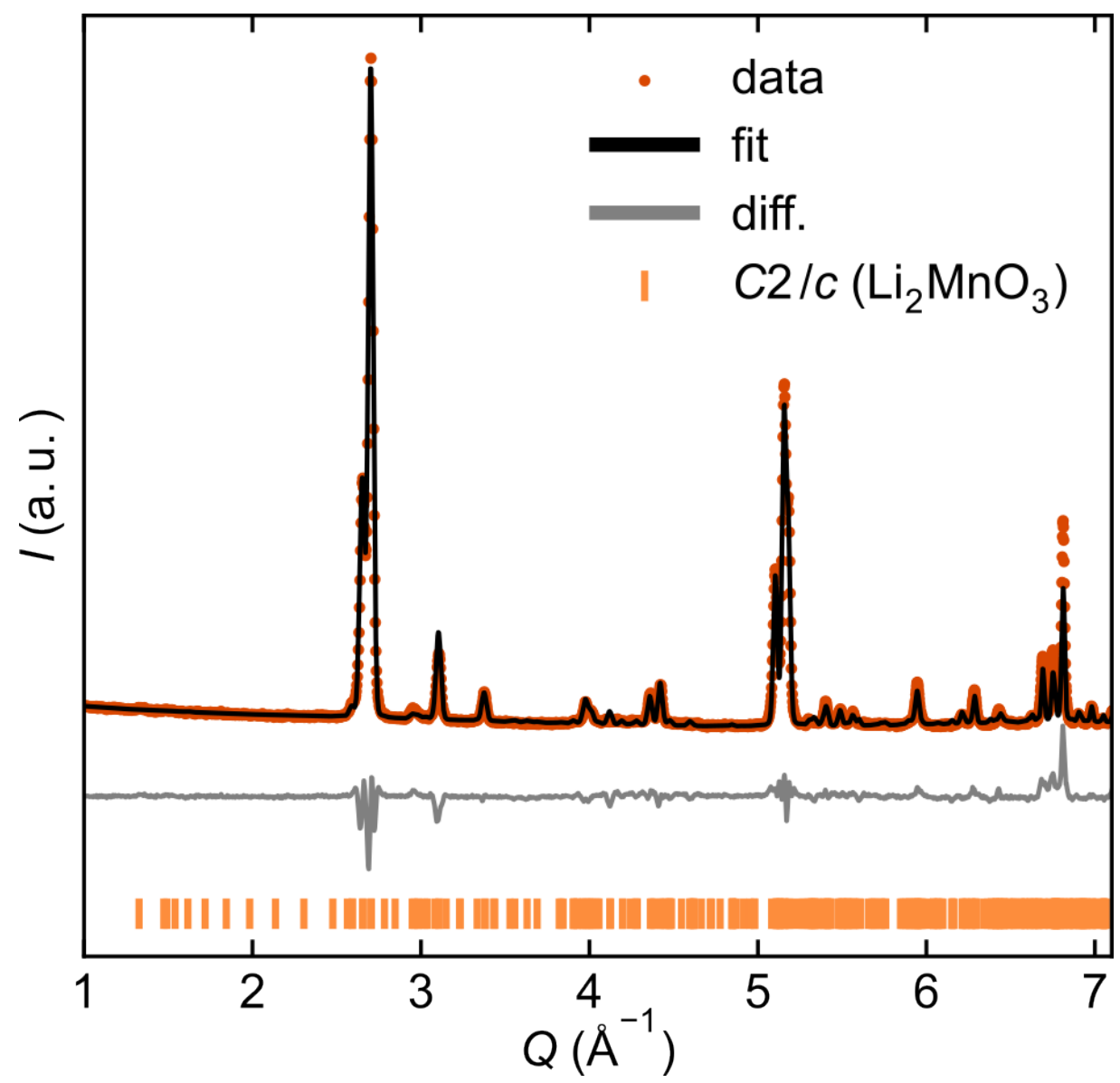

Figure S1: Rietveld refinement of the powder neutron diffraction pattern acquired at room temperature on Li_2MnO_3, $R_{\mathrm{w.p.}} = 9.1\%$.

4 Experimental ^{7}Li NMR Spectra

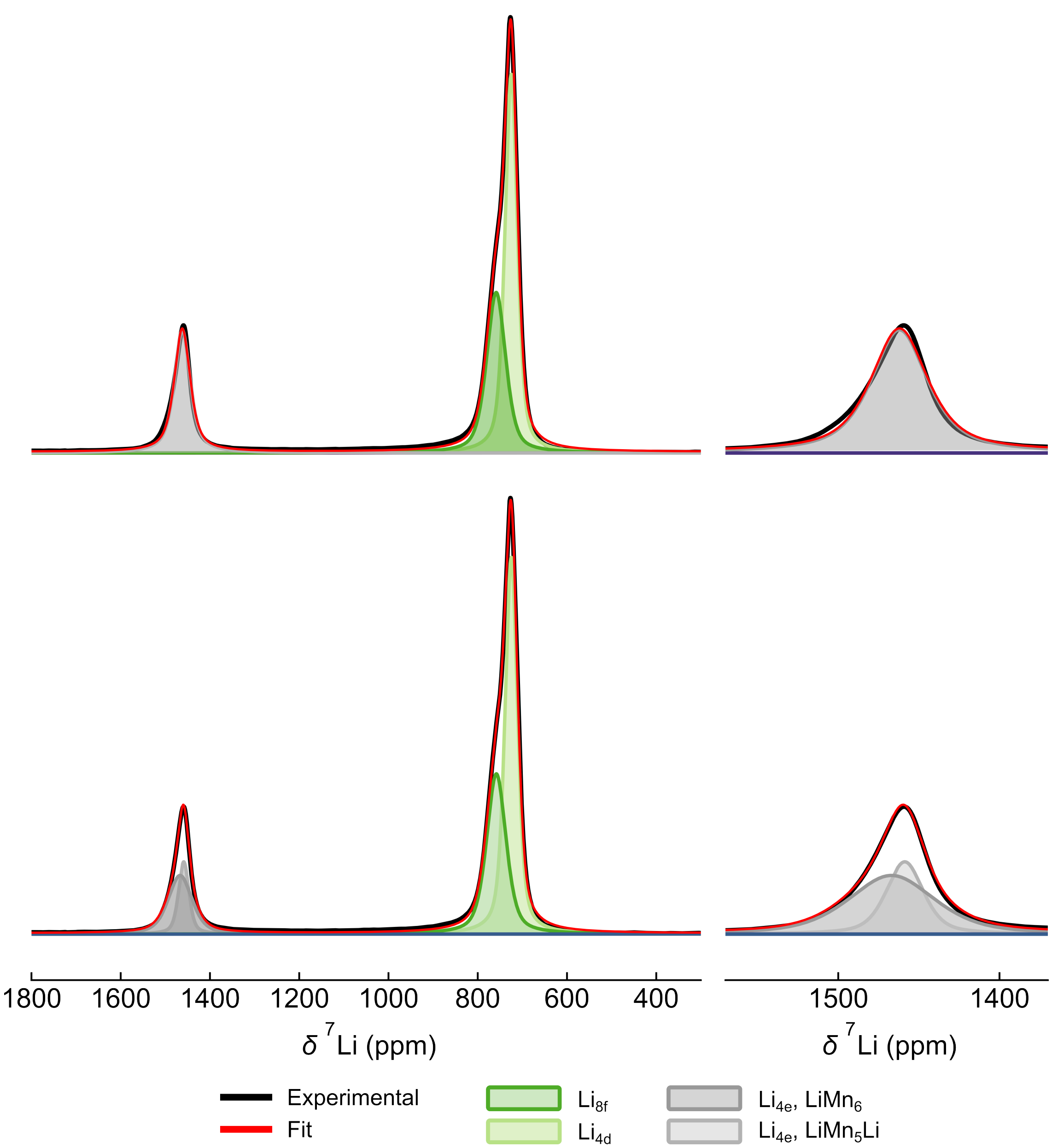

Figure S2: Fits to the experimentally observed ^{7}Li NMR spectrum (2.35 T, 60 kHz MAS) using: a three-component model (top) and a four-component model (bottom). On the right, a zoom-in of the high-frequency region, corresponding to the Li_{4e} environment, is shown with the same fits.

5 Bulk magnetic susceptibility measurements

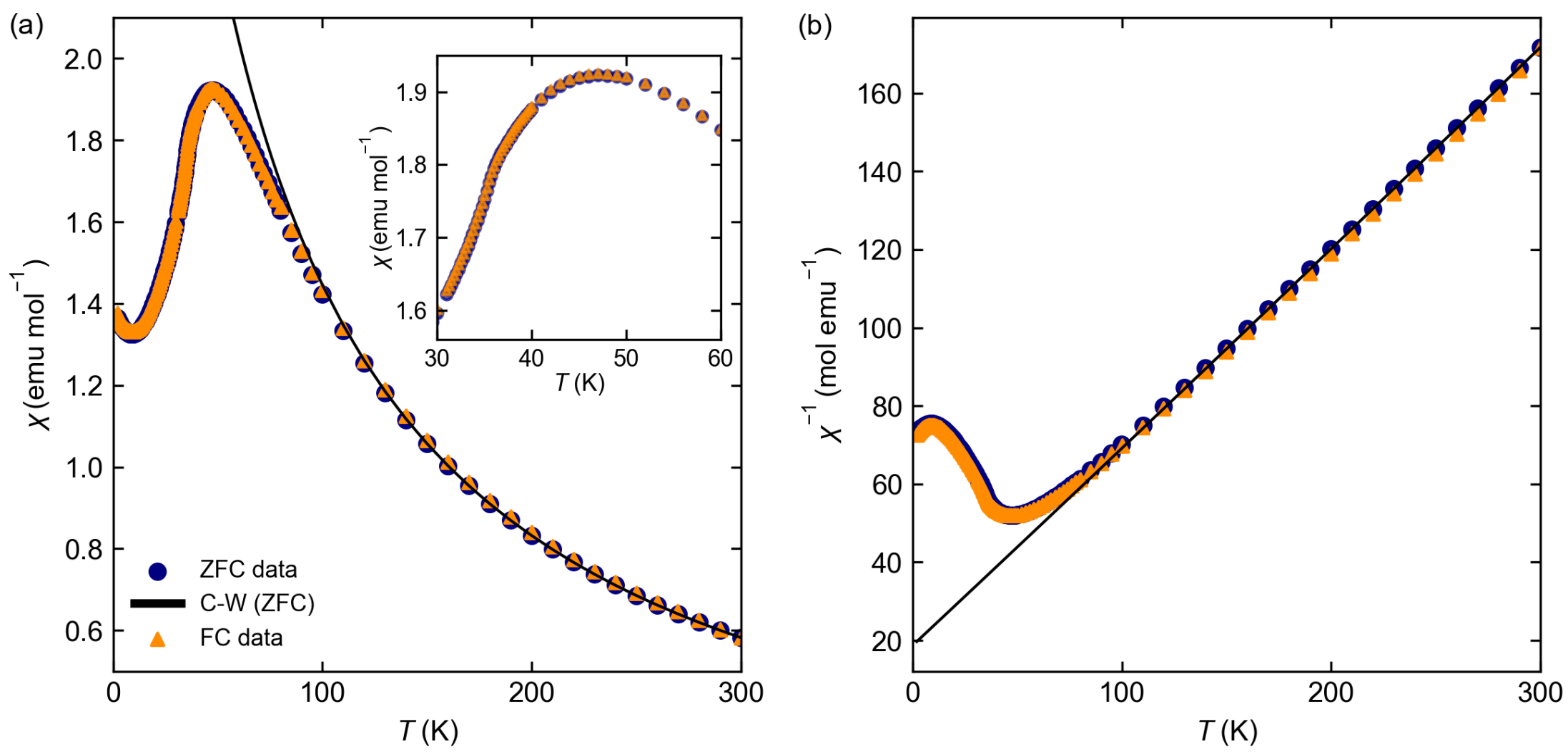

Figure S3: Susceptibility data for powder Li_2MnO_3, recorded at a field of 0.1 T: in **(a)**, plotted as χ *vs* T, and in **(b)** as χ^{-1} *vs* T. A Curie-Weiss law has been fit to the data above 150 K, with fitted values of $\mu_{\text{eff.}} = 3.87\ \mu_{\text{B}}\ \text{mol}^{-1}_{\text{Mn}}$ and Weiss constant $\theta = -45$ K.

6 Additional plots on the variation of S_z with $S_{\text{tot.}}$

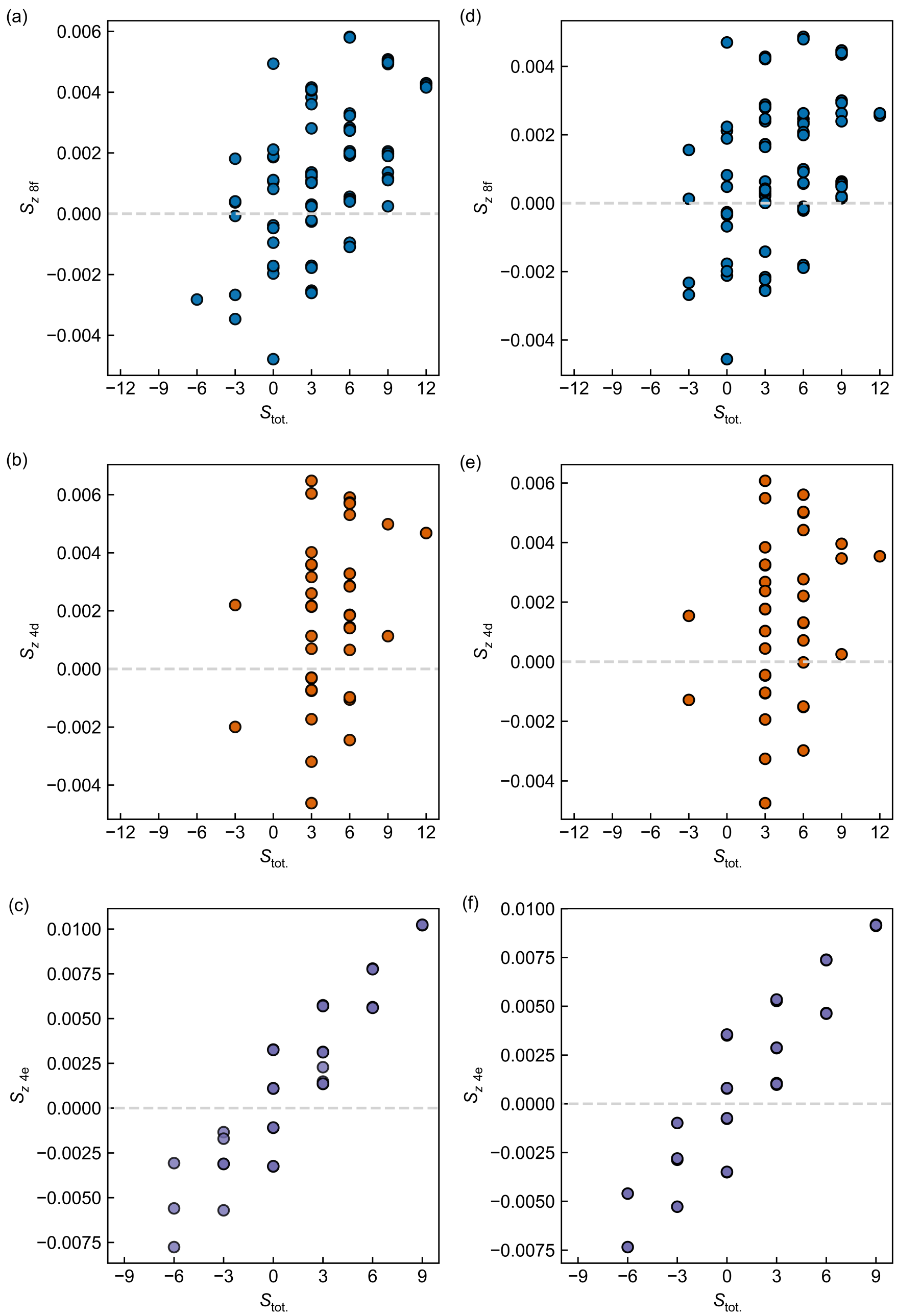

Figure S4: z-component of the local electron spin, S_z, for various magnetic environments of **(a), (d)** Li_{8f}, **(b), (e)** Li_{4d}, and **(c), (f)** Li_{4e} in N-Li_2MnO_3, plotted against the total spin, $S_{\text{tot.}}$ of the Mn cations surrounding the Li sites, up to a maximum radius of 4.2 Å. In **(a)**, **(b)** and **(c)**, S_z values are calculated using the Hyb20 functional. In **(d)**, **(e)** and **(f)**, S_z values are calculated using the Hyb35 functional. The non-linear variation in S_z with $S_{\text{tot.}}$ indicates that a cluster expansion is required.

7 Additional Cluster Expansion Fits

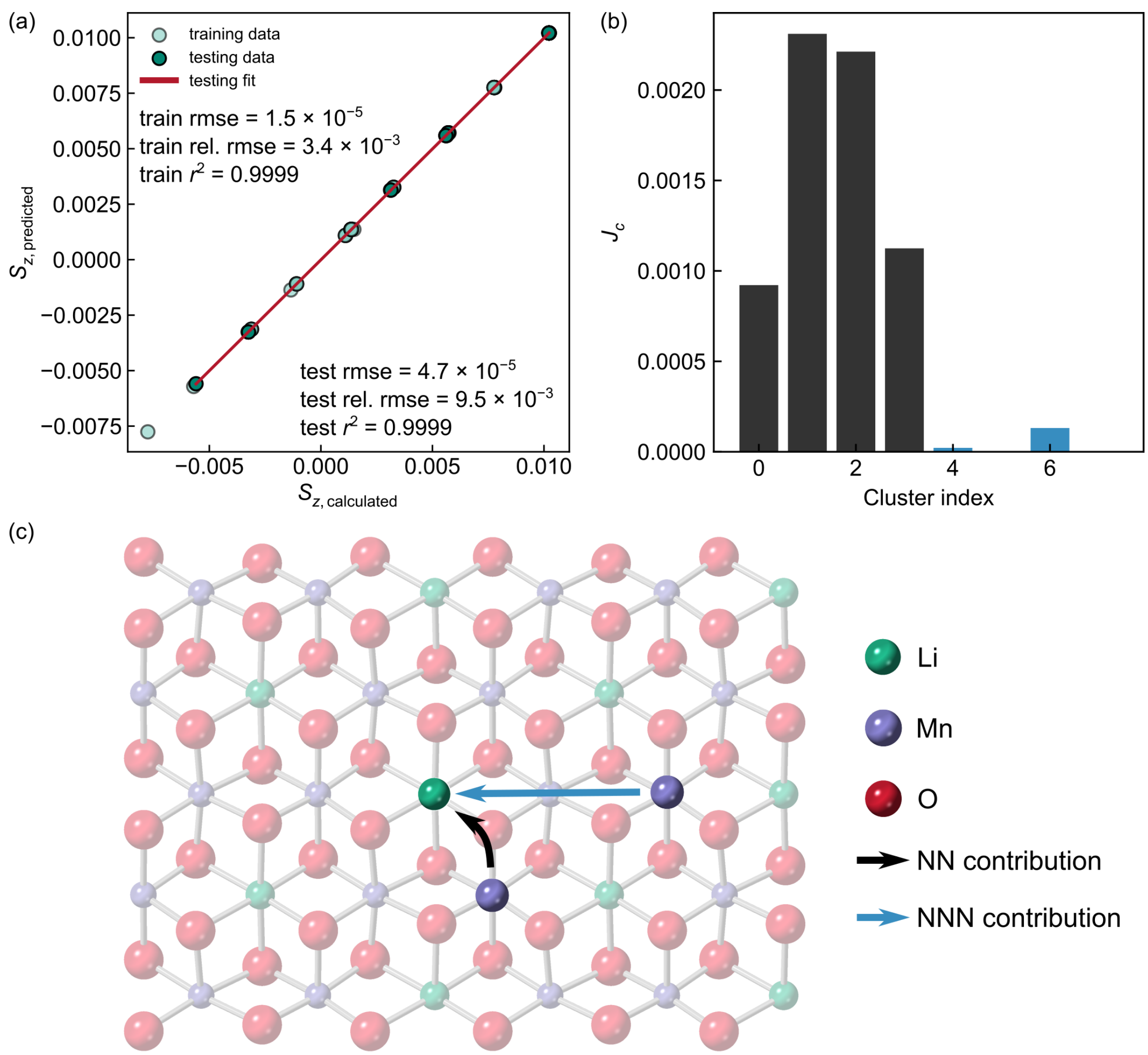

Figure S5: **(a)**: Cluster expansion fit of S_z for Li_{4e}. S_z values were computed using the Hyb20 functional in CRYSTAL. This fit includes nearest and next-nearest neighbor (NN and NNN, respectively) clusters in the effective Hamiltonian, unlike in the main text where only NN were considered. The root-mean square error (rmse) and CV-score (r^2) were computed using *k*-fold cross-validation, and the relative rmse is defined as the rmse normalized by the standard deviation of the calculated S_z. **(b)**: Effective cluster interactions (ECIs) obtained from the fit in (a). **(c)**: Interaction pathways between the Li nucleus and neighboring Mn centers associated with the NN and NNN ECI types.

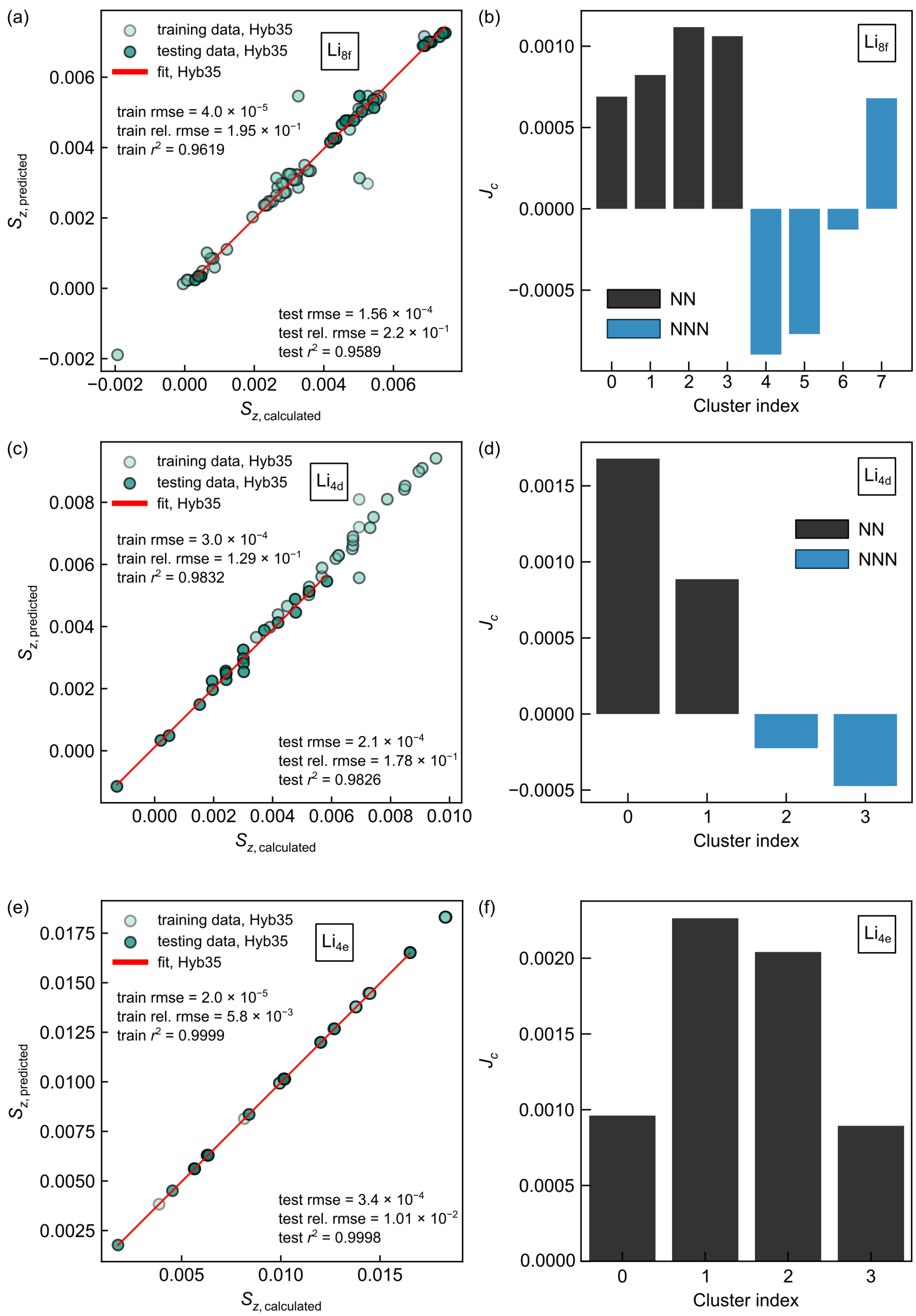

Figure S6: Fits and plots of the ECIs for the cluster expansion of S_z for: **(a)** and **(b)** Li_{8f}, **(c)** and **(d)** Li_{4d}, and **(e)** and **(f)** Li_{4e} sites. S_z values were computed using the Hyb35 functional in CRYSTAL.

8 Prediction of Finite-Temperature NMR Spectrum using Heisenberg Monte Carlo Simulations

To obtain the finite-temperature NMR spectrum of Li_2MnO_3, we multiply our ECI values by the z-component of the electronic spins, the latter determined using a Heisenberg Monte Carlo simulation at an applied magnetic field of 2.35 T, consistent with experiment. Details of the Heisenberg Monte Carlo model used can be found in ref.[33] After enumerating all configurations using the CASM software package,[17] we determined the total energy of each configuration using the Hyb20 functional as implemented in CRYSTAL23.

The effective Hamiltonian was constructed by fitting the total energies to a simple collinear spin model:

$$\mathcal{H} = \sum_{\langle i,j \rangle} J_{i,j} \vec{s}_i \cdot \vec{s}_j, \tag{4}$$

where $J_{i,j}$ is a coefficient that reports the strength and nature of the exchange interaction between the ith and j^{th} sites. The fits to the Hamiltonian are shown in Figure S7, whilst the Monte Carlo results obtained (using the exchange constants obtained in Figure S7(b)) are shown in Figure S9.

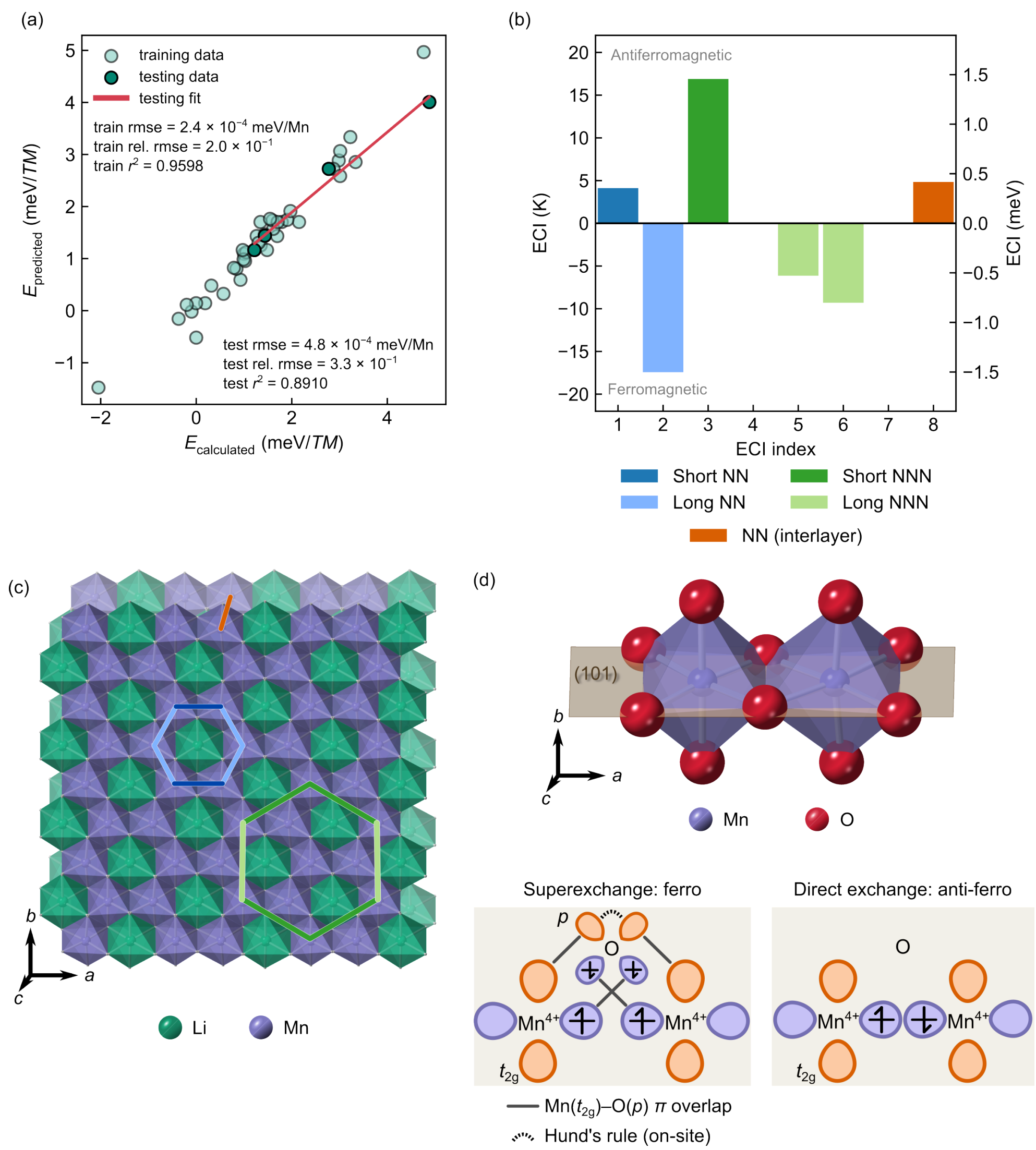

Figure S7: Magnetic energy cluster expansion results for N-Li_2MnO_3, with energies computed using the Hyb20 functional. In **(a)**, the cluster expansion fit is shown, with the corresponding ECI plotted in **(b)**. These ECI correspond to the interactions highlighted as lines connecting pairs of Mn ions in the structure shown in **(c)**. These lines are shown in the same colors as the bars in **(b)**. In **(d)**, the exchange interaction for a pair of Mn ions is shown, highlighting the direct competition between ferromagnetic superexchange and anti-ferromagnetic direct exchange.

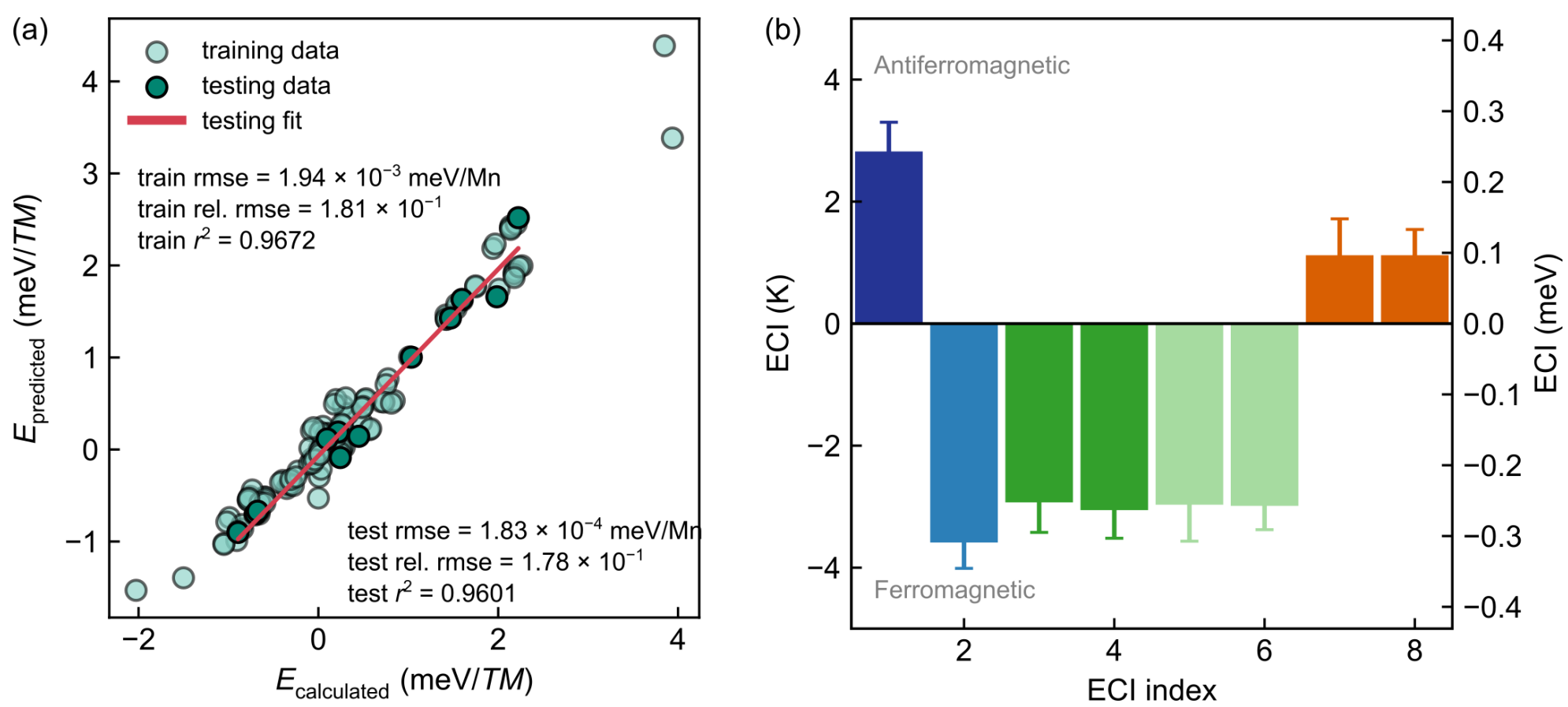

Figure S8: Magnetic energy cluster expansion results for N-Li_2MnO_3, with energies computed using the Hyb35 functional. **(a)** Cluster expansion fit, and **(b)** corresponding ECIs.

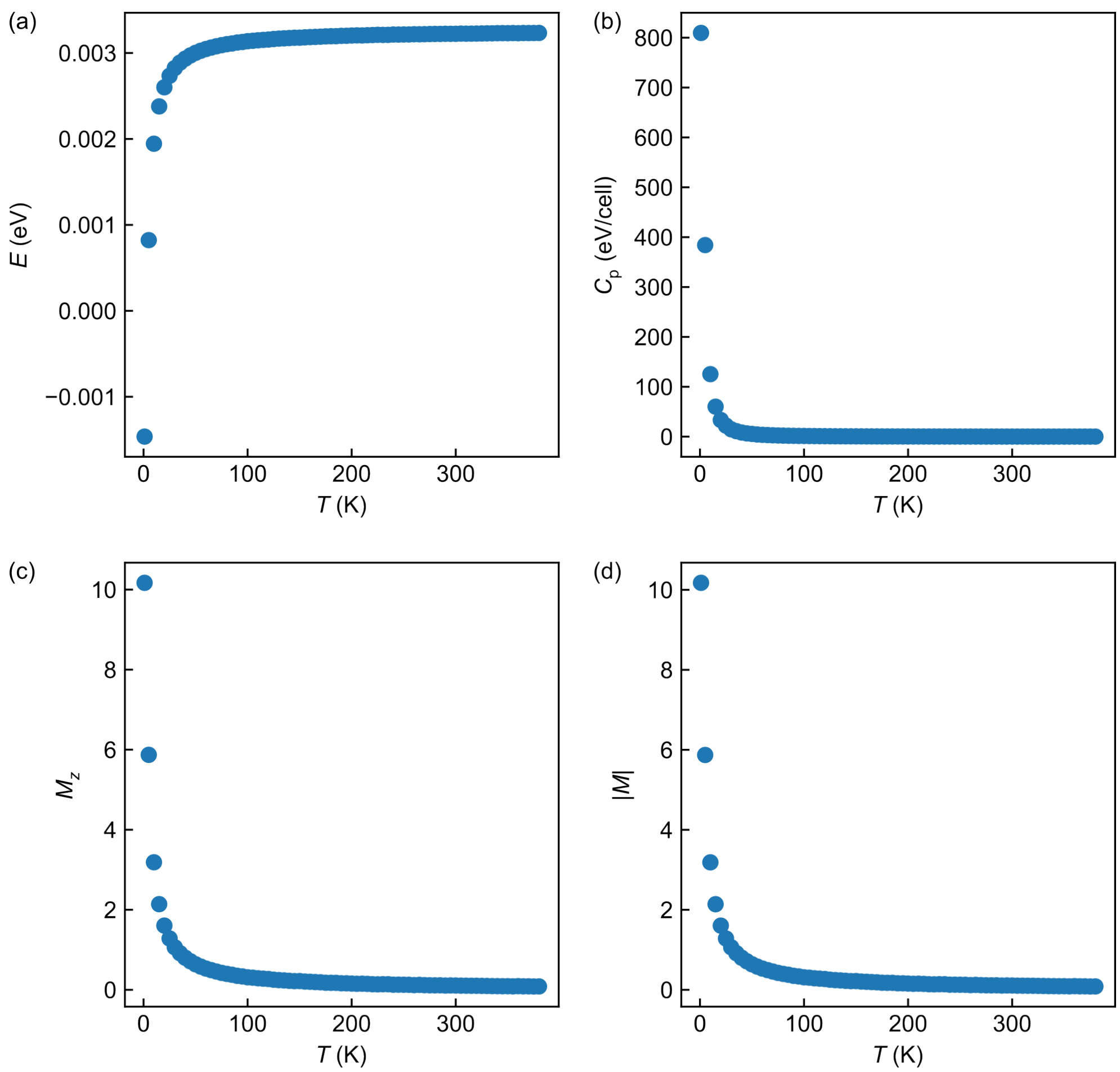

Figure S9: Heisenberg Monte Carlo (HMC) results using the Hyb20 magnetic energy cluster expansion on N-Li_2MnO_3, showing the temperature variation in **(a)** the energy, **(b)** the heat capacity, **(c)** the z-component of the sum of all magnetic moments on Mn, and **(d)** the magnitude of the total magnetic moment vector (summed over all Mn). This HMC was performed on a cell containing 2048 Mn ions.

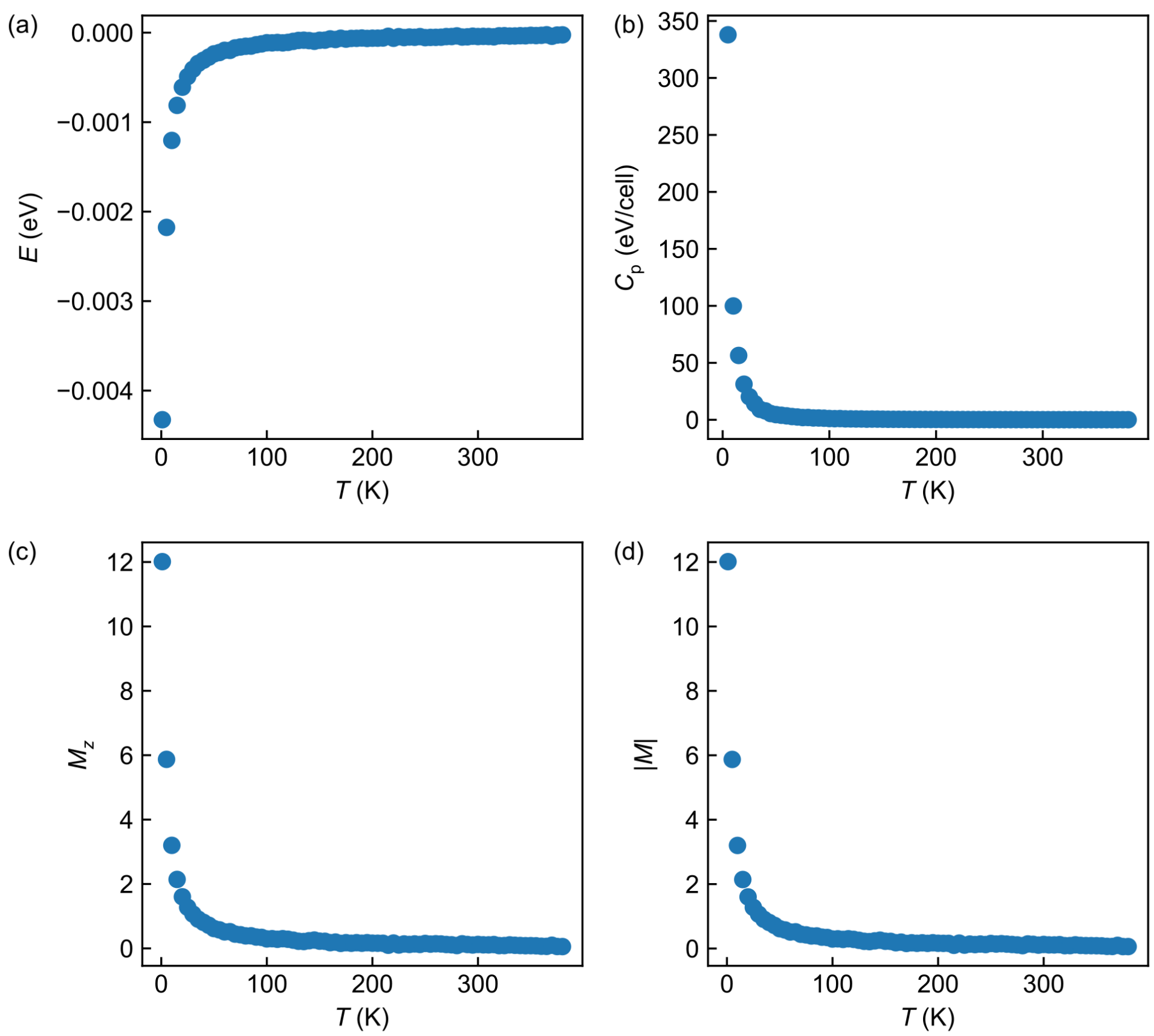

Figure S10: Heisenberg Monte Carlo (HMC) results using the Hyb35 magnetic energy cluster expansion on N-Li_2MnO_3, showing the temperature variation in **(a)** the energy, **(b)** the heat capacity, **(c)** the z-component of the sum of all magnetic moments on Mn, and **(d)** the magnitude of the total magnetic moment vector (summed over all Mn). This HMC was performed on a cell containing 2048 Mn ions.

9 Comparison of Monte Carlo, Mean Field and Experimental Shifts

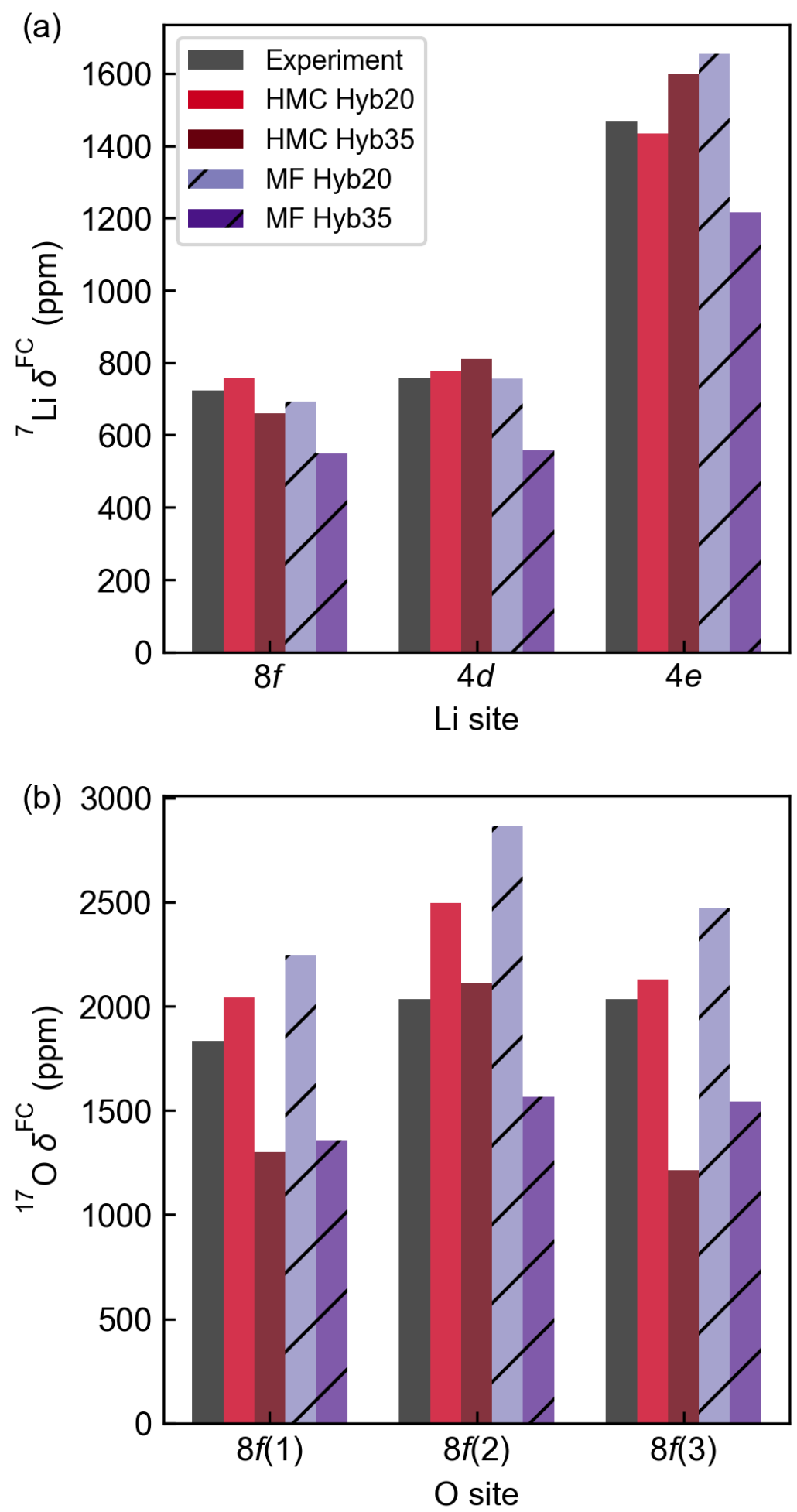

Figure S11: Comparison of experimental and calculated Fermi contact shifts, δ^{FC}, for **(a)** ^{7}Li and **(b)** ^{17}O in Li_2MnO_3. Experimental shifts were obtained at 60 kHz MAS (320 K) and at a field of 2.35 T (^{7}Li) and 11.7 T (^{17}O). Heisenberg Monte Carlo (HMC) and mean field (MF) shifts were obtained from first principles calculations on N-Li_2MnO_3 using the Hyb20 and Hyb35 functionals. HMC shifts were determined by combining ECIs from a magnetic cluster-expansion with Monte Carlo-derived Heisenberg Mn moments at 320 K and at the field of interest. MF shifts were obtained by scaling the first principles shifts from 0 K to 320 K according to eq. 15 ($\theta = -45$ K, $\mu_{\mathrm{eff}} = 3.87\ \mu_{\mathrm{B}}\ \mathrm{mol_{Mn}}^{-1}$).

Table S1: Experimental and calculated ^{7}Li and ^{17}O Fermi contact shifts, δ^{FC}, in N-Li_2MnO_3 using hybrid functionals with 20% and 35% Hartree–Fock exchange (Hyb20 and Hyb35). Heisenberg Monte Carlo (HMC) shifts were obtained by combining cluster-expanded magnetic ECIs with Monte Carlo-derived Mn moments. Mean field (MF) shifts were scaled to room temperature using the Curie–Weiss scaling in eq. 15 ($\theta = -45$ K, $\mu_{\mathrm{eff}} = 3.87\ \mu_{\mathrm{B}}\ \mathrm{mol_{Mn}}^{-1}$). Experimental values for ^{7}Li were measured at 320 K (MAS 60 kHz) at a field of 2.35 T; ^{17}O values were measured at 320 K (MAS 60 kHz) at a field of 11.7 T.

	^{7}Li: δ^{FC} (ppm)			Deviation from experiment			Site-to-site differences (ppm)	
Spectrum	8f	4d	4e	8f	4d	4e	$4d - 8f$	$4e - \frac{8f+4d}{2}$
Exp.	724	757	1466	0	0	0	+33	+726
HMC (Hyb20)	758	777	1433	+5%	+3%	−2%	+19	+666
MF (Hyb20)	693	756	1653	−4%	−0.1%	+13%	+63	+929
HMC (Hyb35)	660	811	1599	−9%	+7%	+9%	+151	+864
MF (Hyb35)	548	557	1216	−24%	−26%	−17%	+9	+664
	^{17}O: δ^{FC} (ppm)			Deviation from experiment			Site-to-site differences (ppm)	
Spectrum	8f(1)	8f(2)	8f(3)	8f(1)	8f(2)	8f(3)	$8f(2) - 8f(1)$	$8f(1) - \frac{8f(2)+8f(3)}{2}$
Exp.	1833	2033	2033	0	0	0	+200	−200
HMC (Hyb20)	2043	2496	2129	+11%	+23%	+5%	+453	−270
MF (Hyb20)	2245	2868	2468	+22%	+41%	+21%	+623	−423
HMC (Hyb35)	1302	2109	1215	−29%	+4%	−40%	+807	−360
MF (Hyb35)	1357	1566	1542	−26%	−23%	−24%	+209	−197

10 Additional ^{17}O NMR Fermi contact shift calculation results

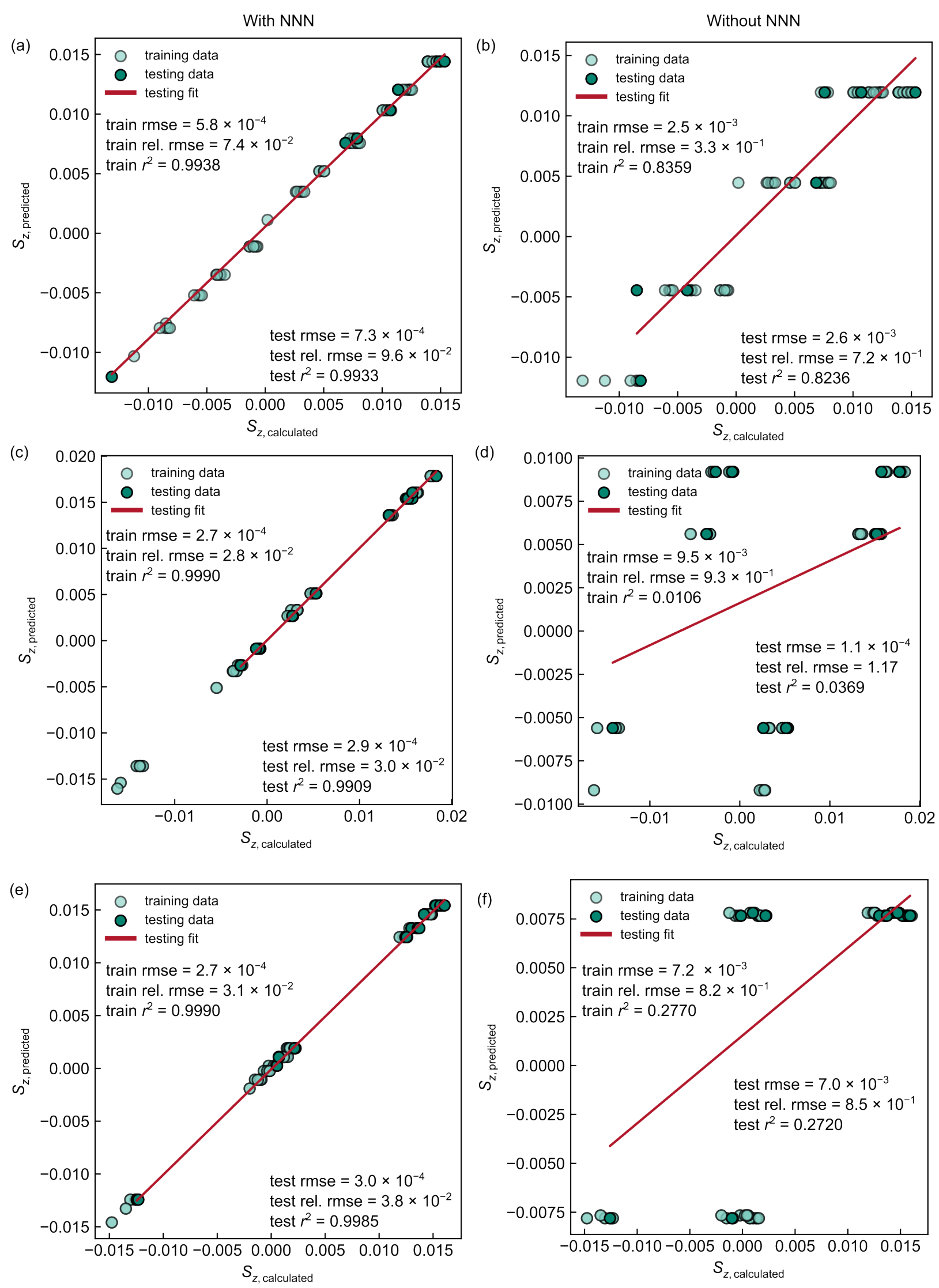

Figure S12: Cluster expansion fits for the Hyb20-calculated spin density at ^{17}O nuclear positions for: $O_{8f(1)}$ (**(a)**, **(b)**), $O_{8f(2)}$ (**(c)**, **(d)**), and $O_{8f(3)}$ (**(e)**, **(f)**), with and without next-nearest neighbor terms, respectively. The root-mean square error (rmse) and CV-score (r^2) were computed using k-fold cross-validation.

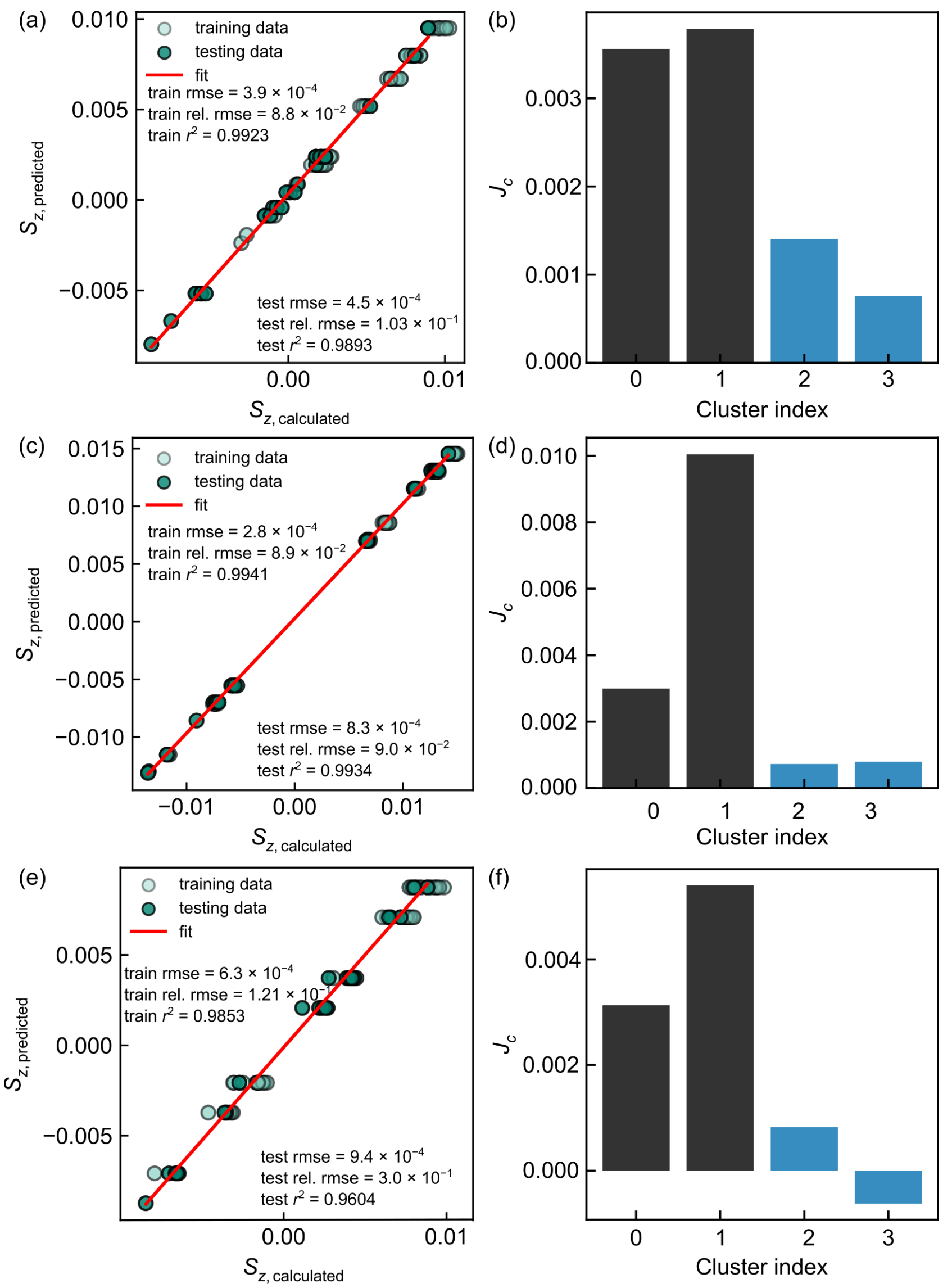

Figure S13: Cluster expansion fits and effective cluster interaction coefficients for the Hyb35-calculated spin density at ^{17}O nuclear positions for: $O_{8f(1)}$ (**(a)** and **(b)**); $O_{8f(2)}$ (**(c)**, **(d)**), and $O_{8f(3)}$ (**(e)**, **(f)**). The root-mean square error (rmse) and CV-score (r^2) were computed using k-fold cross-validation.

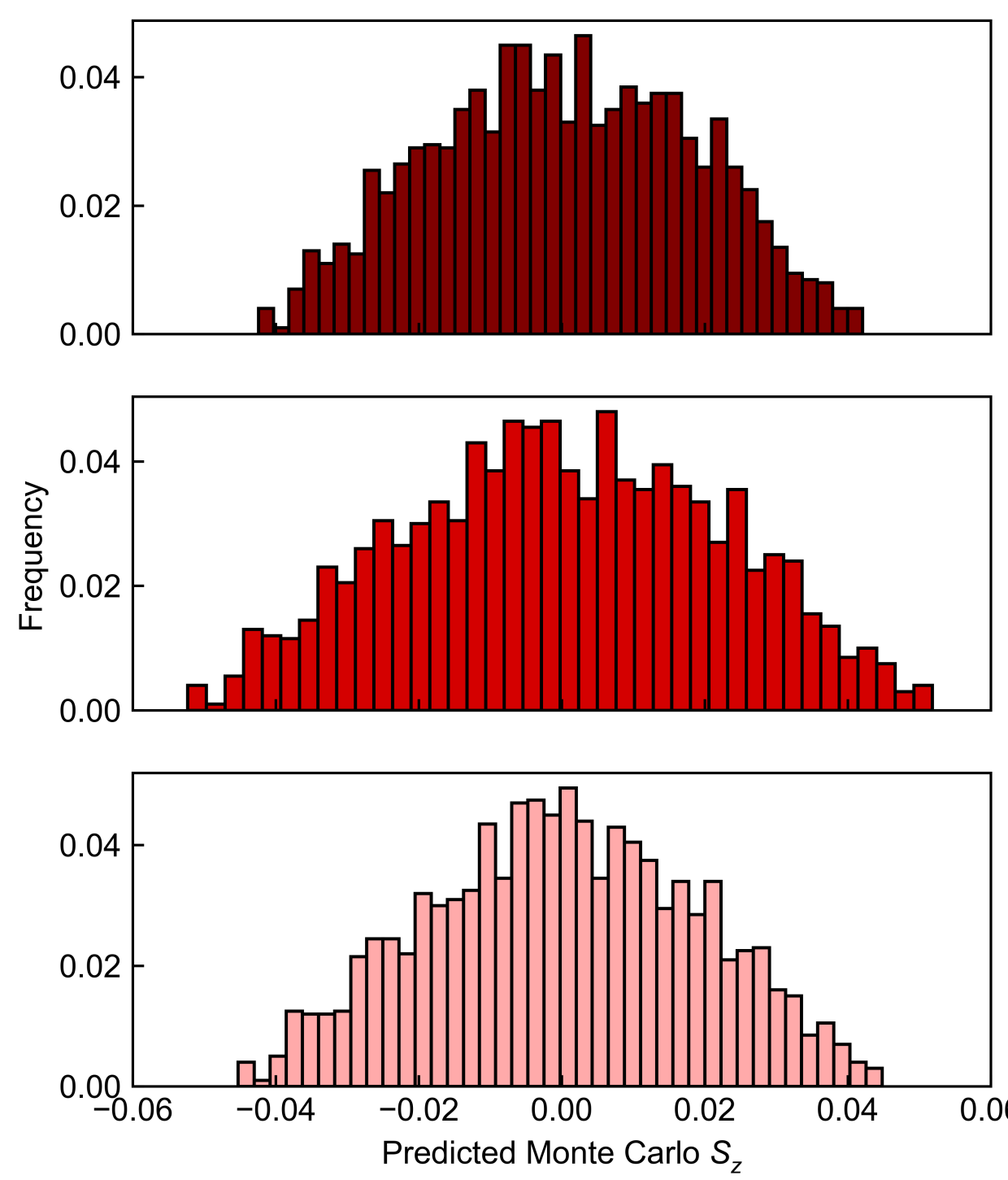

Figure S14: z-component of the spin, S_z, at ^{17}O nuclear positions, obtained from the Hyb20-based Heisenberg Monte Carlo (HMC) simulations on N-Li_2MnO_3. The spins on all O in the simulation cell are plotted as histograms: top, $O_{8f(1)}$, middle $O_{8f(2)}$ and bottom $O_{8f(3)}$.

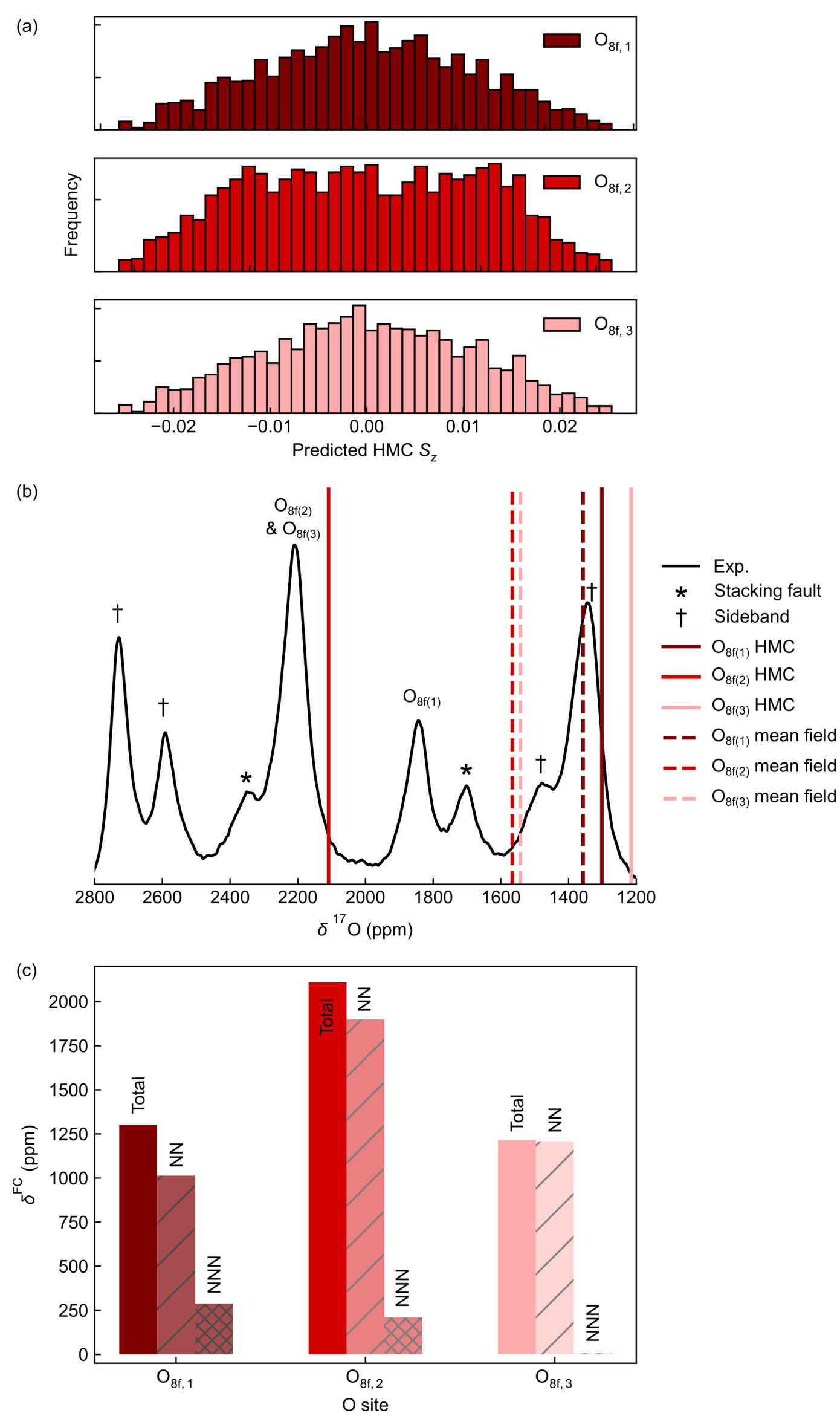

Figure S15: **(a)**: z-component of the electron spin at ^{17}O nuclear positions, predicted from Hyb35-based Heisenberg Monte Carlo (HMC) simulations on N-Li_2MnO_3. The Hyb35-based spins on all O in the simulation cell are plotted as histograms: top, $O_{8f(1)}$, middle $O_{8f(2)}$ and bottom $O_{8f(3)}$. **(b)** Numerical averages of the $O_{8f(1)}$, $O_{8f(2)}$, and $O_{8f(3)}$ shifts are shown alongside the mean-field calculated shifts (obtained using eq. 15), and the experimental spectrum, acquired at 2.35 T with a magic angle spinning frequency of 60 kHz, corresponding to a sample temperature of 320 K. **(c)** Decomposition of the average shifts into contributions from each coordination shell.

11 Structural Models of Li_2MnO_3 Used in This Study

Table S2: Rietveld-derived lattice parameters and site occupancies, based on powder neutron diffraction data collected for Li_2MnO_3 powder, $R_{\mathrm{w.p.}}$ = 9.1%. Standard errors are given for all refined values and are in parentheses; decimal quantities that were not refined are denoted with an asterisk.

space group				$C2/c$		
a (Å)	4.91717(3)		α (°)	90		
b (Å)	8.52454(3)		β (°)	99.728(5)		
c (Å)	9.60924(12)		γ (°)	90		
	site	*x*	*y*	*z*	occ.	B_{iso} (Å^2)
Mn(1)/Li(1)	4*e*	0	0.75065*	1/4	0.478/0.522	0.7*
Mn(2)/Li(2)	4*e*	0	0.41573*	1/4	0.859/0.141	0.7*
Mn(3)/Li(3)	4*e*	0	0.08141*	1/4	0.734/0.266	0.7*
Li(4)	8*f*	0.24685*	0.08694*	-0.00316*	1.0	0.7*
Li(5)	4*d*	1/4	1/4	1/2	1.0	0.7*
O(1)	8*f*	0.1079*	0.256*	0.1364*	1.0	0.5*
O(2)	8*f*	0.641*	0.0655*	0.1371*	1.0	0.5*
O(3)	8*f*	0.641*	0.4169*	0.1398*	1.0	0.5*

Table S3: Lattice parameters, ion positions and site occupancies, for the ferromagnetically relaxed structure of Li_2MnO_3.

space group				$P1$	
a (Å)	4.96132		α (°)	90	
b (Å)	8.57740		β (°)	99.46134	
c (Å)	9.65176		γ (°)	90	
		x	*y*	*z*	occ.
Li(1)		0.749632895	0.588779628	0.999865890	1
Li(2)		0.249374732	0.088231273	0.999760211	1
Li(3)		0.750614524	0.911680460	0.000001111	1
Li(4)		0.250706941	0.411315471	0.000491640	1
Li(5)		0.250367045	0.588779628	0.500134051	1
Li(6)		0.750625193	0.088231273	0.500239730	1
Li(7)		0.249385417	0.911680460	0.499998808	1
Li(8)		0.749292970	0.411315471	0.499508291	1
Li(9)		0.750136197	0.750165999	0.500000834	1
Li(10)		0.249751866	0.249835238	0.499932200	1
Li(11)		0.249863714	0.750165999	0.999999106	1
Li(12)		0.750248075	0.249835238	0.000067721	1
Li(13)		0.000000000	0.749840558	0.249999970	1
Li(14)		0.499999970	0.249742046	0.249999970	1
Li(15)		0.499999970	0.750158846	0.749999940	1
Li(16)		0.000000000	0.250360519	0.749999940	1
Mn(1)		0.499999970	0.917005718	0.249999970	1
Mn(2)		0.000000000	0.417043149	0.249999970	1
Mn(3)		0.000000000	0.582781792	0.749999940	1
Mn(4)		0.499999970	0.083296679	0.749999940	1
Mn(5)		0.499999970	0.582724333	0.249999970	1
Mn(6)		0.000000000	0.082693584	0.249999970	1
Mn(7)		0.000000000	0.917316079	0.749999940	1
Mn(8)		0.499999970	0.417185724	0.749999940	1
O(1)		0.605468869	0.749835014	0.136239871	1
O(2)		0.105597951	0.249850109	0.136258304	1
O(3)		0.894875288	0.750327587	0.863895059	1
O(4)		0.395135880	0.249984935	0.863733351	1
O(5)		0.394531071	0.749835014	0.363760054	1
O(6)		0.894401968	0.249850109	0.363741636	1
O(7)		0.105124630	0.750327587	0.636104882	1
O(8)		0.604864061	0.249984935	0.636266589	1
O(9)		0.142545730	0.571108937	0.138193786	1
O(10)		0.642554343	0.071097374	0.138184607	1
O(11)		0.357677937	0.928951621	0.861799121	1
O(12)		0.856542528	0.429386616	0.861746192	1
O(13)		0.857454181	0.571108937	0.361806154	1
O(14)		0.357445568	0.071097374	0.361815304	1
O(15)		0.642322004	0.928951621	0.638200819	1
O(16)		0.143457383	0.429386616	0.638253748	1
O(17)		0.142514139	0.928517044	0.138158664	1
O(18)		0.642519951	0.428524435	0.138129830	1
O(19)		0.356397420	0.570858359	0.861674011	1
O(20)		0.857940137	0.071474589	0.861955702	1
O(21)		0.857485771	0.928517044	0.361841261	1
O(22)		0.357479990	0.428524435	0.361870110	1
O(23)		0.643602490	0.570858359	0.638325870	1
O(24)		0.142059818	0.071474589	0.638044238	1

Table S4: Lattice parameters, ion positions and site occupancies, for the anti-ferromagnetically relaxed structure of Li_2MnO_3.

space group				$P1$	
a (Å)	4.90484		α (°)	90	
b (Å)	8.49032		β (°)	99.53389	
c (Å)	9.54907		γ (°)	90	
		x	y	z	occ.
Li(1)		0.74959685	0.58892555	0.99988146	1
Li(2)		0.24969307	0.08890932	0.99987790	1
Li(3)		0.75022970	0.91110886	0.99999539	1
Li(4)		0.25063232	0.41098306	0.00030146	1
Li(5)		0.25040307	0.58892555	0.50011846	1
Li(6)		0.75030685	0.08890932	0.50012202	1
Li(7)		0.24977022	0.91110886	0.50000452	1
Li(8)		0.74936760	0.41098306	0.49969846	1
Li(9)		0.75000797	0.74999651	0.50002689	1
Li(10)		0.25000021	0.25001001	0.49990583	1
Li(11)		0.24999195	0.74999651	0.99997303	1
Li(12)		0.74999971	0.25001001	0.00009409	1
Li(13)		0.99999996	0.74998732	0.24999996	1
Li(14)		0.49999996	0.24998257	0.24999996	1
Li(15)		0.49999996	0.75001406	0.74999996	1
Li(16)		0.99999996	0.25001833	0.74999996	1
Mn(1)		0.49999996	0.91671279	0.24999996	1
Mn(2)		0.99999996	0.41673513	0.24999996	1
Mn(3)		0.99999996	0.58327272	0.74999996	1
Mn(4)		0.49999996	0.08329780	0.74999996	1
Mn(5)		0.49999996	0.58303101	0.24999996	1
Mn(6)		0.99999996	0.08296799	0.24999996	1
Mn(7)		0.99999996	0.91700664	0.74999996	1
Mn(8)		0.49999996	0.41701666	0.74999996	1
O(1)		0.60599495	0.74986608	0.13606112	1
O(2)		0.10604510	0.24986081	0.13607293	1
O(3)		0.89397963	0.75015352	0.86393335	1
O(4)		0.39401836	0.25013584	0.86395921	1
O(5)		0.39400497	0.74986608	0.36393879	1
O(6)		0.89395482	0.24986081	0.36392699	1
O(7)		0.10602029	0.75015352	0.63606657	1
O(8)		0.60598156	0.25013584	0.63604071	1
O(9)		0.14192680	0.57147019	0.13796261	1
O(10)		0.64193002	0.07146657	0.13794665	1
O(11)		0.35808371	0.92853924	0.86204681	1
O(12)		0.85806249	0.42854066	0.86204980	1
O(13)		0.85807312	0.57147019	0.36203730	1
O(14)		0.35806990	0.07146657	0.36205327	1
O(15)		0.64191621	0.92853924	0.63795310	1
O(16)		0.14193743	0.42854066	0.63795012	1
O(17)		0.14192783	0.92826275	0.13792055	1
O(18)		0.64193310	0.42825155	0.13792417	1
O(19)		0.35807108	0.57172415	0.86209438	1
O(20)		0.85807454	0.07177282	0.86207674	1
O(21)		0.85807209	0.92826275	0.36207937	1
O(22)		0.35806682	0.42825155	0.36207575	1
O(23)		0.64192884	0.57172415	0.63790554	1
O(24)		0.14192539	0.07177282	0.63792318	1

Table S5: Lattice parameters, ion positions and site occupancies, for the anti-ferromagnetically relaxed structure of Li_2MnO_3.

space group				$P1$	
a (Å)	4.80300		α (°)	60	
b (Å)	4.92120		β (°)	85.2812	
c (Å)	4.92120		γ (°)	80.5300	
		x	*y*	*z*	occ.
Li(1)		0.00000000	0.00000000	0.00000000	1
Li(2)		0.50000000	0.83333333	0.33333333	1
Li(3)		0.50000000	0.16666667	0.66666667	1
Li(4)		0.50000000	0.50000000	0.00000000	1
Mn(1)		0.00000000	0.33333333	0.33333333	1
Mn(2)		0.00000000	0.66666667	0.66666667	1
O(1)		0.22600005	0.58333333	0.3333333	1
O(2)		0.77399999	0.41666667	0.6666666	1
O(3)		0.77399999	0.08333333	0.3333333	1
O(4)		0.22600005	0.91666667	0.6666666	1
O(5)		0.22600005	0.25000000	0.0000000	1
O(6)		0.77399999	0.75000000	0.0000000	1

We note that the volumes expressed in Table 2 of the main text are expressed as the cell volume normalized by the number of Mn centers, to ensure comparability.

12 Effect of Normal Modes on the Fermi Contact Shifts for ^{7}Li and ^{17}O

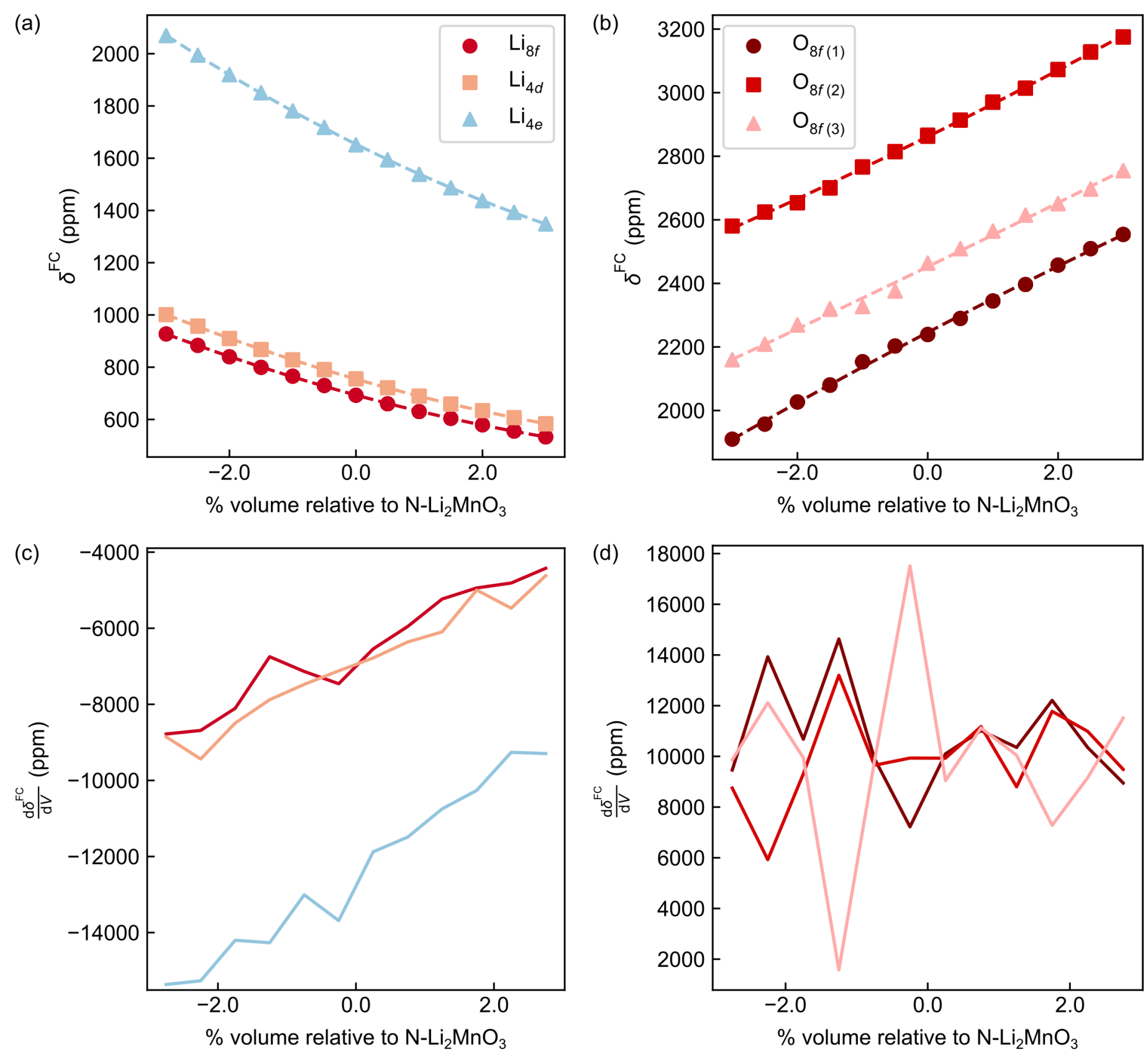

Figure S16: Variation in the calculated Fermi contact shift for ^{7}Li ((**a**) and (**b**)) and ^{17}O ((**c**) and (**d**)) with volume of the simulation cell. The simulation cell at 0.0% volume change corresponds to N-Li_2MnO_3. For all other cells, the atomic coordinates were held fixed while the volume was varied uniformly. In (**c**) and (**d**), the derivative of the shift with respect to the volume change is shown for ^{7}Li and ^{17}O, respectively. This can be considered analogous to a thermal expansion coefficient. All shifts were calculated using the Hyb20 functional and scaled to room temperature using the Curie–Weiss scaling in eq. 15 ($\theta = -45$ K, $\mu_{\text{eff}} = 3.87\ \mu_B\ \text{mol}_{\text{Mn}}{}^{-1}$).

13 Effect of Harmonic Lattice Vibrations on Room-Temperature Fermi Contact Shifts

In this section, we derive expressions describing the impact of harmonic lattice vibrations on finite-temperature Fermi contact shifts. As stated in the main text, as a full cluster expansion treatment of vibrations and magnetism are required to describe the Fermi contact shift, we chose to focus on the impact on the value of S_z at local ^{7}Li and ^{17}O sites in N-Li_2MnO_3 in the ferromagnetic configuration.

Consider a supercell of a crystal containing N atoms. Each atom, i, has a displacement vector, $\vec{u}_i$, relative to a high symmetry reference state. These can be renormalized by the square root of the mass m_i of atom i to yield the displacement vectors $\vec{v}_i = \vec{u}_i\sqrt{m_i}$. Each vector $\vec{v}_i$ has three Cartesian components, $v_{i,x}$, $v_{i,y}$, $v_{i,z}$. The displacement vectors for all N atoms of the supercell can be concatenated into a $3N$-dimensional vector $\vec{v} = (v_{1,x}, v_{1,y}, v_{1,z}, \ldots, v_{i,x}, \ldots, v_{N,z})$. The $3N$ dimensional vector of displacements $\vec{v}$ can next be decomposed into a sum of $3N$ symmetry-adapted orthogonal normal modes using group theoretical techniques, implemented in the CASM software package,[17,37] according to

$$\vec{v} = \sum_{n=1}^{3N} \eta_n \vec{q}_n \tag{5}$$

where $\vec{q}_n$ are $3N$ dimensional vectors that represent symmetry adapted collective displacement modes. The normal mode vectors form a complete and orthonormal set, i.e. $\vec{q}_n^{\intercal}\vec{q}_m = \delta_{n,m}$, where $\delta_{n,m}$ is the Kronecker delta. In the above sum, 3 normal modes correspond to rigid translation translations.

Within the harmonic approximation, the normal modes $\vec{q}_n$ diagonalize the potential energy of the $3N$ vibrational degrees of freedom of the supercell. The potential energy of the crystal can then be expressed in terms of the normal amplitudes η_n and decomposes into a sum of independent harmonic oscillators. Each independent harmonic oscillator potential takes the form

(we drop the subscript n and focus on each mode independently)

$$V(\eta) = \frac{1}{2}\omega^2\eta^2, \tag{6}$$

where ω^2 is the natural frequency of the mode and corresponds to the curvature of the energy of the crystal as a function of η.

We next consider the effect of symmetry adapted collective vibration modes with amplitudes η on the local spin at the NMR nucleus of interest. Assuming that the effect of lattice vibrations on the z-component of the electron spin at the nucleus, S_z, is small, we may expand S_z as:

$$S_z = S_{z,0} + \left(\frac{\mathrm{d}S_z}{\mathrm{d}\eta}\right)_{\eta=0}\eta + \frac{1}{2}\left(\frac{\mathrm{d}S_z^2}{\mathrm{d}\eta^2}\right)_{\eta=0}\eta^2 + ... \tag{7}$$

$$= S_{z,0} + \Delta_1\eta + \frac{1}{2}\Delta_2\eta^2 + ..., \tag{8}$$

where $S_{z,0}$ is the z-component of the electron spin at the nucleus in the high symmetry reference structure, corresponding here to the atomic positions of N-Li_2MnO_3. In the second line, we have absorbed the derivatives into the coefficients of the Taylor series. The thermal average of S_z is then given by:

$$\langle S_z(q)\rangle = S_{z,0} + \Delta_1\langle\eta\rangle + \Delta_2\langle\eta^2\rangle + ... \tag{9}$$

To evaluate the thermal average of S_z, we first require the partition function, Z, associated with the harmonic potential in eq. 6:

$$Z = \int_{-\infty}^{\infty} \exp\left(-\frac{1}{2}\beta\omega^2\eta^2\right)\mathrm{d}\eta \tag{10}$$

$$= \left(\frac{2\pi}{\beta\omega^2}\right)^{1/2}, \tag{11}$$

where $\beta = 1/k_{\mathrm{B}}T$. The thermal average of η is:

$$\langle \eta \rangle = \int_{-\infty}^{\infty} \eta \left(\frac{2\pi}{\beta \omega^2} \right)^{1/2} \exp \left(-\frac{1}{2} \beta \omega^2 \eta^2 \right) \tag{12}$$

$$= 0 \tag{13}$$

where we have exploited the fact that the integrand is an odd function and the integration limits are symmetric about the origin. The thermal average of η^2 is then:

$$\langle \eta^2 \rangle = \int_{-\infty}^{\infty} \eta^2 \left(\frac{2\pi}{\beta \omega^2} \right)^{1/2} \exp \left(-\frac{1}{2} \beta \omega^2 \eta^2 \right) \tag{14}$$

$$= \frac{1}{\beta \omega^2}. \tag{15}$$

Using the expressions derived above, the root-mean-square thermal displacement for a given normal mode can be obtained simply by fitting the energy as a function of displacement:

$$\langle \eta^2 \rangle^{\frac{1}{2}} = \frac{1}{(\beta \omega^2)^{\frac{1}{2}}}. \tag{16}$$

The thermally-averaged shift contribution due to the lattice vibrational mode is given by:

$$\langle S_z(\eta) \rangle = S_{z,0} + \frac{1}{2} \Delta_2 \left(\frac{1}{\beta \omega^2} \right). \tag{17}$$

Therefore, if the spin density (and hence the Fermi contact shift) is linear with respect to the vibrational mode amplitude over the relevant range of displacements, $\Delta_2 = 0$ and the thermal average of S_z vanishes. If, instead, the shift exhibits a quadratic dependence on the mode amplitude, eq. 17 is recovered.

This means that, by fitting the variation of the energy with respect to η and the shift with respect to η, we can obtain the vibrational contribution to the shift, $\frac{1}{2} \Delta_2 \left(\frac{1}{\beta \omega^2} \right)$.

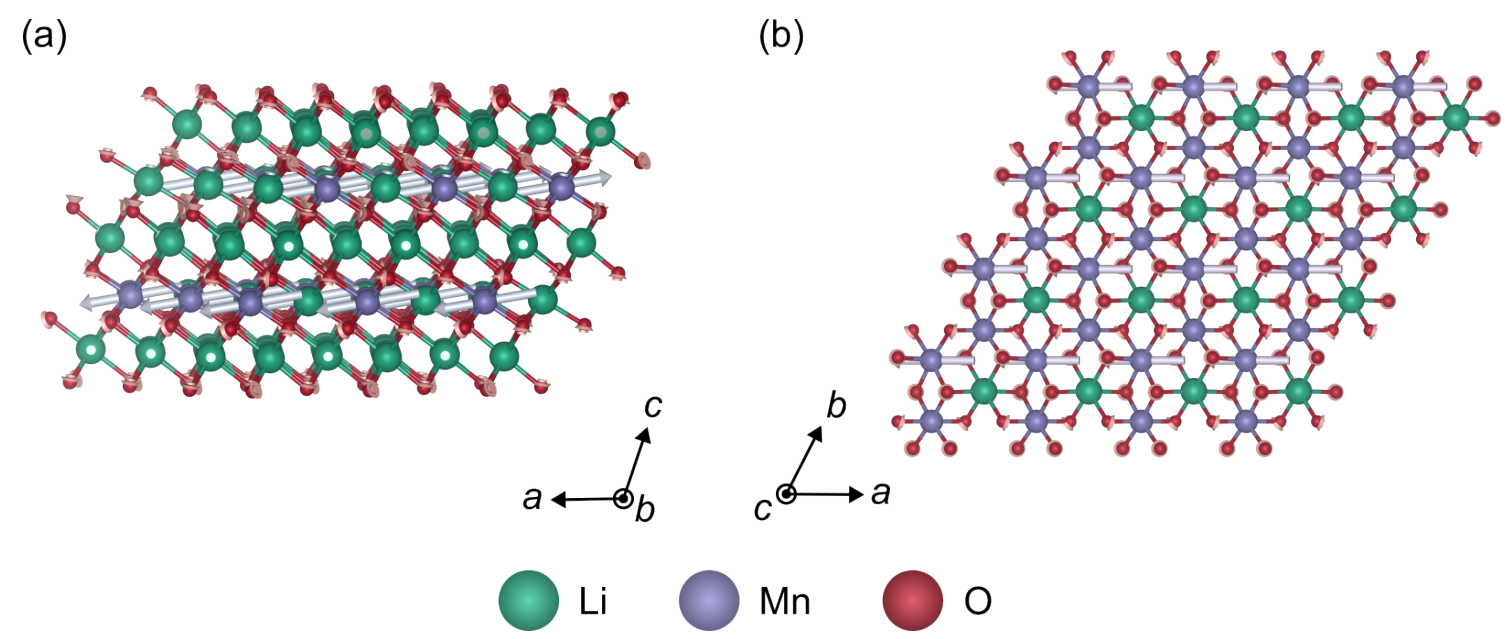

Figure S17: Example of a symmetry-adapted harmonic vibrational mode in N-Li_2MnO_3, corresponding to mode 1 described in the main text. The mode is illustrated from **(a)** a side view, and **(b)** a top view of a single [$Li_{1/3}Mn_{2/3}$] layer.

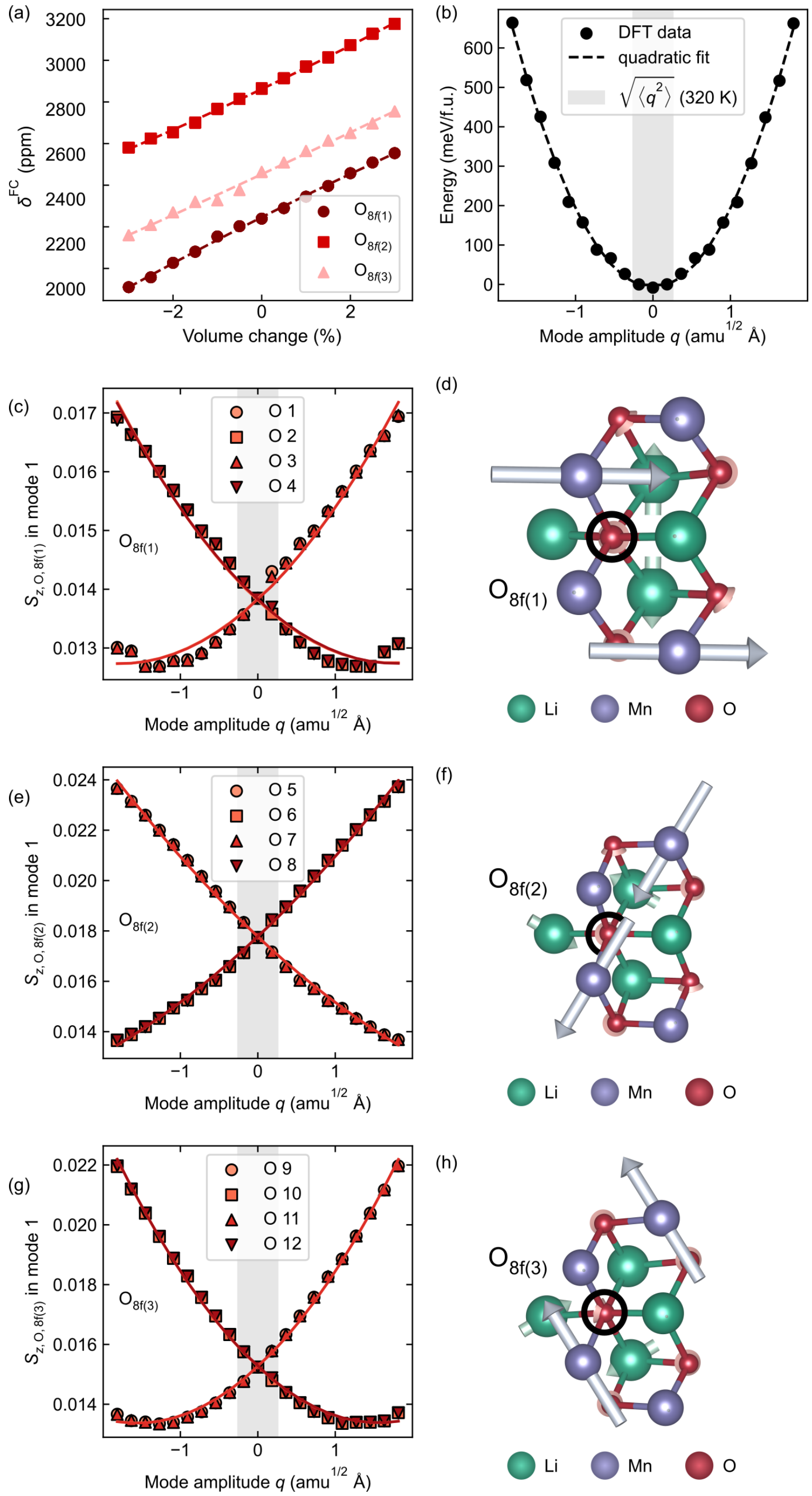

Figure S18: Impact of volume and atomic displacements (vibrational modes) on the calculated ^{17}O z-component of spin density, S_z. All first-principles ^{17}O spin densities were computed using the Hyb20 functional. **(a)** Change in ^{17}O S_z as a function of cell volume for the N-Li_2MnO_3 structure. **(b)** Variation in the total energy with normal mode displacement amplitude for mode 1. The corresponding variations in S_z of $O_{8f(1)}$, $O_{8f(2)}$ and $O_{8f(3)}$ are shown in **(c)** and **(d)**, **(e)** and **(f)**, and **(g)** and **(h)**, respectively. The light gray boxes in **(b)**, **(c)**, **(e)** and **(g)** indicate the displacement range expected at room temperature.

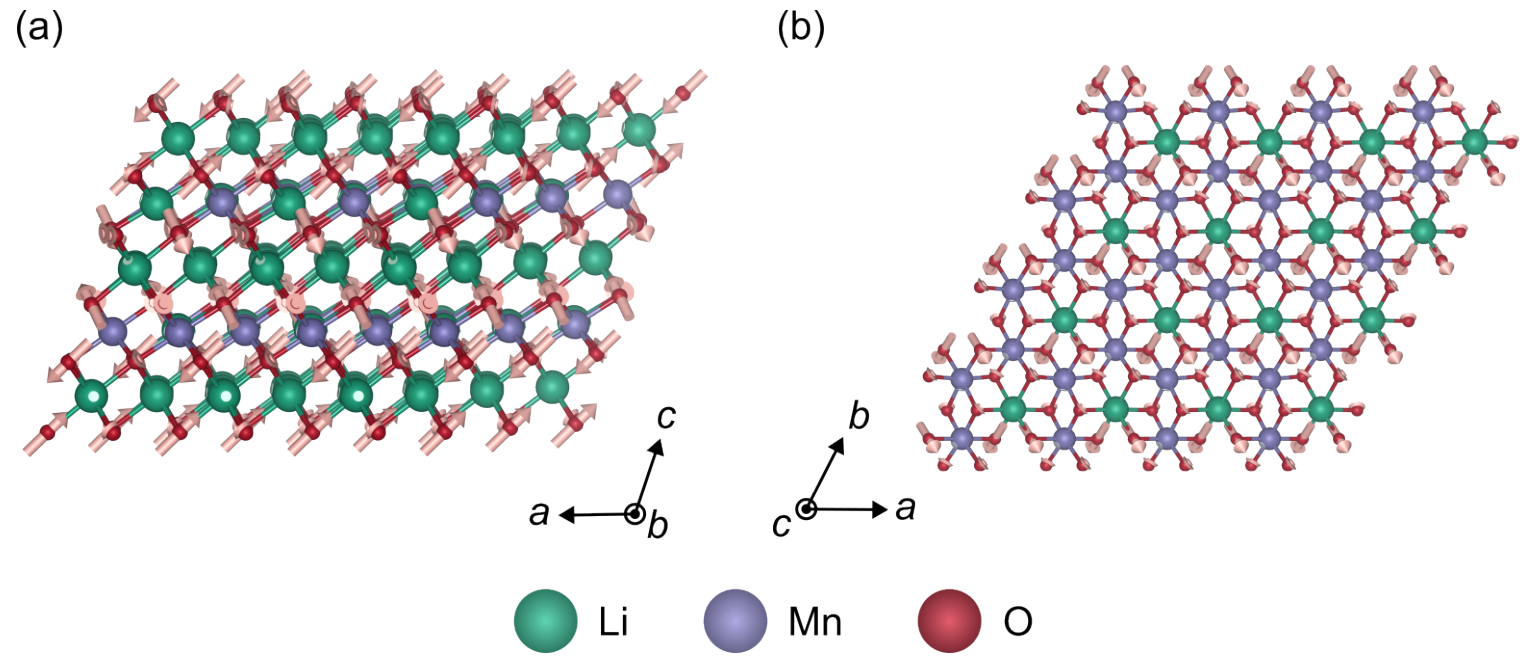

Figure S19: Symmetry-adapted displacement mode 2 for N-Li_2MnO_3 described in the main text. This mode corresponds to the mode shown in the main text (Figures S21 and S22). **(a)** shows the layer stacking whilst **(b)** shows a top-down view of a single [$Li_{1/3}Mn_{2/3}$] layer.

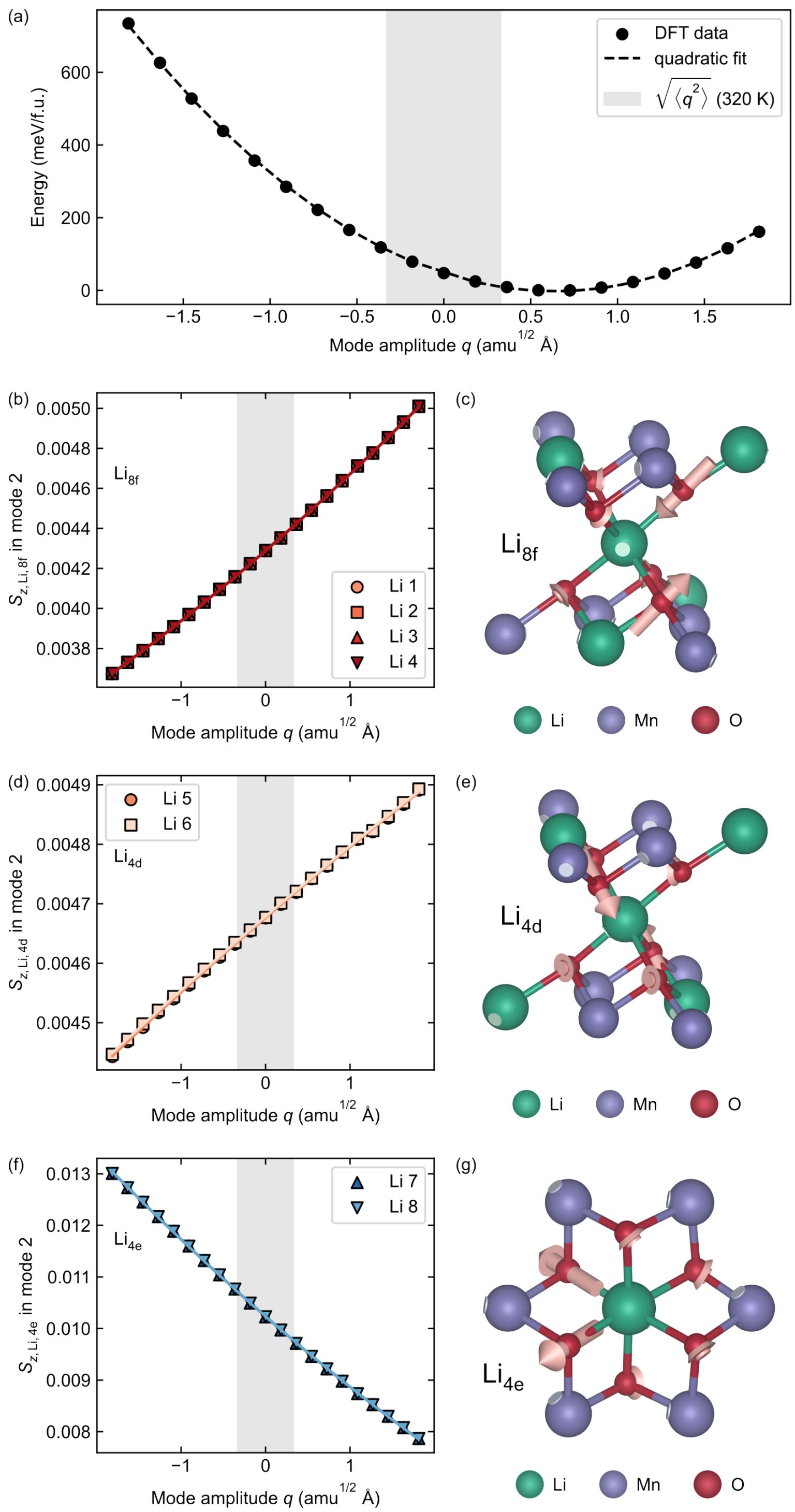

Figure S20: Impact of atomic displacements (vibrational modes) on the calculated ^{7}Li z-component of spin density, S_z. All first-principles ^{7}Li spin densities were computed using the Hyb20 functional. **(a)** Variation in the total energy with normal mode displacement amplitude for mode 2. The corresponding variations in the S_z of Li_{8f}, Li_{4d} and Li_{4e} sites along the same mode are shown in **(b)** and **(c)**, **(d)** and **(e)**, and **(f)** and **(g)**, respectively. The light gray boxes in **(a)**, **(b)**, **(d)** and **(f)** indicate the displacement range expected at room temperature.

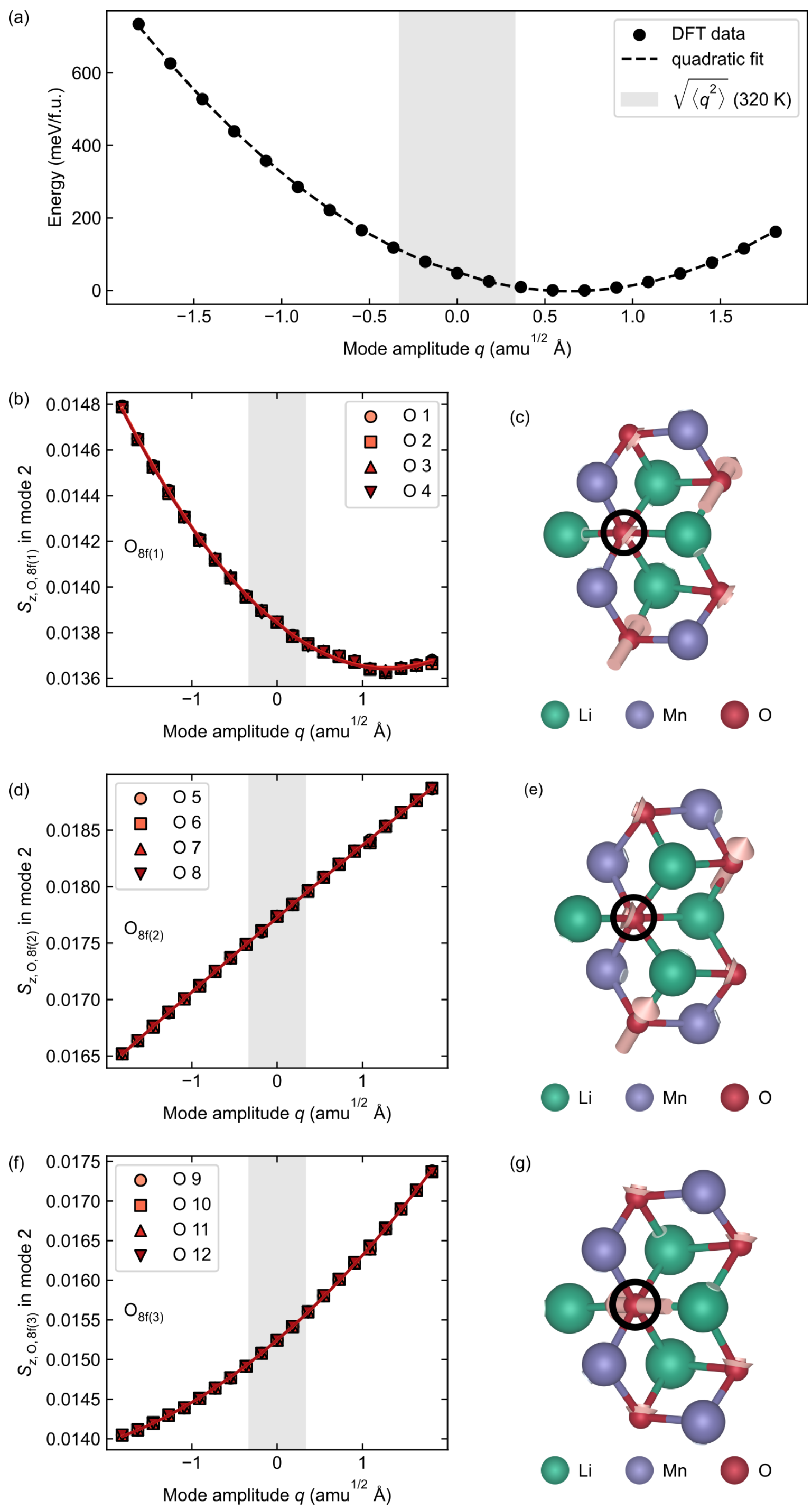

Figure S21: Impact of atomic displacements (vibrational modes) on the calculated ^{17}O z-component of spin density, S_z. All first-principles ^7Li spin densities were computed using the Hyb20 functional. **(a)** Variation in the total energy with normal mode displacement amplitude for mode 2. The corresponding variations in the S_z of $O_{8f(1)}$, $O_{8f(2)}$ and $O_{8f(3)}$ sites along the same mode are shown in **(b)** and **(c)**, **(d)** and **(e)**, and **(f)** and **(g)**, respectively. The light gray boxes in **(a)**, **(b)**, **(d)** and **(f)** indicate the displacement range expected at room temperature.

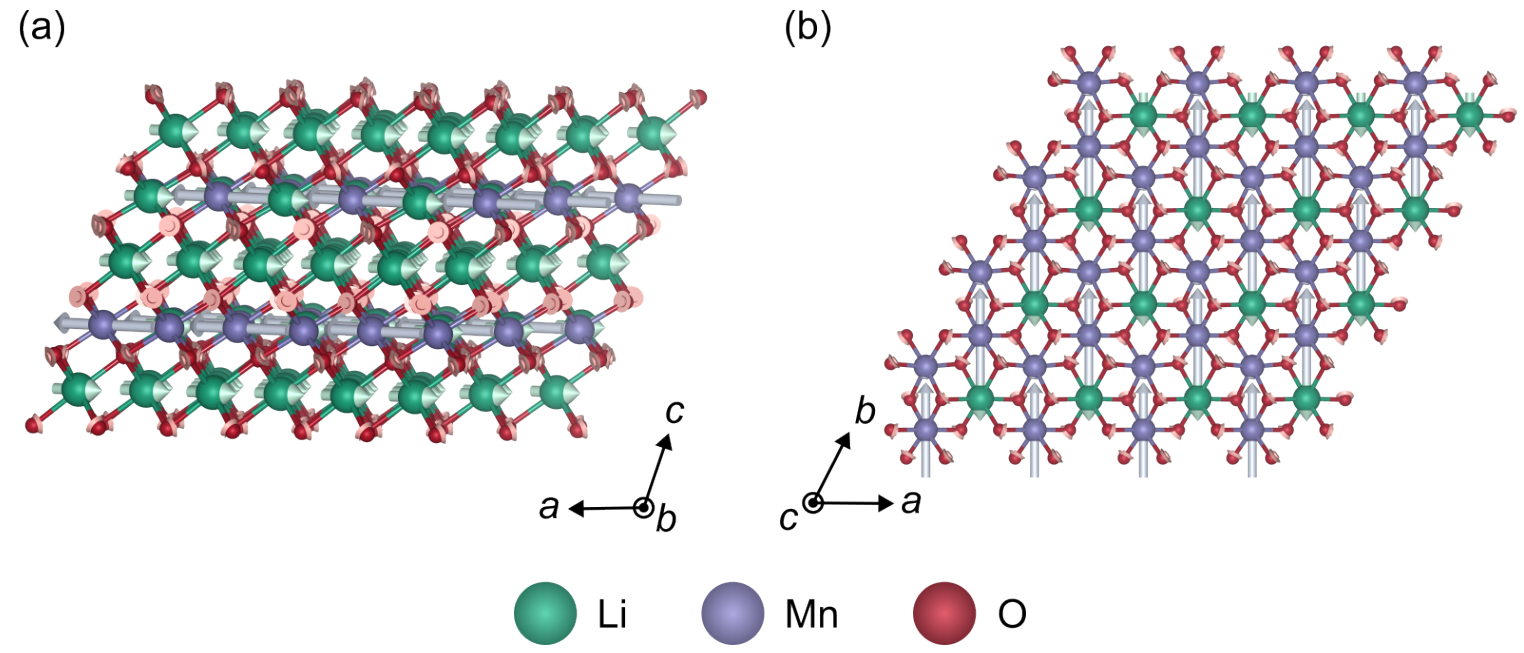

Figure S22: Symmetry-adapted displacement mode 3 for N-Li_2MnO_3 described in the main text. This mode corresponds to the mode shown in the main text (Figures S23 and S24). **(a)** shows the layer stacking whilst **(b)** shows a top-down view of a single [$Li_{1/3}Mn_{2/3}$] layer.

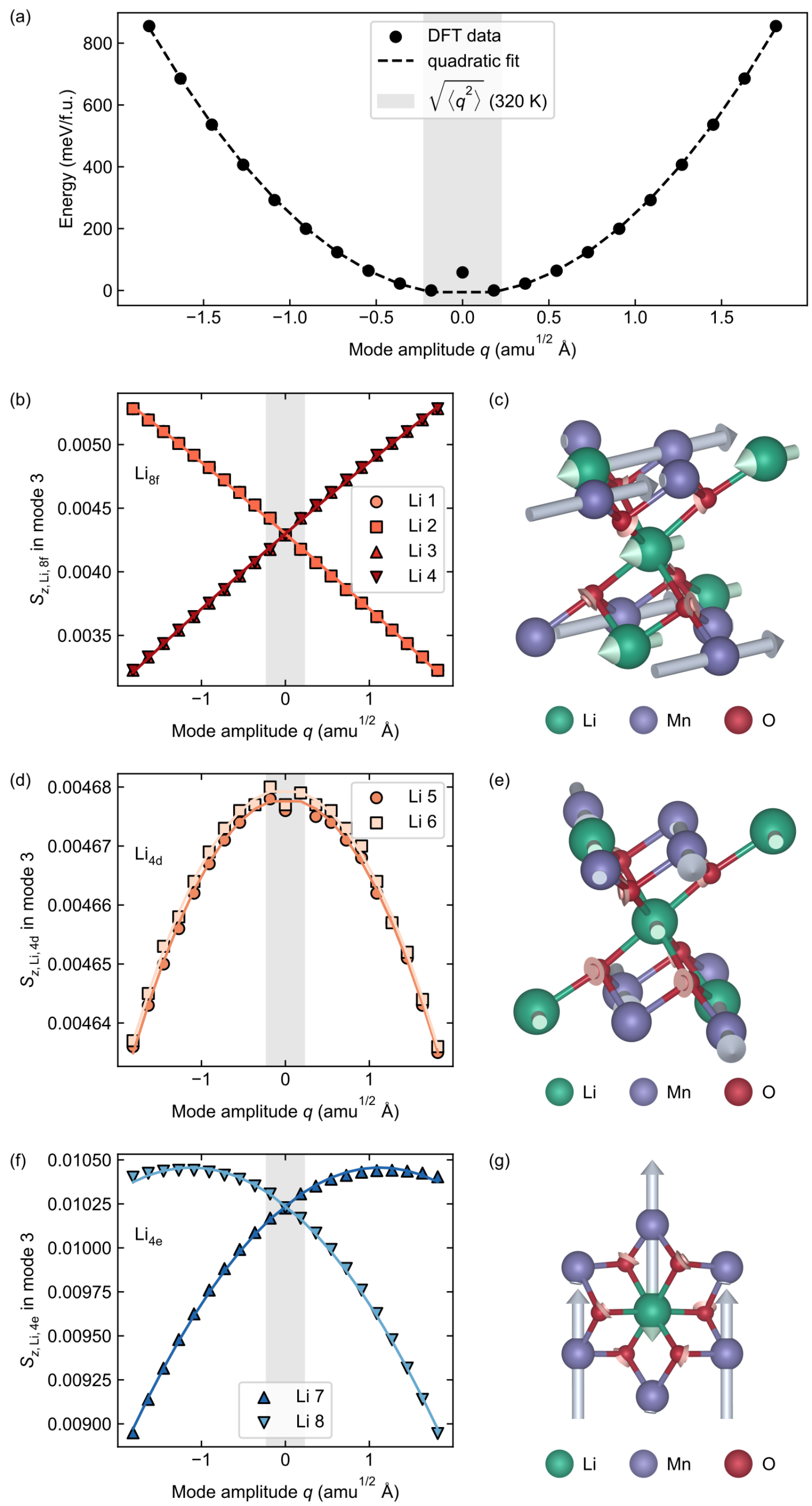

Figure S23: Impact of atomic displacements (vibrational modes) on the calculated ^{7}Li z-component of spin density, S_z. All first-principles ^{7}Li spin densities were computed using the Hyb20 functional. **(a)** Variation in the total energy with normal mode displacement amplitude for mode 2. The corresponding variations in the S_z of Li_{8f}, Li_{4d} and Li_{4e} sites along the same mode are shown in **(b)** and **(c)**, **(d)** and **(e)**, and **(f)** and **(g)**, respectively. The light gray boxes in **(a)**, **(b)**, **(d)** and **(f)** indicate the displacement range expected at room temperature.

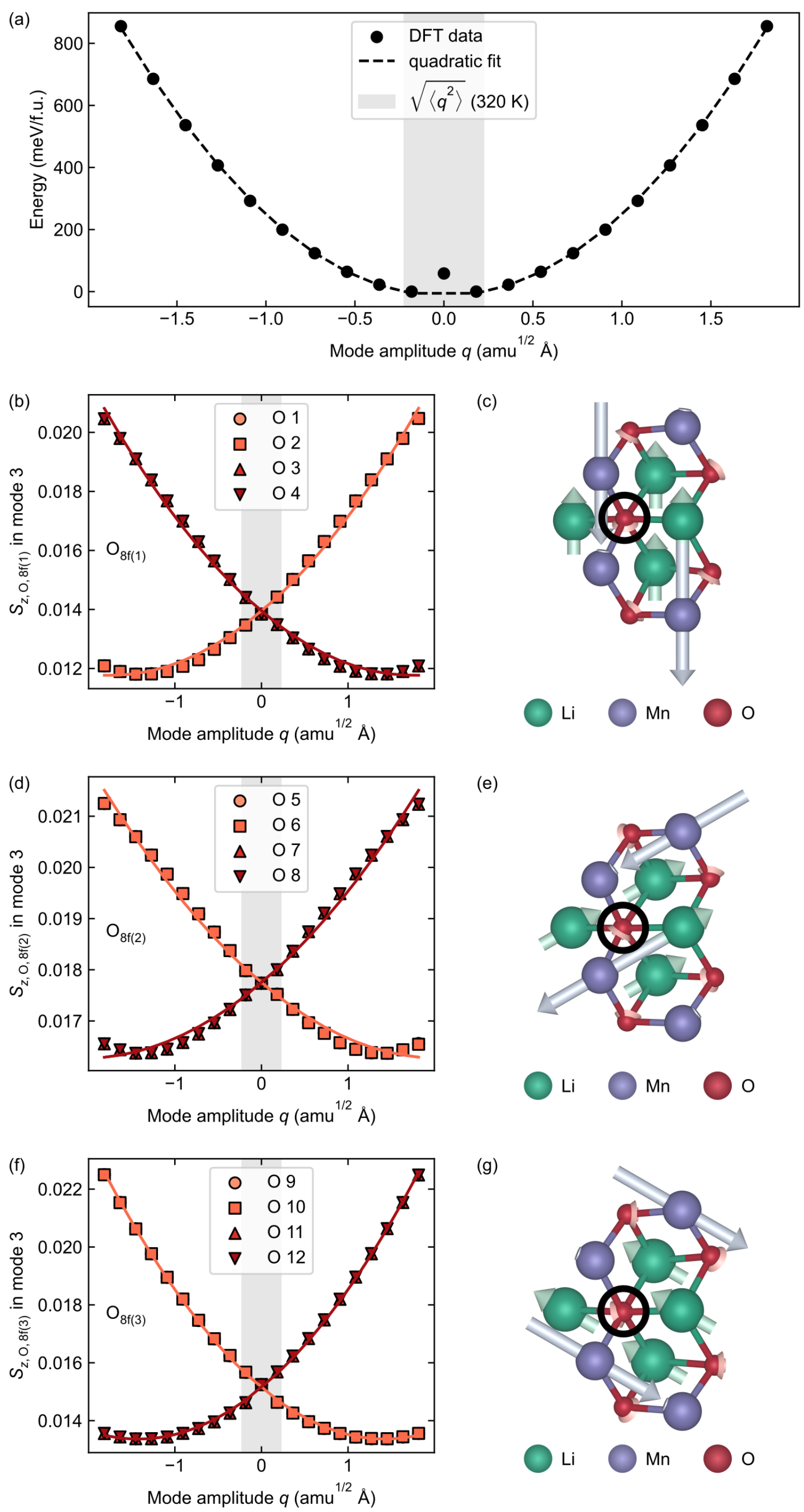

Figure S24: Impact of atomic displacements (vibrational modes) on the calculated ^{17}O z-component of spin density, S_z. All first-principles ^{7}Li spin densities were computed using the Hyb20 functional. **(a)** Variation in the total energy with normal mode displacement amplitude for mode 2. The corresponding variations in the S_z of $O_{8f(1)}$, $O_{8f(2)}$ and $O_{8f(3)}$ sites along the same mode are shown in **(b)** and **(c)**, **(d)** and **(e)**, and **(f)** and **(g)**, respectively. The light gray boxes in **(a)**, **(b)**, **(d)** and **(f)** indicate the displacement range expected at room temperature.

14 Effect of Normal Mode Displacements on Local Structure around Li and O

Here, we show local structural parameters related to the structures in each of the normal mode displaced configurations generated by CASM.[17]

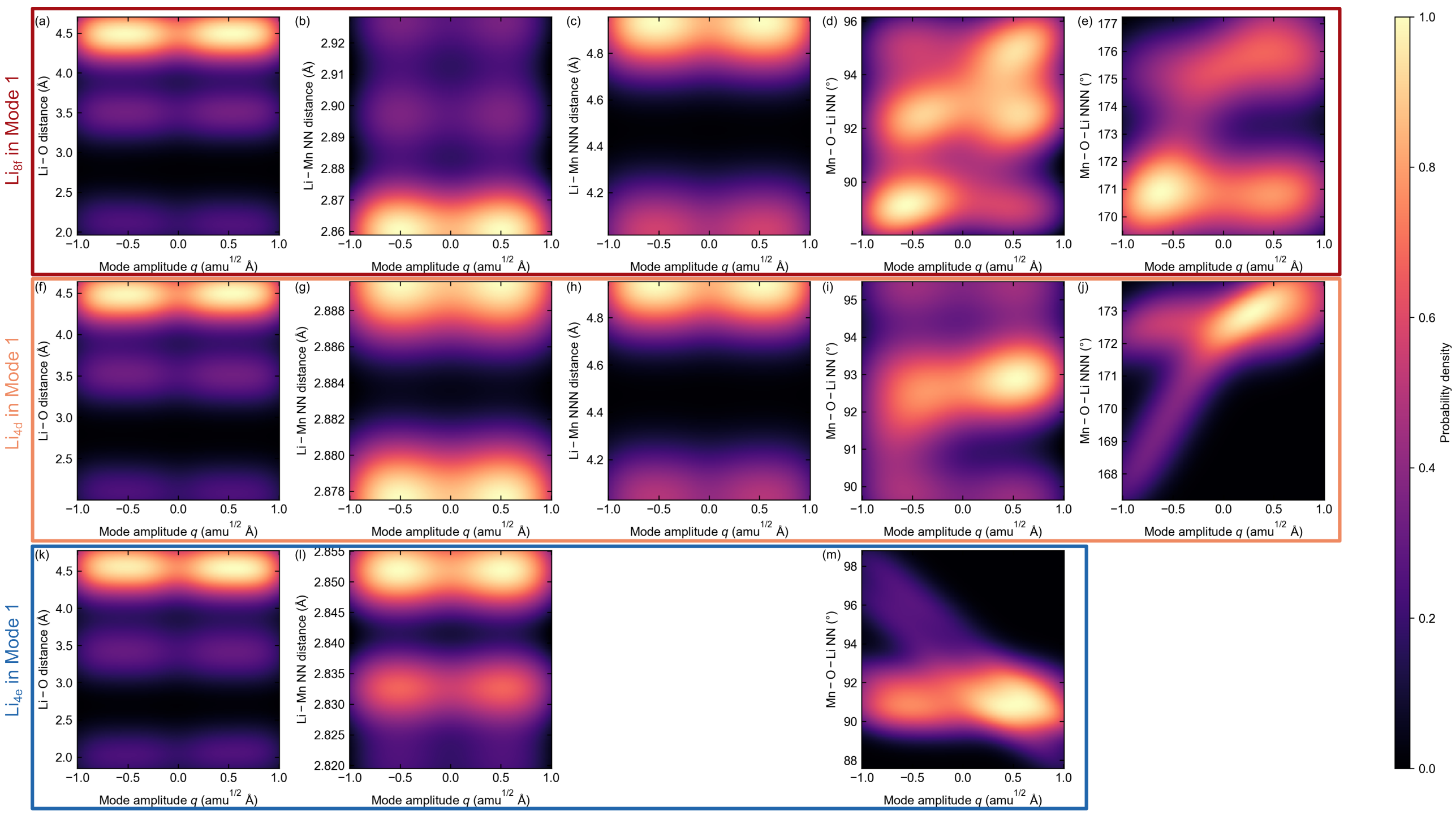

Figure S25: Impact of atomic displacements corresponding to vibrational mode 1 on the local structure around Li_{8f} (a) to (e), Li_{4d} (f) to (j) and Li_{4e} (k) to (m). For each crystallographic Li environment, we show the Li-O distances ((a), (f) and (k)), the Li-Mn nearest-neighbor (NN) distances ((b), (g) and (l)), the Li-Mn next-nearest-neighbor (NNN) distances ((c) and (g) - no such distances were considered for Li_{4e}, as per the magnetic cluster expansion in the main text), the Mn-O-Li NN bond angle ((d), (i) and (m)) and the Mn-O-Li NNN bond angle ((e) and (j), again no such NNN considered for Li_{4e}).

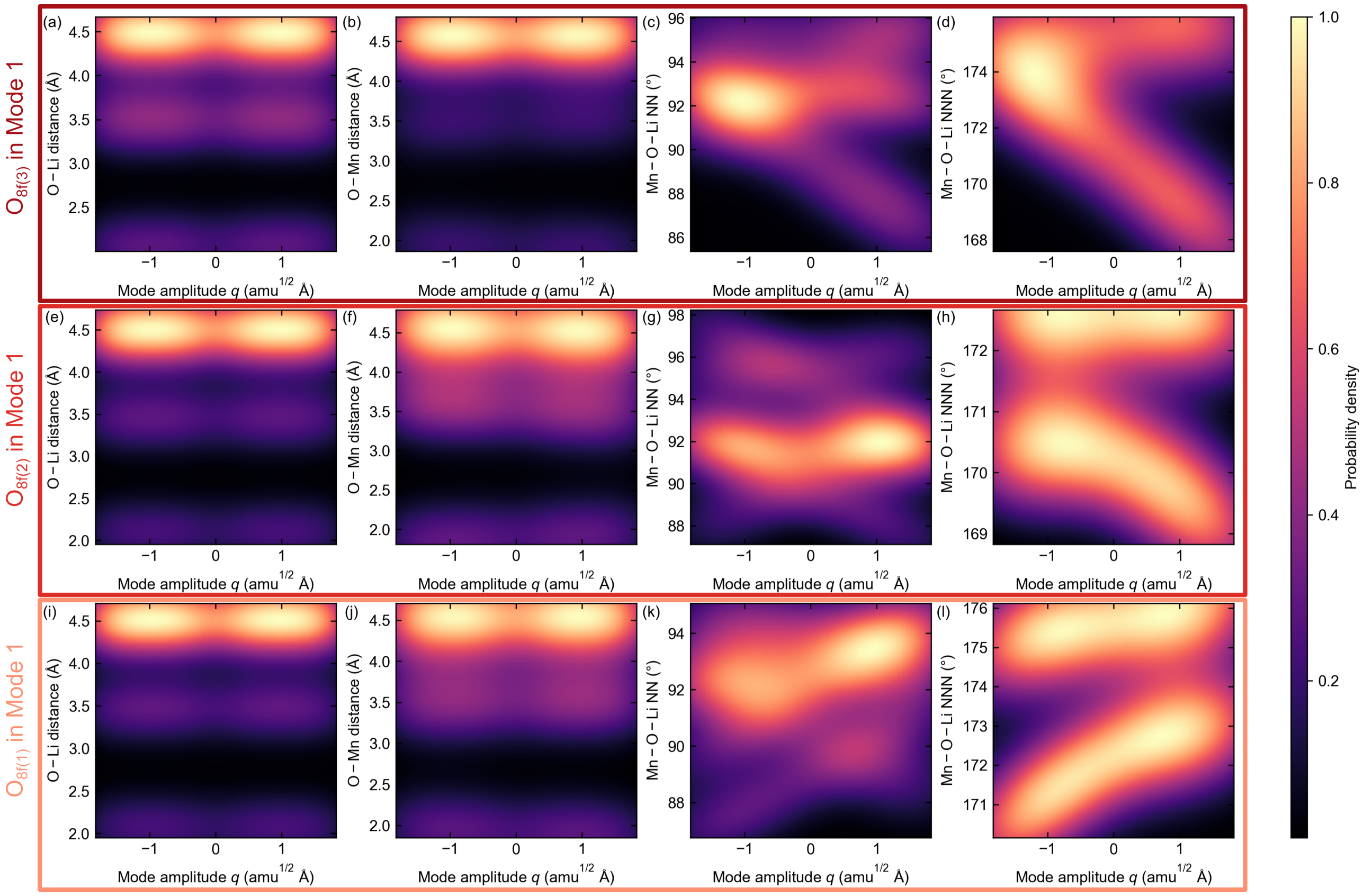

Figure S26: Impact of atomic displacements corresponding to vibrational mode 1 on the local structure around $O_{8f(1)}$ (a) to (d), $O_{8f(2)}$ (e) to (h) and $O_{8f(3)}$ (i) to (l). For each crystallographic O environment, we show the O-Li distances ((a), (e) and (i)), the O-Mn distances ((b), (f) and (j)), the Mn-O-Li nearest-neighbor (NN) bond angle ((c), (g) and (k)) and the Mn-O-Li next-nearest-neighbor (NNN) bond angle ((d), (h) and (l)).

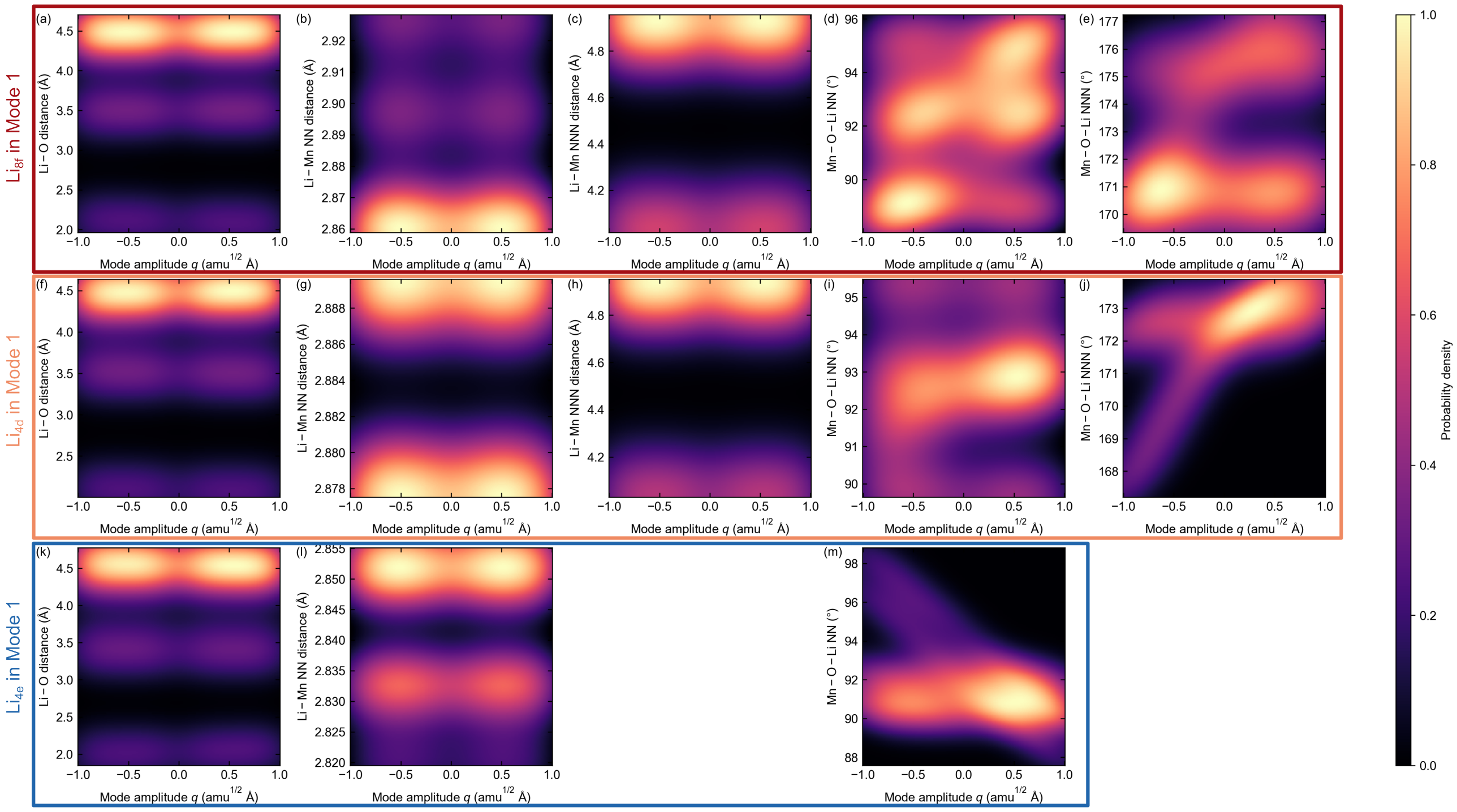

Figure S27: Impact of atomic displacements corresponding to vibrational mode 2 on the local structure around Li$_{8f}$ (a) to (e), Li$_{4d}$ (f) to (j) and Li$_{4e}$ (k) to (m). For each crystallographic Li environment, we show the Li-O distances ((a), (f) and (k)), the Li-Mn nearest-neighbor (NN) distances ((b), (g) and (l)), the Li-Mn next-nearest-neighbor (NNN) distances ((c) and (g) - no such distances were considered for Li$_{4e}$, as per the magnetic cluster expansion in the main text), the Mn-O-Li NN bond angle ((d), (i) and (m)) and the Mn-O-Li NNN bond angle ((e) and (j), again no such NNN considered for Li$_{4e}$).

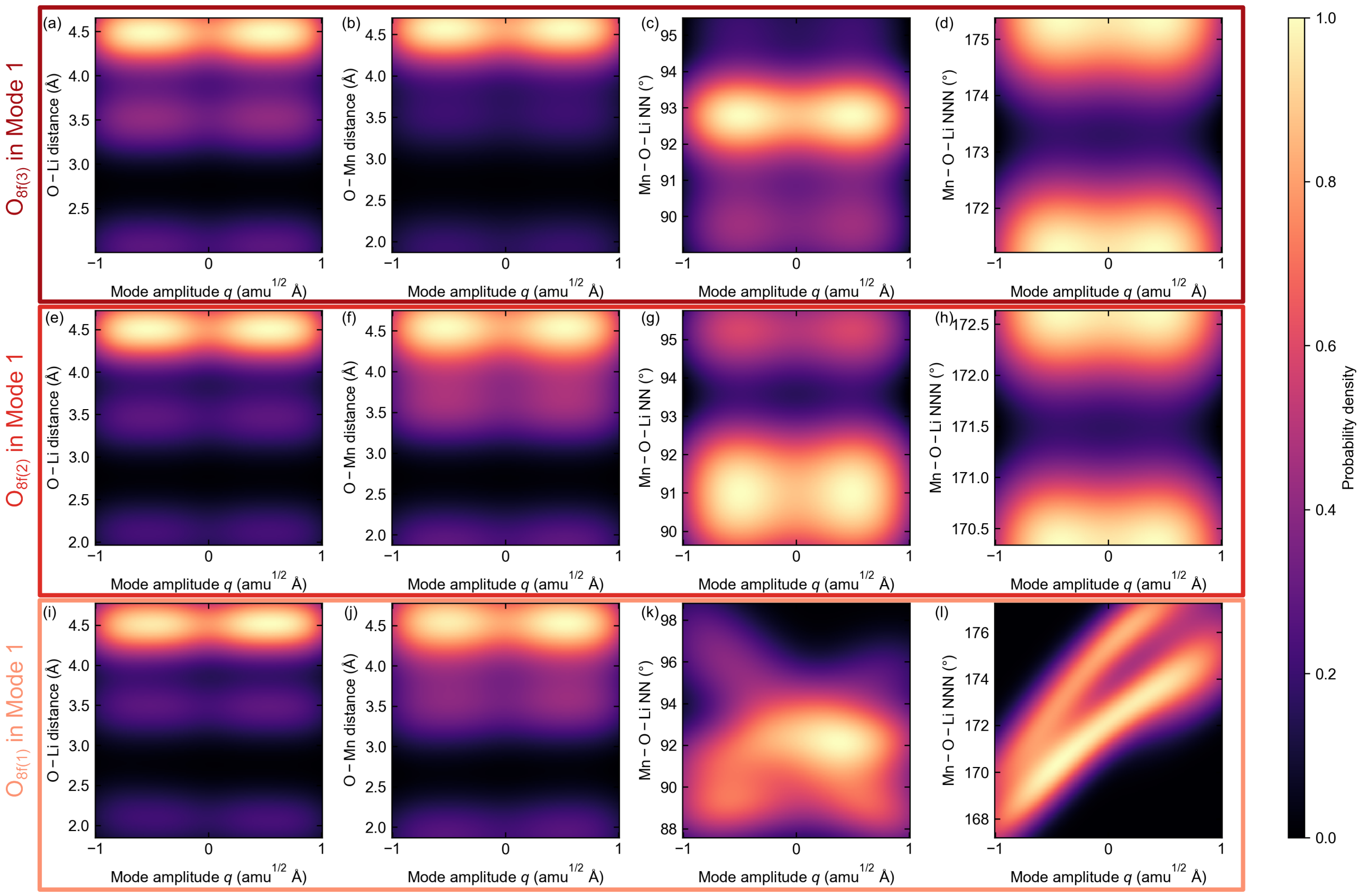

Figure S28: Impact of atomic displacements corresponding to vibrational mode 2 on the local structure around $O_{8f(1)}$ (a) to (d), $O_{8f(2)}$ (e) to (h) and $O_{8f(3)}$ (i) to (l). For each crystallographic O environment, we show the O-Li distances ((a), (e) and (i)), the O-Mn distances ((b), (f) and (j)), the Mn-O-Li nearest-neighbor (NN) bond angle ((c), (g) and (k)) and the Mn-O-Li next-nearest-neighbor (NNN) bond angle ((d), (h) and (l)).

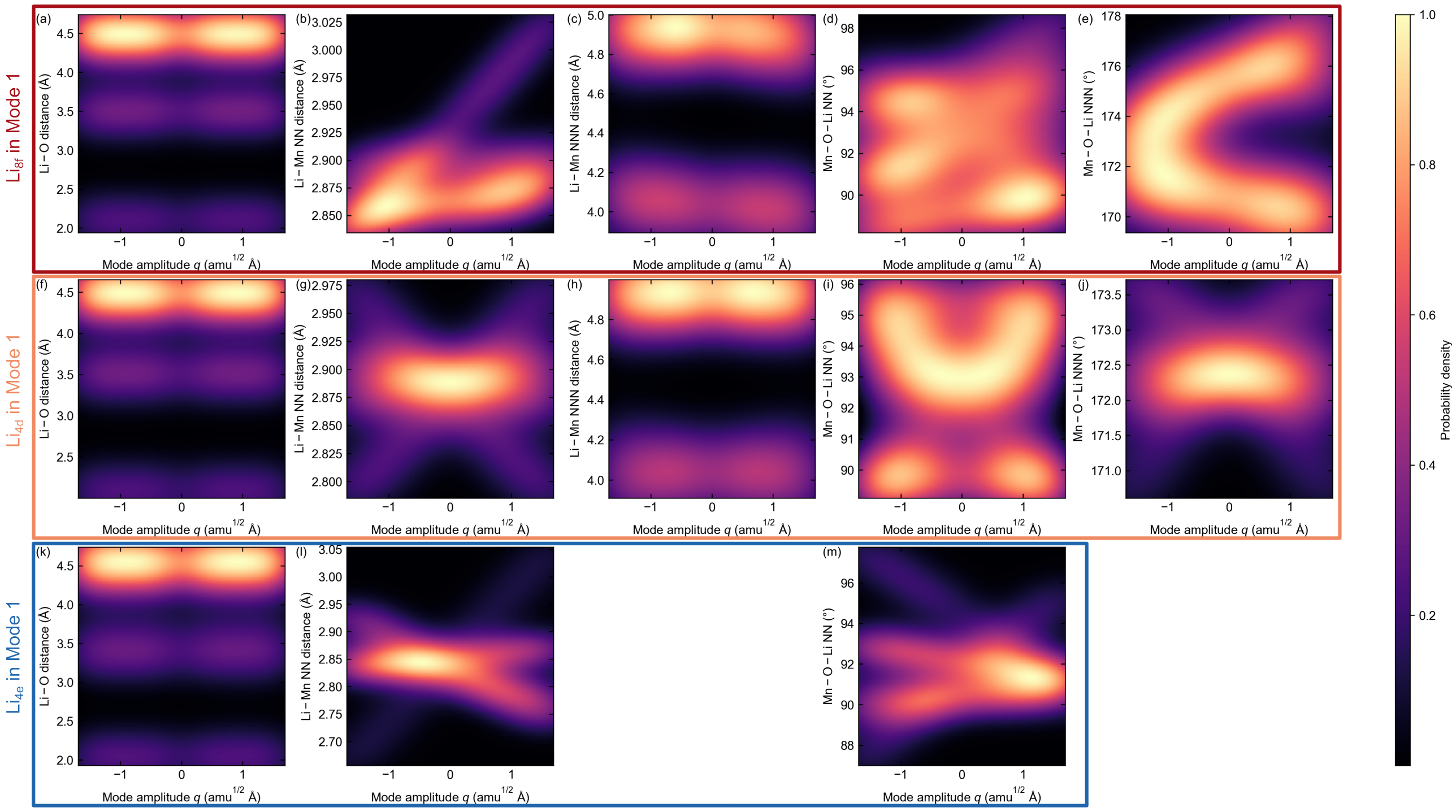

Figure S29: Impact of atomic displacements corresponding to vibrational mode 3 on the local structure around Li_{8f} (a) to (e), Li_{4d} (f) to (j) and Li_{4e} (k) to (m). For each crystallographic Li environment, we show the Li-O distances ((a), (f) and (k)), the Li-Mn nearest-neighbor (NN) distances ((b), (g) and (l)), the Li-Mn next-nearest-neighbor (NNN) distances ((c) and (g) - no such distances were considered for Li_{4e}, as per the magnetic cluster expansion in the main text), the Mn-O-Li NN bond angle ((d), (i) and (m)) and the Mn-O-Li NNN bond angle ((e) and (j), again no such NNN considered for Li_{4e}).

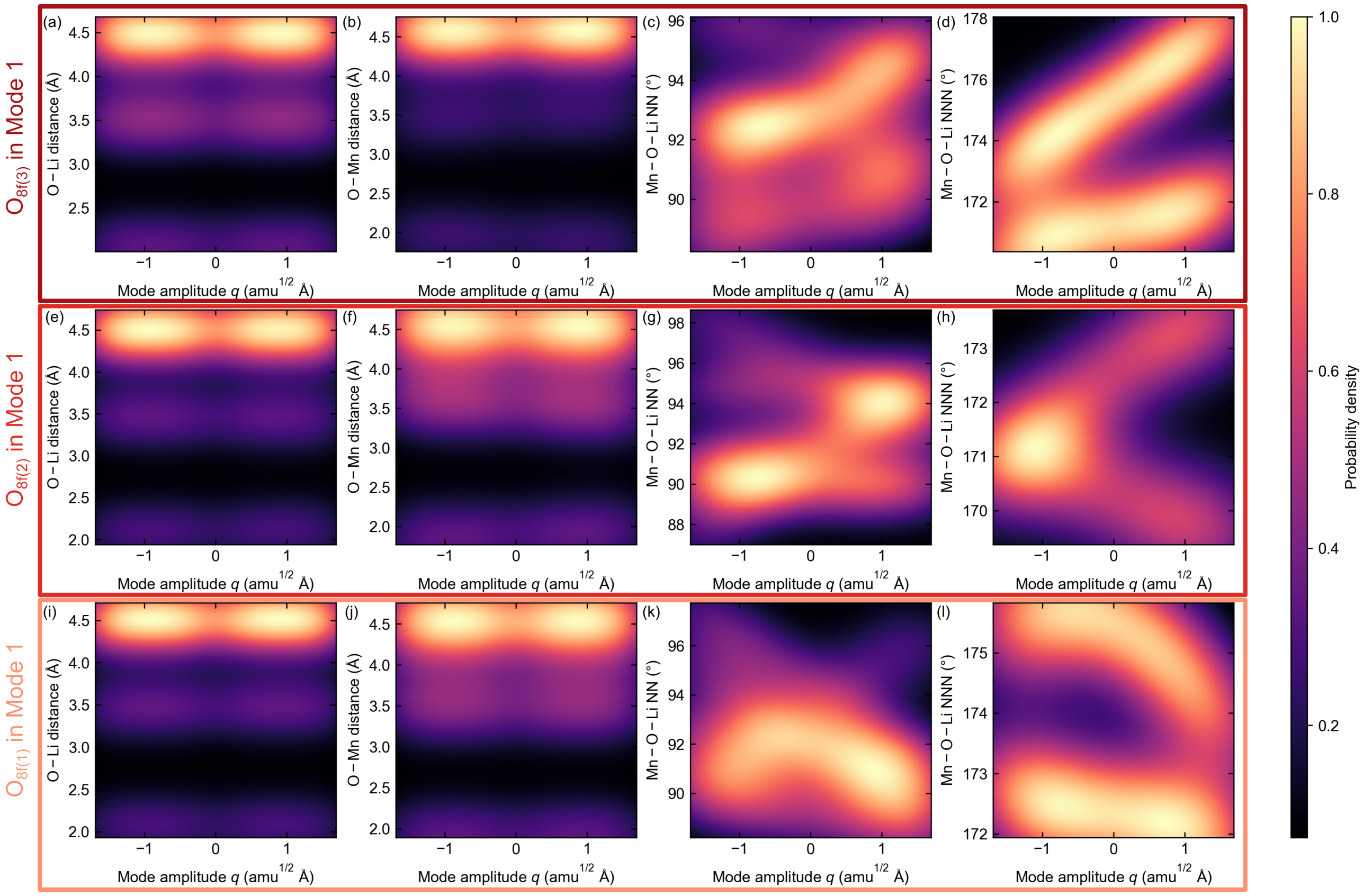

Figure S30: Impact of atomic displacements corresponding to vibrational mode 3 on the local structure around $O_{8f(1)}$ (a) to (d), $O_{8f(2)}$ (e) to (h) and $O_{8f(3)}$ (i) to (l). For each crystallographic O environment, we show the O-Li distances ((a), (e) and (i)), the O-Mn distances ((b), (f) and (j)), the Mn-O-Li nearest-neighbor (NN) bond angle ((c), (g) and (k)) and the Mn-O-Li next-nearest-neighbor (NNN) bond angle ((d), (h) and (l)).

References

(1) Suard, E.; Hewat, A. The Super-D2B project at the ILL: Neutron News: Vol 12, No 4. *Neutron News* **2001**, *12*, 30–33.

(2) Rietveld, H. M. Line profiles of neutron powder-diffraction peaks for structure refinement. *Acta Crystallographica* **1967**, *22*, 151–152.

(3) Rietveld, H. M. A profile refinement method for nuclear and magnetic structures. *Journal of Applied Crystallography* **1969**, *2*, 65–71, Publisher: International Union of Crystallography.

(4) Coelho, A. A. *TOPAS* and *TOPAS-Academic* : an optimization program integrating computer algebra and crystallographic objects written in C++. *Journal of Applied Crystallography* **2018**, *51*, 210–218, Publisher: International Union of Crystallography.

(5) Coelho, A. TOPAS-Academic: General Profile and Structure Analysis Software for Powder Diffraction Data. **2007**, Place: Brisbane, Australia.

(6) Hung, I.; Zhou, L.; Pourpoint, F.; Grey, C. P.; Gan, Z. Isotropic high field NMR spectra of Li-ion battery materials with anisotropy >1 MHz. *Journal of the American Chemical Society* **2012**, *134*, 1898–1901, ISBN: 0002-7863.

(7) Bain, G. A.; Berry, J. F. Diamagnetic Corrections and Pascal's Constants. *J. Chem. Educ.* **2008**, *85*, 532–536.

(8) Kresse, G.; Hafner, J. Ab initio molecular dynamics for liquid metals. *Physical Review B* **1993**, *47*, 558–561, Publisher: American Physical Society.

(9) Kresse, G.; Hafner, J. Ab initio molecular-dynamics simulation of the liquid-metalamorphous- semiconductor transition in germanium. *Physical Review B* **1994**, *49*, 14251–14269, Publisher: American Physical Society.

(10) Kresse, G.; Furthmüller, J. Efficiency of ab-initio total energy calculations for metals and semiconductors using a plane-wave basis set. *Computational Materials Science* **1996**, *6*, 15–50, Publisher: Elsevier.

(11) Blöchl, P. E. Projector augmented-wave method. *Physical Review B* **1994**, *50*, 17953–17979, Publisher: American Physical Society.

(12) Kresse, G.; Joubert, D. From ultrasoft pseudopotentials to the projector augmented-wave method. *Physical Review B* **1999**, *59*, 1758–1775, Publisher: American Physical Society.

(13) Sun, J.; Ruzsinszky, A.; Perdew, J. Strongly Constrained and Appropriately Normed Semilocal Density Functional. *Physical Review Letters* **2015**, *115*, 036402, Publisher: American Physical Society.

(14) Sun, J.; Remsing, R. C.; Zhang, Y.; Sun, Z.; Ruzsinszky, A.; Peng, H.; Yang, Z.; Paul, A.; Waghmare, U.; Wu, X.; Klein, M. L.; Perdew, J. P. Accurate first-principles structures and energies of diversely bonded systems from an efficient density functional. *Nature Chemistry* **2016**, *8*, 831–836, Publisher: Nature Publishing Group.

(15) Peng, H.; Yang, Z. H.; Perdew, J. P.; Sun, J. Versatile van der Waals density functional based on a meta-generalized gradient approximation. *Physical Review X* **2016**, *6*, 041005, Publisher: American Physical Society.

(16) Monkhorst, H. J.; Pack, J. D. Special points for Brillouin-zone integrations. *Physical Review B* **1976**, *13*, 5188–5192, Publisher: American Physical Society.

(17) Puchala, B.; Thomas, J. C.; Natarajan, A. R.; Goiri, J. G.; Behara, S. S.; Kaufman, J. L.; Van der Ven, A. CASM — A software package for first-principles based study of multicomponent crystalline solids. *Computational Materials Science* **2023**, *217*, 111897.

(18) Dovesi, R.; Orlando, R.; Civalleri, B.; Roetti, C.; Saunders, V. R.; Zicovich-Wilson, C. M.

CRYSTAL: A computational tool for the ab initio study of the electronic properties of crystals. *Zeitschrift fur Kristallographie* **2005**, *220*, 571–573, Publisher: De Gruyter.

(19) Erba, A.; Desmarais, J. K.; Casassa, S.; Civalleri, B.; Donà, L.; Bush, I. J.; Searle, B.; Maschio, L.; Edith-Daga, L.; Cossard, A.; Ribaldone, C.; Ascrizzi, E.; Marana, N. L.; Flament, J.-P.; Kirtman, B. CRYSTAL23: A Program for Computational Solid State Physics and Chemistry. *Journal of Chemical Theory and Computation* **2023**, *19*, 6891–6932, Publisher: American Chemical Society.

(20) Dovesi, R.; Erba, A.; Orlando, R.; Zicovich-Wilson, C. M.; Civalleri, B.; Maschio, L.; Rérat, M.; Casassa, S.; Baima, J.; Salustro, S.; Kirtman, B. Quantum-mechanical condensed matter simulations with CRYSTAL. *Wiley Interdisciplinary Reviews: Computational Molecular Science* **2018**, *8*, e1360, Publisher: John Wiley & Sons, Ltd (10.1111).

(21) Clément, R. J.; Pell, A. J.; Middlemiss, D. S.; Strobridge, F. C.; Miller, J. K.; Whittingham, M. S.; Emsley, L.; Grey, C. P.; Pintacuda, G. Spin-Transfer Pathways in Paramagnetic Lithium Transition-Metal Phosphates from Combined Broadband Isotropic Solid-State MAS NMR Spectroscopy and DFT Calculations. *Journal of the American Chemical Society* **2012**, *134*, 17178–17185, Publisher: American Chemical Society.

(22) Middlemiss, D. S.; Ilott, A. J.; Clément, R. J.; Strobridge, F. C.; Grey, C. P. Density Functional Theory-Based Bond Pathway Decompositions of Hyperfine Shifts: Equipping Solid-State NMR to Characterize Atomic Environments in Paramagnetic Materials. *Chemistry of Materials* **2013**, *25*, 1723–1734, Publisher: American Chemical Society.

(23) Kim, J.; Middlemiss, D. S.; Chernova, N. A.; Zhu, B. Y. X.; Masquelier, C.; Grey, C. P. Linking Local Environments and Hyperfine Shifts: A Combined Experimental and Theoretical 31 P and 7 Li Solid-State NMR Study of Paramagnetic Fe(III) Phosphates. *Journal of the American Chemical Society* **2010**, *132*, 16825–16840, Publisher: American Chemical Society.

(24) Kim, J.; Ilott, A. J.; Middlemiss, D. S.; Chernova, N. A.; Pinney, N.; Morgan, D.; Grey, C. P. 2H

and 27Al Solid-State NMR Study of the Local Environments in Al-Doped 2-Line Ferrihydrite, Goethite, and Lepidocrocite. *Chemistry of Materials* **2015**, *27*, 3966–3978.

(25) Becke, A. D. Density-functional thermochemistry. III. The role of exact exchange. *The Journal of Chemical Physics* **1993**, *98*, 5648–5652, Publisher: American Institute of Physics.

(26) Lee, C.; Yang, W.; Parr, R. G. Development of the Colle-Salvetti correlation-energy formula into a functional of the electron density. *Physical Review B* **1988**, *37*, 785–789, Publisher: American Physical Society.

(27) Kutzelnigg, W.; Fleischer, U.; Schindler, M. *The IGLO-Method: Ab-initio Calculation and Interpretation of NMR Chemical Shifts and Magnetic Susceptibilities*; Springer, Berlin, Heidelberg, 1990; pp 165–262.

(28) Lee, J.; Seymour, I. D.; Pell, A. J.; Dutton, S. E.; Grey, C. P. A systematic study of 25 Mg NMR in paramagnetic transition metal oxides: applications to Mg-ion battery materials. *Physical Chemistry Chemical Physics* **2017**, *19*, 613–625, Publisher: The Royal Society of Chemistry.

(29) Catti, M.; Valerio, G.; Dovesi, R.; Causà, M. Quantum-mechanical calculation of the solid-state equilibrium MgO+alpha-Al2O3 <−> MgAl2O4 (spinel) versus pressure. *Physical Review B* **1994**, *49*, 14179–14187, Publisher: American Physical Society.

(30) Catti, M.; Sandrone, G.; Valerio, G.; Dovesi, R. Electronic, magnetic and crystal structure of Cr2O3 by theoretical methods. *Journal of Physics and Chemistry of Solids* **1996**, *57*, 1735–1741, Publisher: Pergamon.

(31) Catti, M.; Sandrone, G.; Dovesi, R. Periodic unrestricted Hartree-Fock study of corundumlike Ti2O3 and V2O3. *Physical Review B* **1997**, *55*, 16122–16131, Publisher: American Physical Society.

(32) Schäfer, A.; Horn, H.; Ahlrichs, R. Fully optimized contracted Gaussian basis sets for atoms

Li to Kr. *The Journal of Chemical Physics* **1992**, *97*, 2571–2577, Publisher: American Institute of Physics.

(33) García Ponte, G. E.; Behara, S. S.; Bassey, E. N.; Clément, R. J.; Van der Ven, A. First-Principles Statistical Mechanics Study of Magnetic Fluctuations and Order–Disorder in the Spinel LiNi0.5Mn1.5O4 Cathode. *Chem. Mater.* **2025**, *37*, 1835–1846, Publisher: American Chemical Society.

(34) Van de Walle, A. A complete representation of structure–property relationships in crystals. *Nature materials* **2008**, *7*, 455–458.

(35) Decolvenaere, E.; Levin, E.; Seshadri, R.; Van der Ven, A. Modeling magnetic evolution and exchange hardening in disordered magnets: The example of Mn 1- x Fe x Ru 2 Sn Heusler alloys. *Physical Review Materials* **2019**, *3*, 104411.

(36) Kitchaev, D. A.; Schueller, E. C.; Van der Ven, A. Mapping skyrmion stability in uniaxial lacunar spinel magnets from first principles. *Physical Review B* **2020**, *101*, 054409.

(37) Thomas, J. C.; Van der Ven, A. The exploration of nonlinear elasticity and its efficient parameterization for crystalline materials. *Journal of the Mechanics and Physics of Solids* **2017**, *107*, 76–95.
